# Microglial GPR35 Ameliorates Epileptogenesis and Neuroinflammation via PDGFA Domain 2 Signaling

**DOI:** 10.1002/advs.202519642

**Published:** 2026-01-28

**Authors:** Qi Wang, Tingting Qu, Qibing Sun, Ran Li, Junfei Dong, Yuming Du, Ziyin Xuan, Lei Wang, Hanli Li, Jianyun Sun, Fangliang Chen, Jinshuai Liu, Zifan Yang, Jianxiang Lei, Qian Yang, Bin Wang, Zhiming Zhou, Yu Wang

**Affiliations:** ^1^ Department of Neurology, Epilepsy and Headache Group The First Affiliated Hospital of Anhui Medical University Hefei China; ^2^ Department of Neurology, First Affiliated Hospital of Wannan Medical College Yijishan Hospital Wuhu Anhui China; ^3^ Department of Neurology Anhui Public Health Clinical Center Hefei China; ^4^ Department of Anesthesiology, First Affiliated Hospital of Wannan Medical College Yijishan Hospital Wuhu Anhui China

**Keywords:** epileptogenesis, GPR35, kynurenic acid, neuroinflammation, PDGFA

## Abstract

Neuroinflammation is a critical driver of epileptogenesis and cognitive dysfunction in epilepsy; however, targeted anti‐inflammatory therapies remain limited. In this study, we demonstrate that microglial GPR35 orchestrates neuroinflammatory epileptic networks through platelet‐derived growth factor A (PDGFA)‐dependent signaling. Single‐nucleus RNA sequencing of patients with temporal lobe epilepsy (TLE) and pharmacological models reveals selective GPR35 upregulation in disease‐associated microglia. GPR35 deficiency exacerbates seizure susceptibility and cognitive deficits. We further demonstrate that GPR35 activation mitigates seizures, suppresses hippocampal neuroinflammation, and alleviates cognitive deficits. Mechanistically, kynurenic acid–activated GPR35 specifically interacts with PDGFA domain 2 via defined binding motifs, thereby suppressing PDGFA degradation through the ubiquitin–proteasome pathway. This cascade triggers PI3K–AKT signaling and subsequently inhibits pro‐inflammatory responses. Conversely, GPR35 deficiency disrupts this pathway of neuroinflammation, and hyperexcitability. PDGFA overexpression phenocopies GPR35 activation, attenuating inflammation and epileptogenesis. These findings establish GPR35 as a critical modulator of epileptic networks via PDGFA‐dependent anti‐inflammatory signaling, bridging neuroimmune crosstalk with the pathophysiology of epilepsy. Our study identifies GPR35 as a druggable target capable of disrupting the vicious cycle of inflammation and hyperexcitability in epilepsy, offering a dual therapeutic strategy to alleviate seizures and cognitive comorbidities.

## Graphical abstract

Activation of microglial G protein–coupled receptor 35 (GPR35) by L‐kynurenic acid (L‐Kyna) initiates a platelet‐derived growth factor A (PDGFA)–dependent phosphoinositide 3‐kinase–protein kinase B (PI3K–AKT) signaling cascade that dampens hippocampal neuroinflammation, thereby restraining epileptogenesis, lowering seizure susceptibility, and ameliorating epilepsy‐related cognitive dysfunction.

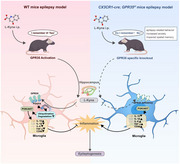

## 1 Introduction

Epilepsy is the second most common neurological disorder after stroke, with approximately 2.4 million new diagnoses each year. Despite the availability of more than 20 anti‐epileptic drugs, seizures remain uncontrolled in approximately 30% of patients [1], with limited treatment options beyond surgery. Most patients with drug‐resistant epilepsy are not candidates for surgical intervention [2]. Data indicate that 80% of patients treated with medication experience adverse reactions, and 30%–40% experience reactions that significantly affect the quality of life, leading to drug withdrawal or non‐compliance and failing to improve the long‐term prognosis [3]. In addition, the mechanisms of action of current anti‐epileptic drugs are poorly correlated with their clinical efficacy spectrum, and the introduction of antiepileptic drugs with novel mechanisms has not reduced the frequency of drug‐resistant epilepsy [4]. Therefore, exploring the pathogenesis of epilepsy and identifying new treatment strategies remain urgent clinical priorities.

Accumulating evidence demonstrates that neuroinflammatory processes play a fundamental role in epileptogenesis and seizure generation [5]. Basic research and clinical studies have consistently shown that increased concentrations of pro‐inflammatory mediators such as interleukin (IL)‐1β, tumor necrosis factor (TNF)‐α, and IL‐6 contribute to epileptogenesis through multiple mechanisms, including blood‐brain barrier disruption, neuronal hyperexcitability, and impaired synaptic plasticity [5, 6]. We have also observed alterations in peripheral immune cells in patients with chronic epilepsy, which strongly correlate with seizure recurrence, highlighting the role of chronic inflammation in recurrent seizure activity [7]. Novel anti‐neuroinflammatory drugs targeting specific inflammatory cells or molecular pathways have been developed and are anticipated to overcome the limitations of current anti‐epileptic drugs that lack disease‐modifying effects [8]. Thus, chronic neuroinflammation persisting throughout epileptogenesis constitutes a critical contributor to sustained hyperexcitability in epileptic brains, and further mechanistic investigations and targeted interventions are essential for developing innovative therapeutic strategies.

GPR35, a G protein‐coupled receptor (GPCR), is activated by endogenous kynurenic acid (KYNA) [9] and regulates inflammatory responses [10]. Emerging evidence indicates that GPR35 is significantly upregulated in activated neutrophils, enhancing their migratory capacity through chemotactic signaling pathways [11]. Furthermore, this receptor contributes to macrophage polarization by promoting TNF‐α production [12]. Murine models with conditional GPR35 deficiency in CX3CR1^+^ macrophages display exacerbated inflammatory responses under pathological conditions, suggesting a regulatory role in myeloid cell‐mediated immunity [12]. In lipopolysaccharide (LPS)‐induced microglial inflammation models, GPR35 activation suppresses NLRP3 inflammasome assembly, reduces IL‐1β and IL‐18 secretion, and enhances anti‐inflammatory IL‐10 production [13]. In experimental murine models of autoimmune encephalomyelitis, GPR35 knockout exacerbates Th17 cell infiltration and blood‐brain barrier disruption, highlighting its anti‐neuroinflammatory potential [14]. Astrocytic GPR35 regulates ERK1/2 and NF‐κB signaling, influencing glial scar formation and synaptic remodeling, with dysregulation linked to neurodegenerative diseases such as Alzheimer's and Parkinson's disease [15]. However, the expression patterns and roles of GPR35 in epilepsy remain unclear. Using quantitative proteomic analysis, we identified platelet‐derived growth factor A (PDGFA)‐a factor classically associated with development and angiogenesis. The PDGF family has emerged as a critical modulator of neuroinflammation, exhibiting context‐dependent functions that can either exacerbate or ameliorate pathology [16]. PDGFA signaling primarily through its receptor PDGFRα and is vital for oligodendrocyte progenitor cell homeostasis and angiogenesis [17]. PDGFA expression is upregulated in neuroinflammatory contexts, and astrocyte‐derived PDGFA potently drives microglial activation, contributing to neuroinflammatory cascades [18]. Beyond its immunomodulatory role, PDGFA signaling maintains central nervous system homeostasis, and its dysregulation is implicated in neuroinflammatory pathogenesis [19]. However, the extent to which PDGFA interacts with GPCR signaling, specifically, how it functionally interacts with microglial GPR35 in epilepsy, remains unclear.

In this study, we systematically investigated GPR35 expression in brain tissue from patients with epilepsy, evaluated its expression profiles across various experimental models, and explored the role of GPR35 in kainic acid (KA)‐induced seizures and associated mechanisms in mice. We found that GPR35 expression is upregulated in the epileptic brains of patients and animal models. Moreover, we demonstrate that GPR35 engages with PDGFA domain 2 via specific binding motifs, inhibiting its ubiquitin proteasome‐mediated degradation, enhancing its phosphorylation, and activating PI3K‐AKT signaling. This pathway attenuates neuroinflammatory responses and reduces seizure activity. Our findings identify​ GPR35 as a novel, druggable target for anti‐epileptic therapy aimed at disrupting the neuroinflammation hyperexcitability cycle.

## 2 Results

### 2.1 Expression of GPR35 in Patients with Temporal Lobe Epilepsy (TLE) and In Vivo and In Vitro Epileptic Models

**FIGURE 1:**
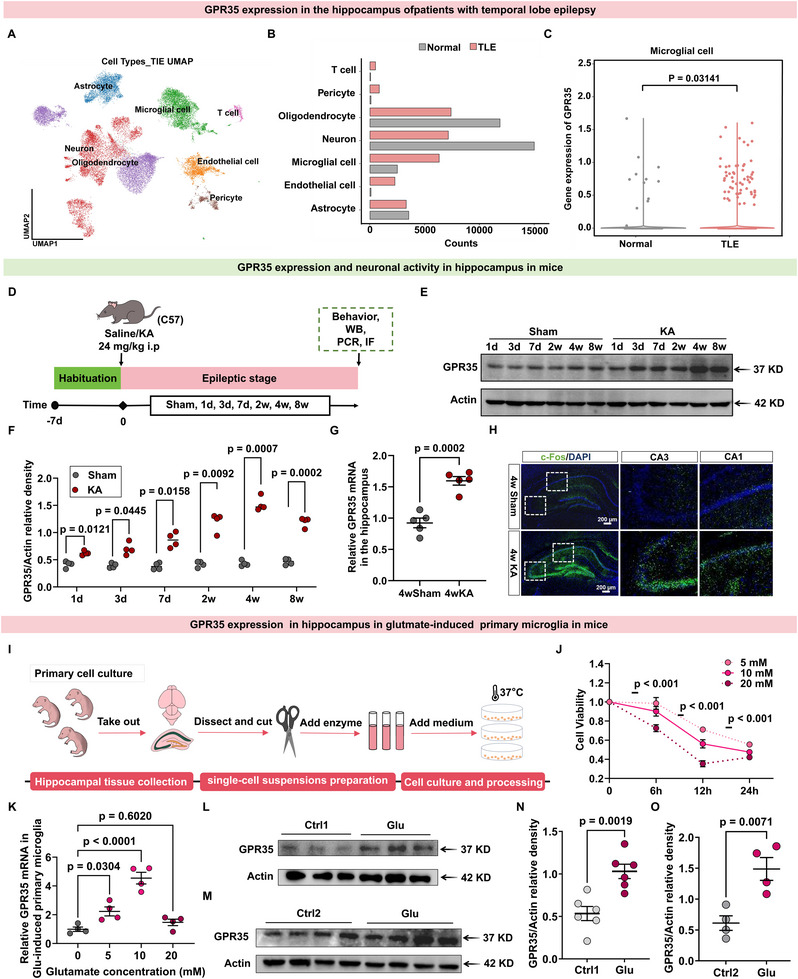
Expression of GPR35 in serum from patients with epilepsy, a kainic acid‐induced (KA‐induced) in vivo epileptic model, and a glutamate‐induced (Glu‐induced) in vitro epileptic model. (A) Unsupervised clustering analysis of distinct cell populations. Single‐nucleus RNA sequencing (snRNA‐seq) analysis of patients with temporal lobe epilepsy (TLE) and non‐epileptic controls (GSE140393, GSE190452). (B) Histogram depicting GPR35 expression levels across different cell types, presented as the percentage of positive cells. Student's *t* test was used for analysis. (C) Comparative analysis of microglial GPR35 gene expression between patients with TLE and non‐epileptic controls. Student's *t* test was used for analysis. (D) Schematic diagram of the experimental procedure. (E,F) Representative Western blot (E) and quantification (F) of hippocampal GPR35 protein levels in mice at various time points following KA‐induced seizures or sham procedure (n = 4). Two‐way ANCOVA was used for analysis. (G) Quantitative real‐time PCR (qRT‐PCR) analysis of hippocampal GPR35 mRNA expression in mice subjected to 4‐week KA treatment (4wKA) or sham procedure (4wsham) (n = 5). One‐way analysis of covariance (ANCOVA) was used for analysis. (H) Immunofluorescence microscopy images of c‐Fos (green) in hippocampal sections from 4wsham and 4wKA mice. Scale bars = 200 µm (n = 3). (I) Schematic representation of the primary microglial culture procedure. (J) Cell Counting Kit‐8 (CCK‐8) assay demonstrating Glu‐induced dose‐ and time‐dependent reductions in neuronal viability (n = 3). One‐way ANCOVA was used for analysis. (K) qRT‐PCR analysis of GPR35 mRNA levels in primary microglia exposed to varying glutamate concentrations (n = 4). One‐way ANCOVA was used for analysis. (L,N) Representative Western blot (L) and quantification (N) of GPR35 protein levels in BV2 cells treated with 10 mm glutamate for 12 h compared with untreated controls (n = 6). Two‐tailed unpaired *t* test was used for analysis. (M,O) Representative Western blot (M) and quantification (O) of GPR35 protein levels in primary microglia treated with 10 mm glutamate for 12 h compared with untreated controls (n = 4). Two‐tailed unpaired *t* test was used for analysis. Data are presented as mean ± standard error of the mean (SEM).

Neuroinflammation is one of the common pathogenic mechanisms underlying epileptic seizures [5]. Previous studies have demonstrated that GPR35 can regulate tissue energy homeostasis and inflammation [20] through its endogenous ligand, kynurenic acid (Kyna), which plays a critical role in epilepsy‐associated inflammation [21]. Therefore, we hypothesized that GPR35 is a key regulatory protein involved in the neuroinflammatory processes during epileptogenesis. To determine the relevance of GPR35 in seizures, we first used publicly available human single‐nucleus RNA sequencing (snRNA‐seq) datasets to characterize GPR35 distribution in the hippocampus of patients with temporal lobe epilepsy (TLE) (Figure s1 A). Clustering analysis identified nine major cell types using representative marker genes (Figure 1A; Figure S1D). Cell population analysis revealed substantial differences between TLE and non‐TLE controls, including neuronal loss and increased microglial numbers in TLE participants (Figure 1B). Among these, GPR35 was significantly upregulated in microglia of TLE participants, but not in other cell types (Figure 1C; Figure S1B,C)

Next, we investigated changes in GPR35 expression in epilepsy models. Experimental induction of epilepsy in mice was achieved with KA [22], whereas the sham group received an intraperitoneal injection of an equivalent volume of normal saline. The acute seizure phase was evaluated using the Racine scale, and only animals that exhibited stage IV–VI seizures were included in subsequent analyses (Figure S2A). We set five time points for the assessment of GPR35 in each group: 3 days, 1 week, 2 weeks, 4 weeks, and 8 weeks after KA induction (4wKA) or after sham (4wSham) (Figure 1D). Western blot analysis showed that GPR35 abundance gradually increased until 4wKA but decreased by 8wKA (Figure 1E,F; Table S10). At the 4‐week time point, KA‐induced epileptic mice exhibited upregulated expression of pro‐inflammatory cytokines (IL‐1β, IL‐6, TNF‐α), alongside downregulated expression of the anti‐inflammatory cytokine IL‐10, compared with sham mice (Figure S2B). GPR35 mRNA in hippocampal tissue was also markedly increased at 4wKA (Figure 1G; Table S11), and its abundance was positively correlated with pro‐inflammatory factor mRNA (Figure S2C–E), but negatively correlated with anti‐inflammatory factors (Figure S2F). Consistent with the snRNA‐seq results, immunofluorescence staining of hippocampal samples showed higher GPR35 protein expression in microglia (Figure S3), but not in neurons, in KA‐induced mice than in sham mice. GPR35 activation was accompanied by increased levels of c‐Fos, a marker of neuronal activation (Figure 1H).

We performed a temporal analysis of GPR35 protein dynamics in a glutamate‐induced (Glu‐induced) cell model (Figure 1I). The time‐dependent effects of varying glutamate concentrations (5, 10, and 20 mm) on neural cell viability were evaluated using a Cell Counting Kit‐8 assay. Glutamate exposure induced dose‐ and time‐dependent suppression of neural cell viability. Significant reductions were observed as early as 6 h, and high‐concentration glutamate (20 mm) caused an approximately 60% loss of cell viability within 24 h (Figure 1J; Table S12). Polymerase chain reaction (PCR) analysis revealed that GPR35 mRNA expression peaked after 12 h of induction with 10 mm glutamate, paralleling the upregulation of pro‐inflammatory factors (IL‐1β and TNF) (Figure 1K; Figure S4, Table S13). Consequently, we selected the 10 mM Glu‐induced 12 h model for subsequent experimental investigations. Western blot analysis confirmed that GPR35 protein levels were significantly increased in the model group compared with the control group in the BV2 cell‐induced model (Figure 1L,N; Table S14), with similar results in the primary microglia‐induced model (Figure 1M,O; Table S15). These findings indicate a correlation between elevated GPR35 levels and seizures in humans, mice, and Glu‐induced in vitro models.

**FIGURE 2:**
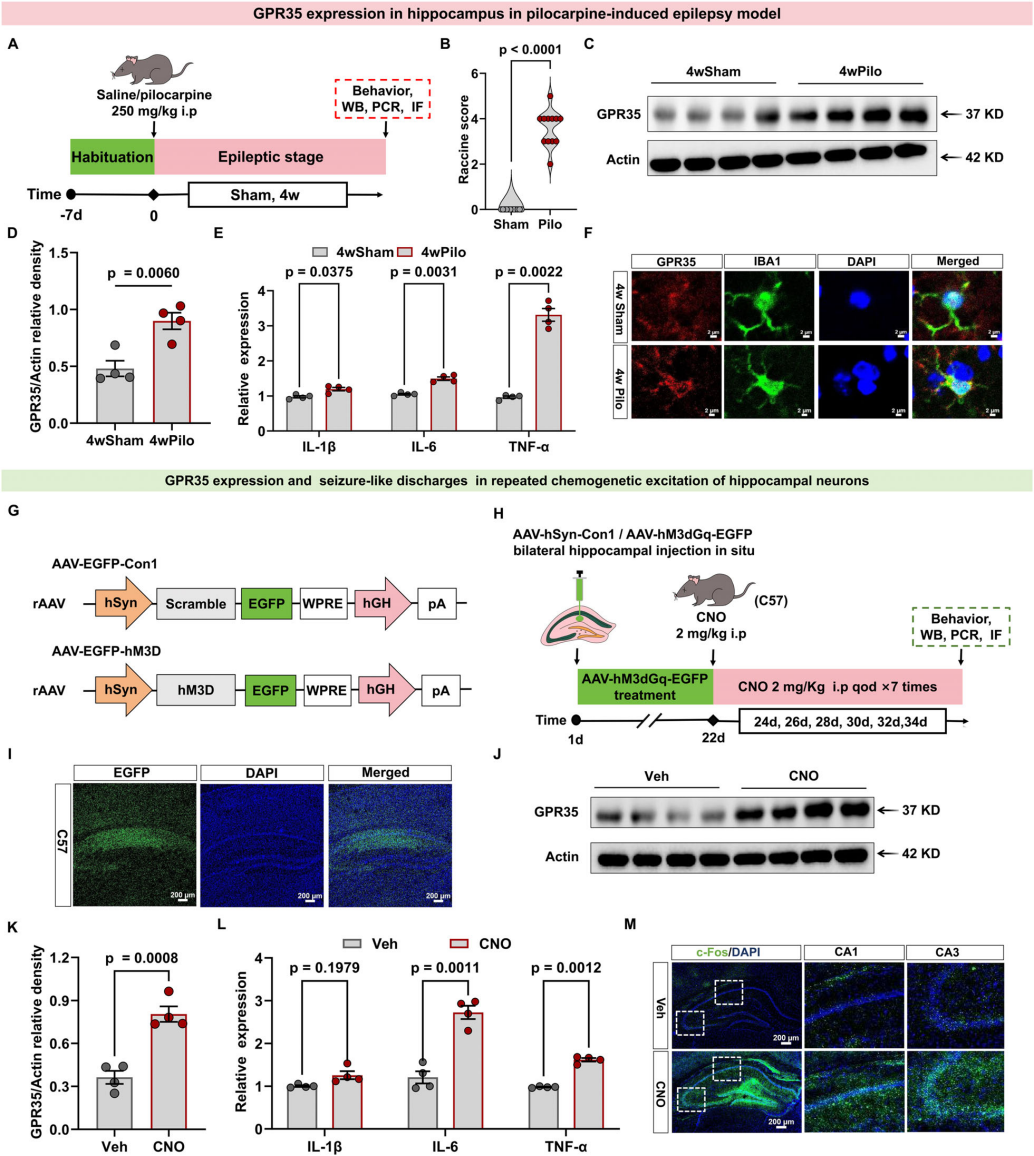
GPR35 expression in different epilepsy models. (A) Schematic diagram of the experimental procedure. (B) Behavioral seizures were evaluated at the acute stage using the Racine scale (Sham, n  =  12; KA, n  =  12). One‐way ANCOVA was used for analysis. (C,D) Representative Western blot (C) and quantification (D) of hippocampal GPR35 protein levels in mice at 4‐week time points following pilocarpine‐induced seizures or sham (n = 4). One‐way ANCOVA was used for analysis. (E) Quantitative real‐time PCR (qRT‐PCR) analysis of hippocampal inflammatory cytokines mRNA (interleukin [IL]‐1β, IL‐6, tumor necrosis factor [TNF]‐α, and IL‐10) mRNA expression in mice subjected to 4‐week pilocarpine treatment (4wPilo) or sham (4wSham) (n = 4). One‐way ANCOVA was used for analysis. (F) Immunofluorescence staining showing GPR35 expression in microglia in 4wSham and 4wPilo mice. Scale bar = 20 µm (n = 3). (G) Schematic of AAV vectors for neuronal activation. (H) Schematic diagram of the experimental procedure. (I) Representative images of EGFP expression 21 days after AAV‐hM3D in the hippocampus of wild‐type (WT) mice (n = 3). Scale bar = 200 µm. (J,K) Representative Western blot (J) and quantification (K) of hippocampal GPR35 protein levels in mice at Day 14 following repeated chemogenetic excitation of hippocampal neurons induced seizure‐like or sham (n = 4). One‐way ANCOVA was used for analysis. (L) qRT‐PCR analysis of hippocampal inflammatory cytokines mRNA (IL‐1β, IL‐6, TNF‐α, and IL‐10) mRNA expression in mice subjected to repeated chemogenetic excitation of hippocampal neurons induced seizure‐like (red) or sham stimulation (gray) (n = 4). Two‐way ANCOVA was used for analysis. (M) Immunofluorescence images of c‐Fos (green) expression in hippocampal sections from chemogenetic excitation or sham (n = 3). Scale bars = 200 µm. Two‐way ANCOVA was used for analysis. Data are presented as mean ± standard error of the mean (SEM).

We also investigated whether changes in GPR35 levels in KA‐induced epilepsy models were reproduced in other epilepsy models. As expected, GPR35 was highly expressed in pilocarpine‐induced (Pilo‐induced) epilepsy models (Figure 2A–F; Tables S16–S18) and after repeated chemogenetic excitation of hippocampal neurons (Figure 2G–M; Tables S19 and S20). These findings suggest that the time‐dependent upregulation of GPR35 in hippocampal tissue following epilepsy is independent of the method of seizure induction.

### 2.2 The Absence of GPR35 Exacerbates the Severity of Chronic Seizures in Epileptic Mice

To determine whether GPR35 is functional in the hippocampus of 4wKA mice, we generated global knockout GPR35 (GPR35^KO^) mice to validate the role of GPR35 in epileptogenesis. The GPR35 knockout mouse model was generated by Shanghai Model Organisms Center, Inc. (Shanghai, China) using CRISPR/Cas9 technology. Four sgRNAs targeting exon 3 of the GPR35 gene were designed and in vitro transcribed using the MEGAshortscript Kit (Thermo Fisher, USA), then co‐injected with in vitro‐transcribed Cas9 mRNA into C57BL/6J zygotes, which were subsequently transferred to pseudopregnant recipients. The resulting F0 founders were identified by PCR genotyping, validated by Sanger sequencing, and backcrossed with wild‐type (WT) C57BL/6J mice to generate F1 heterozygotes, which were then intercrossed to produce homozygous GPR35 knockout mice with genotypes confirmed by PCR and sequencing (Figure S5A,B; Table S1). The knockout had no significant effects on body weight or brain morphology (Figure S5C–F).

**FIGURE 3:**
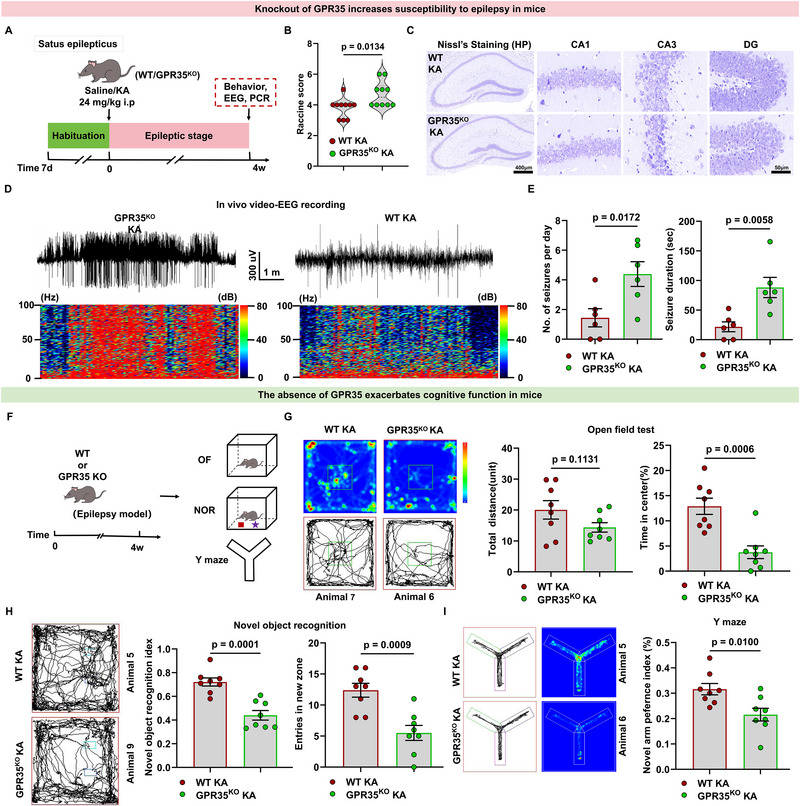
Loss of GPR35 expression exacerbated epilepsy and impaired cognition in mice. (A) Experimental design for studying the impact of GPR35 knockout (GPR35^KO^) on KA‐induced SE. (B) Behavior seizures of KA‐induced WT mice and GPR35^KO^ mice were evaluated at the acute stage using the Racine scale (n = 10). Two‐tailed unpaired *t* test was used for analysis. (C) Nissl staining demonstrating neuronal loss in hippocampal CA1 of GPR35^KO^ KA group compared with Shams (n = 3). (D,E) Electrographic seizure‐like activities were assessed at the chronic stage by in vivo EEG (n = 6). Two‐tailed unpaired *t* test was used for analysis. (F) Schematic of treatments and the cognitive testing timeline (i.p., intraperitoneal injection; OP, the open‐field test; NOR, novel object recognition. (G) (Left) Representative traces of 4wKA WT and GPR35^KO^ mice in the open field arena. (Right) Quantification of total distance traveled and percentage of time in the center (n = 8). Two‐tailed unpaired *t* test was used for analysis. (H) (Left) Representative traces during NOR. The blue rectangle indicates the novel object. (Right) Quantification of the percentage of time exploring the novel object (n = 8). Two‐tailed unpaired *t* test was used for analysis. (I) (Left) Representative traces during Y maze test. (Right) Quantification of time spent in the novel arm (n = 8). One‐way ANCOVA was used for analysis. Data are presented as mean ± standard error of the mean (SEM).

To test whether loss of GPR35 affects seizure severity, we subjected GPR35^KO^ and WT mice to the KA model (Figure 3A). Compared with WT KA mice, GPR35^KO^ KA mice exhibited higher Racine scores (Figure 3B; Table S21), more pronounced neuronal loss (Figure 3C; Figure S6A), and higher expression of pro‐inflammatory factors (Figure S6B). Surface electroencephalographic (EEG) recordings showed increased seizure severity at 4wKA compared with WT mice (Figure 3D,E; Table S22). These results indicate that GPR35 is involved in the neuroinflammatory pathophysiology of KA‐induced epilepsy at 4wKA. Collectively, these findings demonstrate that GPR35 deletion increases the severity of chronic seizures, indicating its involvement in the pathogenesis of epilepsy.

### 2.3 The Absence of GPR35 Aggravates Epilepsy‐Related Cognitive Impairment in KA‐Induced Epilepsy

We also investigated the effects of GPR35 deficiency on cognitive functions in epilepsy. To understand how GPR35 affects epilepsy‐related cognition, we used WT and GPR35^KO^ mice to establish a KA‐induced epilepsy model, followed by a series of behavioral experiments (Figure 3F). Hippocampal‐dependent learning and memory were assessed using the novel object recognition (NOR) and Y‐maze tests, whereas anxiety‐ and depression‐like behaviors were evaluated using the open‐field test (OFT).

In the OFT, mice were allowed to explore the arena for 10 min. GPR35^KO^‐epileptic mice were reluctant to explore the central area and spent less time in the center than control epileptic mice, although both groups traveled comparable distances. Reduced time in the central area is associated with higher anxiety‐ and depression‐like behavior, suggesting that GPR35 deficiency aggravates these behaviors in epileptic mice (Figure 3G; Table S23). During the NOR test, mice freely explored a novel and a familiar object in the arena for 10 min. In the Y maze test, mice were allowed free movement from the start arm to the other two arms for 10 min. Compared with control epileptic mice, GPR35^KO^ epileptic mice exhibited reduced preference for the novel object (NOR) and novel arm (Y maze) relative to the familiar condition (Figure 3H,I; Tables S24 and S25). These results indicated that GPR35^KO^ epileptic mice have impaired spatial working memory and that GPR35 deficiency aggravated spatial memory impairment in epilepsy. Together, these findings demonstrate that GPR35 knockout exacerbates cognitive impairments in KA‐induced epileptic mice, manifested as reduced exploratory behavior, diminished object recognition, and impaired spatial memory, suggesting a neuroprotective role for GPR35 in epilepsy‐associated behavioral deficits.

### 2.4 GPR35 Exhibits Cell‐Type‐Specific Effects on Seizure Susceptibility

**FIGURE 4:**
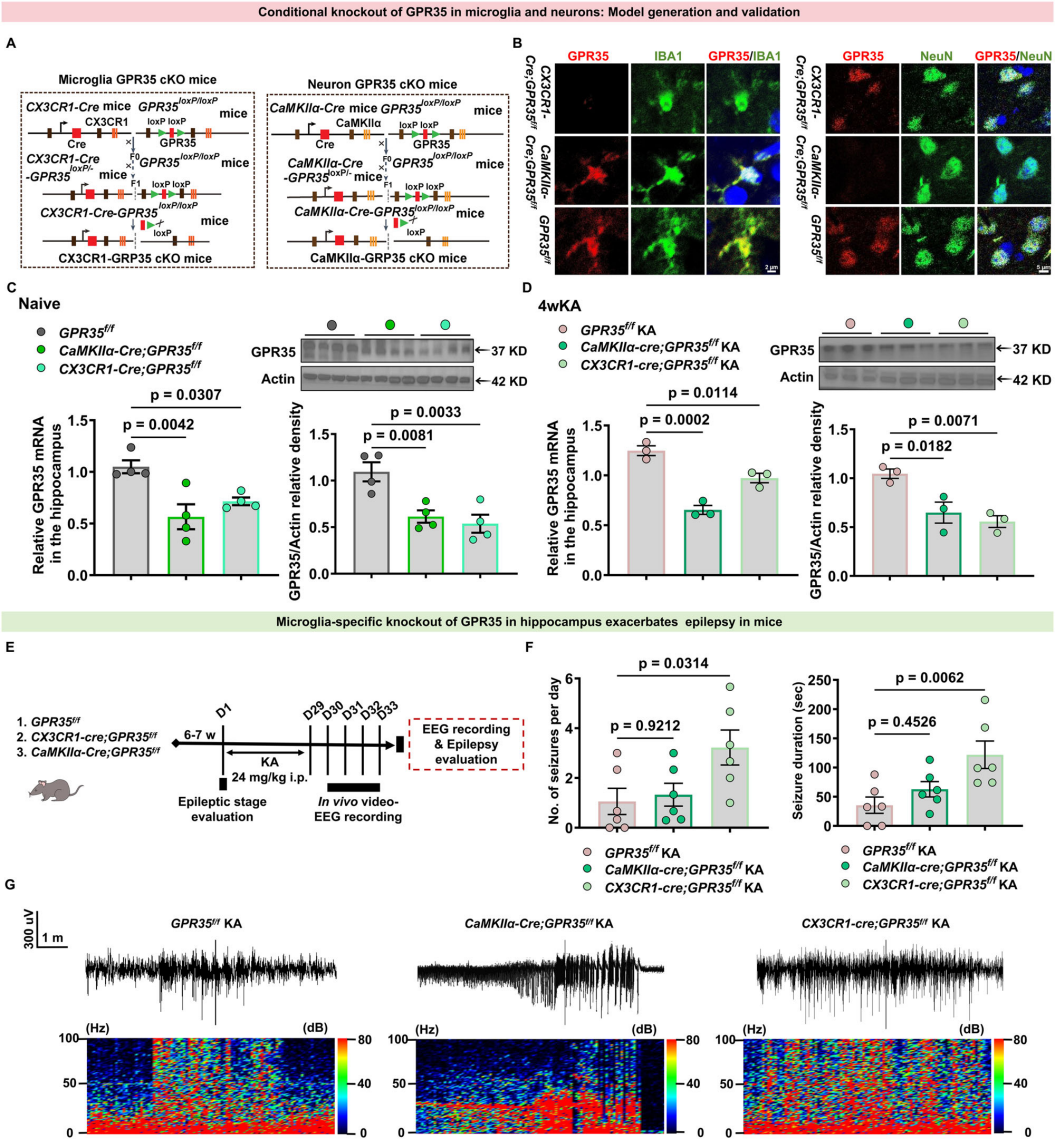
Cell type‐specific effects of GPR35 on seizures. (A) Specific knockout of GPR35 in microglia was achieved by crossing *CX3CR1‐Cre* mice and *CaMKIIα‐Cre* mice with *GPR35‐loxP* mice. (B) Co‐localization of GPR35 (green) with Iba‐1 (or NeuN, red) in the hippocampus (CA1 region) of *CX3CR1‐Cre;GPR35^f/f^* mice (or *CaMKIIα‐Cre;GPR35^f/f^*) 4 weeks post‐kainic acid (KA) (n = 3). Scale bar = 2 and 5 µm. (C) Hippocampal GPR35 mRNA and protein (n = 4 in naive conditions) and (D) 4 weeks post‐KA (n = 3). One‐way ANCOVA was used for analysis. (E) Diagram illustrating the timeline of KA injection treatment (24 mg/kg, i.p.) and the evaluation of seizure symptoms in *GPR35^f/f^*, *CaMKIIα‐Cre;GPR35^f/f^*, and *CX3CR1‐Cre;GPR35^f/f^* mice. (F,G) Electrographic seizure‐like activities were assessed at the chronic stage by in vivo EEG (n = 6). Data are presented as mean ± standard error of the mean (SEM).

To test whether cell type‐specific loss of the GPR35 impacts seizure severity, we generated two conditional knockout mouse models on a C57BL/6J background: (1) microglia‐specific (*CX3CR1‐Cre;GPR35^f/f^*) and (2) hippocampal pyramidal neuron‐specific (*CaMKIIα‐Cre;GPR35^f/f^*) deletions (Figure 4A; Tables S2 and S3). The *GPR35^f/f^* line was generated using CRISPR‐Cas9 to flank exon 3 with LoxP sites (Shanghai Model Organisms Center). For conditional knockout, *GPR35^f/f^* mice were crossed with *CX3CR1‐Cre* (microglia‐specific) or *CaMKIIα‐Cre* (neuron‐specific) mice. Age‐ and sex‐matched *GPR35^f/f^* littermates without Cre were used as controls. Control experiments confirmed no significant differences in body weight, brain weight, or water content between genotypes (Figure S7). Mice underwent a 6‐week pre‐conditioning period under standardized SPF conditions prior to experimentation. These models enabled precise determination of whether the epileptogenic effects of GPR35 are mediated by hippocampal pyramidal neurons, microglia, or both.

GPR35 deficiency in microglia was first verified by co‐immunostaining. Using a GPR35 antibody together with the microglial marker Iba1, we observed a complete absence of GPR35 in microglia of *CX3CR1‐Cre;GPR35^f/f^* mice, whereas GPR35 expression remained detectable in neurons. In contrast, microglia from *CaMKIIα‐Cre;GPR35^f/f^* mice retained robust GPR35 immunoreactivity. Analysis of the CA1 region in *CaMKIIα‐Cre;GPR35^f/f^* mice confirmed efficient neuronal deletion, whereas GPR35 remained expressed in microglia (Figure 4B). Notably, qPCR and Western blot analyses showed that total hippocampal GPR35 mRNA and protein levels were reduced by approximately 20%–40% in both *CX3CR1‐Cre;GPR35^f/f^* and *CaMKIIα‐Cre;GPR35^f/f^* mice under baseline conditions and 4 weeks after SE (Figure 4C,D; Tables S26 and S27), consistent with the expected cell type‐specific knockout.

To determine whether GPR35 expression in microglia or neurons contributes to epileptogenesis, *CaMKIIα‐Cre;GPR35^f/f^*, *CX3CR1‐Cre;GPR35^f/f^*, and *GPR35^f/f^* control mice were subjected to KA (Figure 4E). Behavioral seizures were evaluated in the acute phase using Racine scale scoring, and electrographic seizure activity in the chronic phase (4 weeks) was assessed by in vivo EEG recordings. Compared with *GPR35^f/f^* controls, surface EEG recordings showed that *CX3CR1‐Cre;GPR35^f/f^* mice displayed markedly exacerbated seizure activity compared with both *CaMKIIα‐Cre;GPR35^f/f^* and *GPR35^f/f^* mice, while *CaMKIIα‐Cre;GPR35^f/f^* mice exhibited only a modest trend toward increased spontaneous seizure frequency (Figure 4F,G; Table S28). Specifically, *CX3CR1‐Cre;GPR35^f/f^* mice exhibited a significant increase in daily seizure frequency as well as a prolonged duration of individual seizures. These results indicate that neuronal loss of GPR35 produces only a mild trend toward increased seizure susceptibility, whereas microglia‐specific GPR35 deletion leads to a substantially more severe epileptic phenotype. Thus, GPR35 knockout exerts cell type‐dependent, with the strongest effects observed in microglia‐specific knockouts. Collectively, the loss of microglial GPR35 rendered mice more susceptible to seizures, whereas the loss of neuronal GPR35 lowered the seizure threshold. These findings indicate that GPR35 signaling operates primarily through microglia in epilepsy.

### 2.5 PDGFA is a Downstream Target of GPR35 in Hippocampal Tissue to Regulate 4wKA‐Induced Epileptic Seizures

**FIGURE 5:**
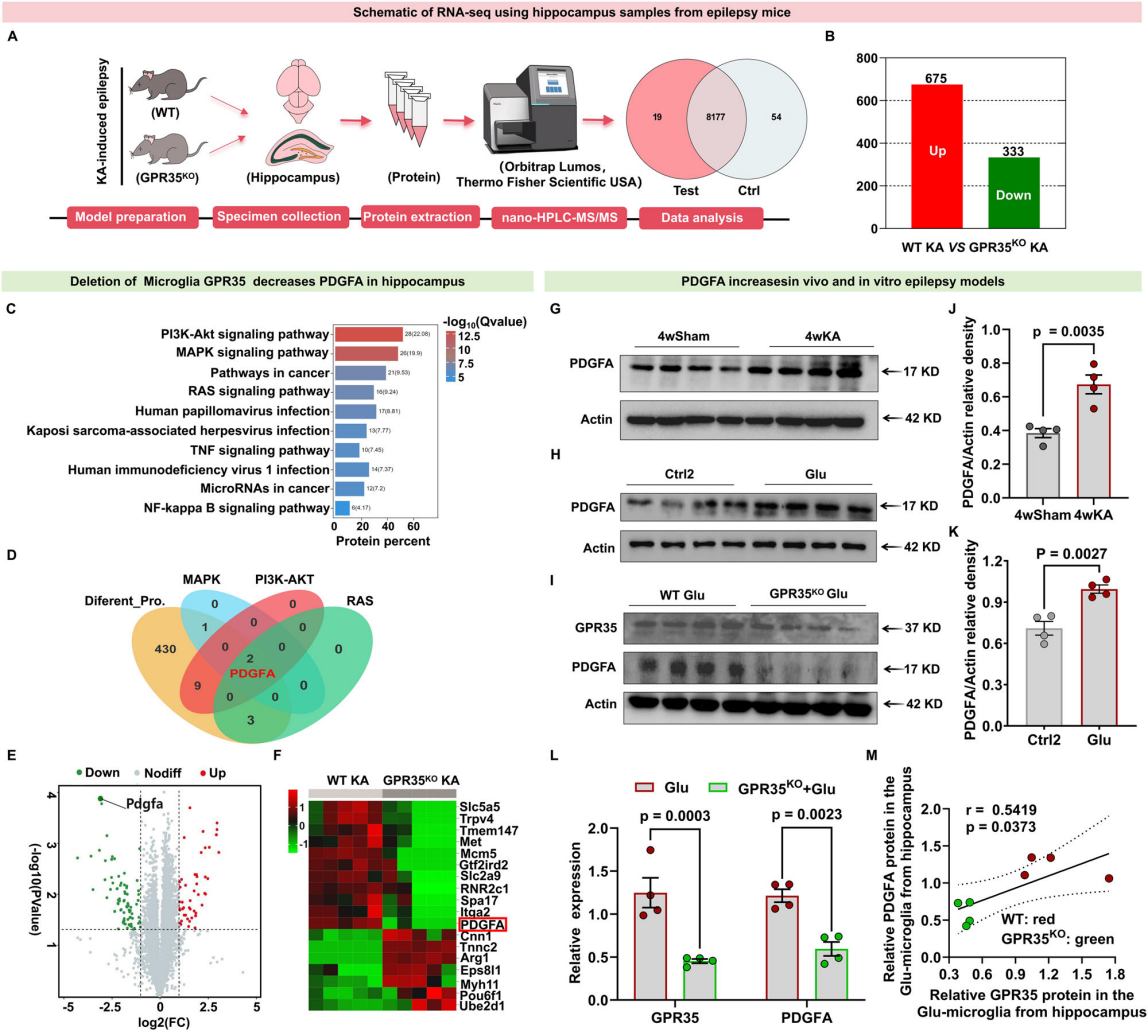
Platelet‐derived growth factor A (PDGFA) is a downstream target of GPR35 in the kainic acid (KA)‐induced in vivo model, and the Glu‐induced in vitro epileptic model. (A) Schematic of RNA‐seq using hippocampal tissue samples from GPR35^KO^ KA mice (n  =  5) and WT KA mice (n  =  5) at the 4‐week time point. (B) Differential protein analysis. A total of 675 proteins were upregulated, and 333 were downregulated expression in KA‐induced GPR35^KO^ mice compared with wild‐type (WT) mice (n = 5). (C) Transcriptome sequencing identified the top 10 enriched epilepsy‐related inflammatory pathways. (D) Venn diagram showing the intersection of the three most significantly enriched pathways (PI3K‐AKT, MAPK, and RAS), identifying PDGFA as a common gene. (E) Volcano plot of total proteins from the hippocampal tissues of WT and GPR35^KO^ mice treated with KA for 4 weeks. (F) Hierarchical clustering heatmap of total proteins, highlighting the most significantly upregulated (red) and downregulated (green) proteins. (G,J) Representative Western blot (G) and quantification (J) of PDGFA protein levels in mice at 4 weeks following KA‐induced epilepsy or sham (n = 4). Two‐tailed unpaired *t* test was used for analysis. (H,K) Representative Western blot (H) and quantification (K) of PDGFA protein levels in Glu‐treated and control primary microglia from the hippocampal tissue of WT mice. (n = 4). Two‐tailed unpaired *t* test was used for analysis. (I,L.) Representative Western blot (I) and quantification (L) of GPR35 and PDGFA protein levels in Glu‐treated primary microglia from GPR35^KO^ mice compared with controls (n = 4). Two‐tailed unpaired *t* test was used for analysis. (M) Partial correlation analysis of PDGFA protein and GPR35 protein expression in the Glu‐treated primary microglia from GPR35^KO^ mice compared with controls at 4 weeks (n = 4). Pearson's correlation coefficient was used for analysis. Data are presented as mean ±standard error of the mean (SEM).

To elucidate the mechanisms underlying hippocampal GPR35‐mediated modulation of epilepsy, we performed quantitative proteomic analysis of hippocampal tissues from KA‐induced epileptic GPR35^KO^ and WT mice (Figure 5A). Our proteomic profiling identified 1508 quantifiable proteins. Significant differential expression was defined as a fold‐change ≥1.5 (*p* < 0.05) and a false discovery rate (FDR) < 0.05 across multiple samples. In total, 675 proteins were upregulated, and 333 proteins were downregulated expression in GPR35^KO^ epileptic mice compared with WT epileptic mice (Figure 5B). The KEGG analysis revealed several epilepsy‐associated inflammatory pathways, with the PI3K‐AKT pathway showing the most significant enrichment (Figure 5C). The top three enriched pathways were PI3K‐AKT, MAPK, and RAS. PDGFA was identified as the common intersecting gene among these pathways using a venn diagram (Figure 5D). PDGFA is a potent mitogenic factor originally identified in platelets. Within the central nervous system, PDGFA is predominantly expressed in glial cells. Although microglia show minimal basal PDGFA expression, Activation significantly upregulates PDGFA production [23]. Notably, PDGFA‐based drug delivery systems with high CNS‐targeting specificity have been developed and show therapeutic potential for neuroinflammatory disorders [24]. Further analysis revealed significantly reduced PDGFA expression in GPR35^KO^ epileptic mice compared with WT epileptic controls (Figure 5E,F)

To validate these findings, we used both in vivo and in vitro models. In KA‐induced epileptic mice, PDGFA expression was significantly upregulated compared with sham controls (Figure 5G,J; Table S29), consistent with observations in the in vitro epilepsy model (Figure 5H,K; Table S30). Furthermore, we assessed the protein expression of GPR35 and PDGFA via western blot in Glu‐induced primary microglia isolated from WT and GPR35^KO^ mice. The results revealed a marked reduction in both GPR35 and PDGFA protein levels in GPR35^KO^ microglia compared with the WT controls (Figure 5I,L; Table S31), supporting the role of GPR35 as a potential positive regulator of PDGFA expression. Further correlation analysis indicated a strong positive relationship between GPR35 and PDGFA protein levels in Glu‐induced primary microglia (Figure 5M; Table S32). To further investigate the expression of PDGFA in other cell types, western blot analysis was performed on KA‐induced epileptic models of *GPR35^f/f^*, *CaMKIIα‐Cre;GPR35^f/f^*, and *CX3CR1‐Cre;GPR35^f/f^* Mice. Compared with *GPR35^f/f^* epileptic controls, PDGFA expression was significantly downregulated in *CX3CR1‐Cre;GPR35^f/f^* epileptic mice, whereas no statistically significant difference was observed in *CaMKIIα‐Cre;GPR35^f/f^* Mice (Figure S8A,B). Consistent with this, a concurrent decrease in both GPR35 and PDGFA protein levels was observed in Glu‐induced *CX3CR1‐Cre;GPR35^f/f^* microglia compared with *GPR35^f/f^* control mice (Figure S8C,D). These findings support a model in which GPR35 acts as a positive regulator of PDGFA expression, primarily through a microglia‐dependent mechanism in epilepsy. Collectively, our results suggest that PDGFA may mediate the anti‐epileptic effect of GPR35 by modulating inflammatory responses, thereby reducing susceptibility to epilepsy

### 2.6 GPR35 Represses PDGFA Degradation via the Ubiquitin–Proteasome Pathway

**FIGURE 6:**
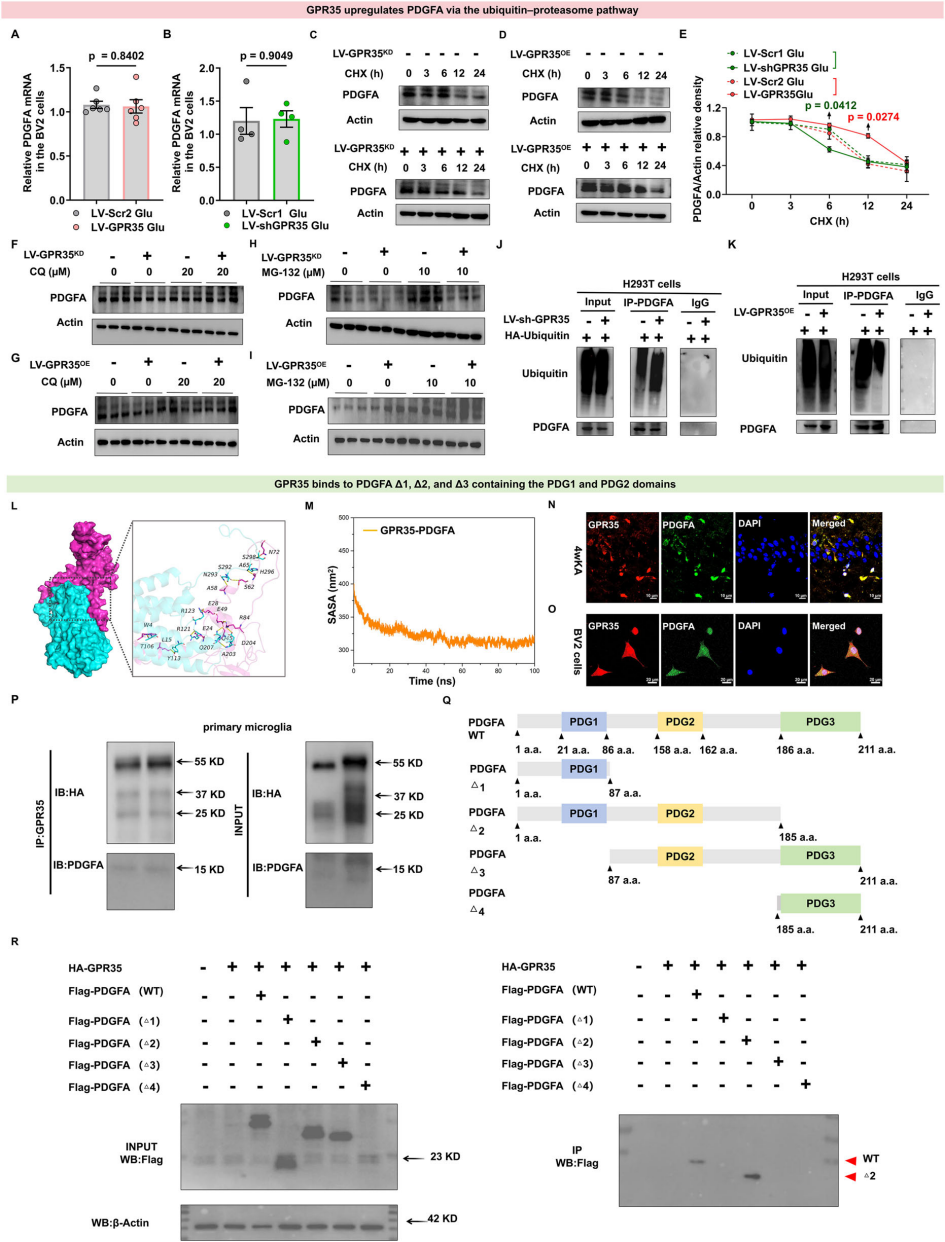
GPR35 binding to platelet‐derived growth factor A (PDGFA)– platelet‐derived growth factor 2 (PDGF2) represses PDGFA degradation via the ubiquitin–proteasome pathway. (A,B) Quantitative real‐time PCR (qRT‐PCR) analyses of PDGFA mRNA levels in BV2 cells with transfected with LV‐shGPR35 or LV‐GPR35 lentivirus (n = 4). Two‐tailed unpaired *t* test was used for analysis. Data are presented as mean ± standard error of the mean (SEM). (C–E) Western blot and quantification of PDGFA protein stability using CHX (200 µg mL^−1^) in GPR35 knockeddown (C and E) or GPR35‐overexpressing cells (D and E). One‐way ANCOVA was used for analysis. Data are presented as mean±SEM. (F,G) Western blot analysis of PDGFA in cells transfected with LV‐shGPR35 or LV‐GPR35 in the presence of the autophagy/lysosome inhibitor chloroquine (CQ, 20 µm, n = 6). One‐way ANCOVA was used for analysis. Data represent mean ± SEM. (H,I) Glutamate‐stimulated BV2 cells transfected with LV‐shGPR35 or LV‐GPR35 overexpression lentivirus, treated with MG132 (10 µm, proteasome inhibitor). PDGFA detected by Western blot (n = 4). One‐way ANCOVA was used for analysis. Data are presented as mean±SEM. (J,K) Western blot analysis of PDGFA ubiquitination in HEK293T cells with GPR35 knockdown (J) or overexpression of GPR35 (K) IgG was used as a negative control. (L) Surface‐rendered PDGFA and GPR35 protein structures. (M) Detailed binding mode of PDGFA with GPR35. Blake dashed lines represent hydrogen bonds or salt bridges. (N) Immunocytochemical analysis showing GPR35 and PDGFA localization in mouse hippocampal tissue (n = 3). Scale bars = 10 µm. (O) Immunocytochemical analysis showing GPR35 and PDGFA localization in BV2 cells (n = 3). Scale bars = 20 µm. (P) Primary microglia transfected with HA‐GPR35 and HA‐PDGFA subjected to anti‐HA immunoprecipitation. (Q) Schematic diagram of PDGFA deletion mutants PDGFA Δ1 contains PDG1; PDGFA Δ2 contains the PDG1 + PDG2; PDGFA Δ3 contains the PDG2 + PDG3; PDGFA Δ4 contains PDG3 (Δ1:1 a.a.–86 a.a.; Δ2:1 a.a.–185 a.a.; Δ3: 87 a.a.–211 a.a.; Δ4: 185 a.a.–211 a.a.). (R) HEK293T cells were co‐transfected with GPR35 and each Flag‐PDGFA deletion mutants, followed by anti‐Flag immunoprecipitation.

To investigate the regulatory mechanism by which GPR35 controls PDGFA expression, PDGFA mRNA levels were quantified. First, we selected and verified the lentivirus (Figure S9). We found that GPR35 deficiency did not alter PDGFA transcript levels (Figure 6A,B; Tables S33 and S34), suggesting that GPR35 may regulate PDGFA protein stability. Glu‐induced BV2 cells were treated with the protein synthesis inhibitor cycloheximide (CHX; 200 µg/mL) for 3, 6, 12, and 24 h. Vehicle‐treated cells served as controls. After treatment, cells were harvested, lysed, and PDGFA protein expression was analyzed by Western blot. The half‐life of PDGFA was significantly shortened following GPR35 knockdown (GPR35^KD^) in BV2 cells (Figure 6C,E; Tables S35 and S36). Conversely, treatment with CHX led to a longer PDGFA protein in GPR35‐overexpression (GPR35^OE^) cells (Figure 6D,E). We next investigated the molecular mechanism by which GPR35 regulates PDGFA expression in Glu‐induced primary microglia. We evaluated two major protein degradation pathways: proteasomal and autophagic degradation pathways [25]. The autophagy inhibitor chloroquine (CQ, 20 µm) had no effect on GPR35‐regulated PDGFA expression (Figure 6F,G; Figure S10A,B). In contrast, treatment with the 26S proteasome inhibitor MG‐132 (10 µm) in primary microglia blocked the enhanced PDGFA degradation caused by GPR35^KD^ (Figure 6H; Figure S10C). Moreover, MG‐132 further increased PDGFA expression in GPR35^OE^ cells (Figure 6I; Figure S10D).

Based on these results, we conclude that the GPR35 stabilizes PDGFA via the ubiquitin‐proteasome pathway. After endogenous PDGFA was immunoprecipitated in cells transfected with HA‐tagged ubiquitin, markedly increased PDGFA ubiquitination was detected in GPR35^KD^ HEK293T cells (Figure 6J). Consistently, PDGFA ubiquitination was reduced in the GPR35^OE^ HEK293T cells compared with that in the control cells (Figure 6K). These results indicate that GPR35 stabilizes PDGFA expression by inhibiting its ubiquitin–proteasome‐mediated degradation.

### 2.7 GPR35 Exerts its Biological Functions by Binding to the PDGF Domain 2 (PDG2)

To delineate the molecular mechanisms underlying GPR35‐mediated modulation of PDGFA modulation, we systematically screened and validated direct physical interactions between GPR35 and PDGFA‐related signaling components. We characterized the protein–protein interactions (PPIs) between GPR35 and PDGFA through integrated structural and functional analyses. Using 100‐ns molecular dynamics (MD) simulations performed in GROMACS 2020 with the AMBER99SB‐ILDN force field in TIP3P explicit solvent, we identified the structural basis of the GPR35–PDGFA interaction. Critical binding interfaces included PDGFA residues ASN‐72, ALA‐65, and ARG‐84 and GPR35 residues SER‐298, HIS‐296, and ASP‐204 (Figure 6L). These residues form a network of intermolecular interactions, including persistent hydrogen bonds, stable salt bridges, and hydrophobic contacts, that collectively stabilize the complex (Figure 6M; Figure S11, Tables S4 and S5).

Confocal immunofluorescence co‐localization analyses revealed compartment‐specific spatial proximity between GPR35 and PDGFA in hippocampal tissue from KA‐induced epileptic mice (Figure 6N) and Glu‐induced BV2 cells (Figure 6O). Co‐immunoprecipitation (Co‐IP) in primary microglia confirmed direct molecular binding, with enrichment above IgG controls (Figure 6P). To assess whether this mechanism extends to other cell types, Co‐IP performed in the murine HT22 cell line also detected GPR35‐PDGFA interactions (Figure S12). Together, these data demonstrate a specific in vivo and in vitro interaction between GPR35 and PDGFA, which is not restricted to microglial cells.

To identify the specific PDGFA domain responsible for binding GPR35, we generated deletion mutants of PDGFA (Figure 6Q; Table S6). Each deletion form of FLAG‐tagged deletion construct was transfected into HEK293T cells. Co‐IP analysis revealed that GPR35 binds to PDGFA Δ2 containing the PDG2 domains, but the other deletion forms containing the PDG2, PDG1, and PDG3 domains were not detected (Figure 6R). These findings mechanistically confirm that GPR35 directly engages PDGFA through its PDG2 domain, thereby activating PDGFA‐mediated downstream signaling. Collectively, our results provide compelling evidence that PDGFA exhibits strong binding affinity for GPR35, forming a stable protein complex capable of mediating GPR35‐dependent biological functions in the nervous system.

### 2.8 Deletion of Microglia‐Specific GPR35 Exacerbates Epileptic Seizures and Neuroinflammation via PI3K‐AKT Signaling

**FIGURE 7:**
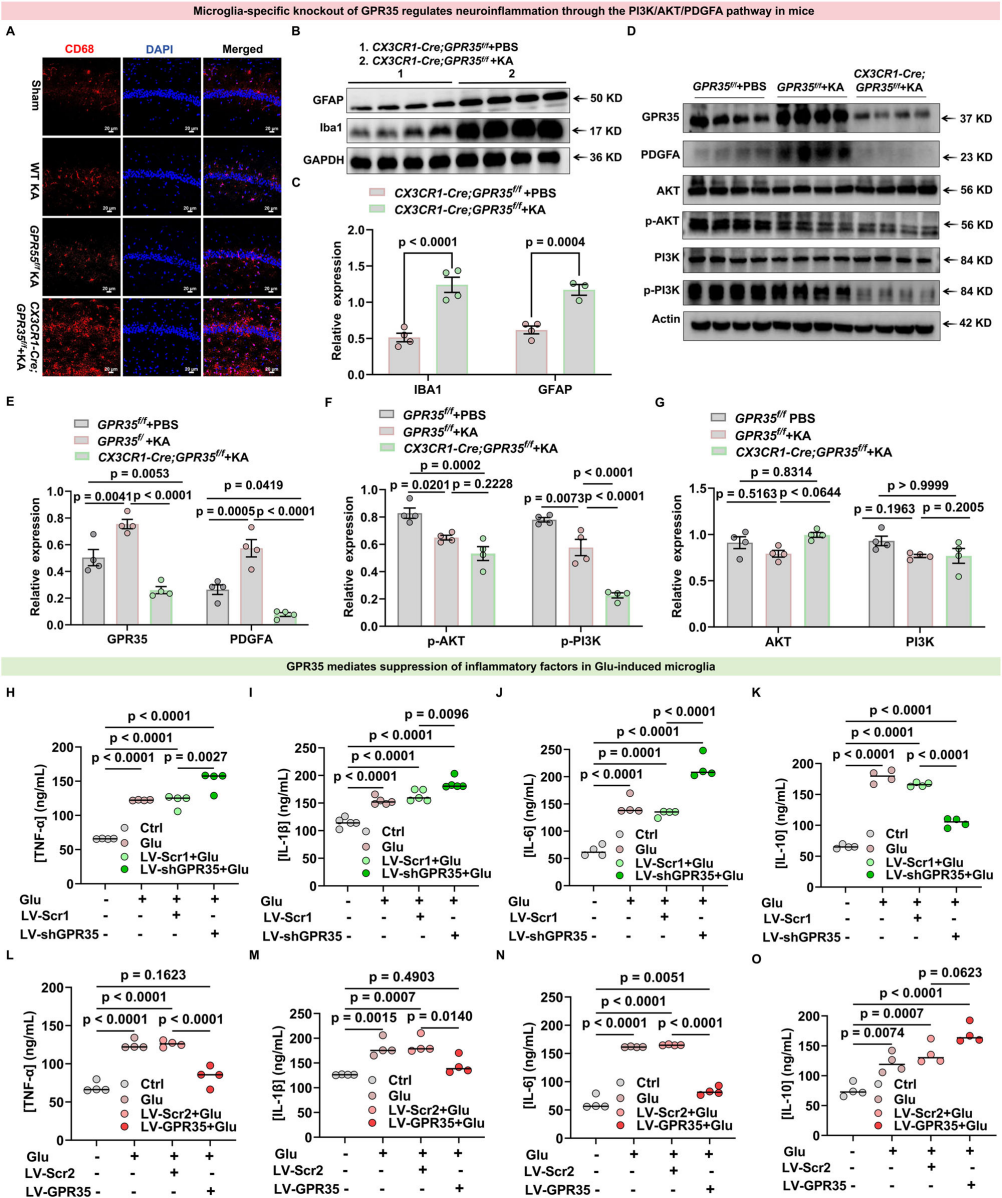
Microglia‐specific knockout of GPR35 regulates epileptogenesis and neuroinflammation in mice through the phosphoinositide 3‐kinase (PI3K)‐AKT‐platelet‐derived growth factor A (PDGFA) pathway. (A) Representative images of immunofluorescence images showing CD68 expression in hippocampal sections from 4wsham, 4wKA, 4w*GPR55^f/f^* KA, and 4w*CX3CR1‐Cre;GPR35^f/f^* KA groups (n = 3). Scale bar: 20 µm. (B,C) Representative Western blot (B) and quantification (C) of IBA1 and glial fibrillary acidic protein in hippocampus from KA‐induced *CX3CR1‐Cre;GPR35^f/f^* and KA‐induced *GPR35^f/f^* mice (n = 4). One‐way ANCOVA was used for analysis. (D–G) Representative Western blot (D) and quantification (E–G) of GPR35, PDGFA, p‐AKT, and p‐PI3K in the hippocampus from KA‐induced *CX3CR1‐Cre;GPR35^f/f^* and KA‐induced *GPR35^f/f^* mice (n = 4). Two‐way ANCOVA was used for analysis. (H–K) Cytokines levels (tumor necrosis factor [TNF]‐α, interleukin [IL]‐1β, IL‐6, IL‐10) in Glu‐BV2 cells treated with LV‐shRNA‐GPR35 or control (n = 4). One‐way ANCOVA was used for analysis. (L–O) Cytokines levels (TNF‐α, IL‐1β, IL‐6, IL‐10) in Glu‐BV2 cells treated with LV‐GPR35 overexpression or control (n = 4). One‐way ANCOVA was used for analysis. Data are presented as mean ± standard error of the mean (SEM).

To investigate brain inflammation induced by GPR35 in an epileptic mouse model, we first confirmed that Cre expression did not introduce confounding effects, as *CX3CR1‐Cre* mice exhibited comparable systemic GPR35 levels and basal inflammatory protein expression relative to *GPR35^f/f^* littermates (Figure S13). With Cre effects excluded, we examined the number of microglial lysosomal marker CD68‐positive cells. CD68^+^ cells were increased in the hippocampus of *CX3CR1‐Cre;GPR35^f/f^* mice following KA injection, as shown by immunofluorescence, indicating increased phagocytotic activity (Figure 7A). Western blot analysis of brain samples at 4 weeks post‐KA revealed that *CX3CR1‐Cre;GPR35^f/f^* mice displayed elevated microglial marker IBA1 and astrocyte marker glial fibrillary acidic protein expression, particularly IBA1, compared with *GPR35^f/f^* mice (Figure 7B,C; Table S37). Based on our initial findings, Kyoto Encyclopedia of Genes and Genomes (KEGG) pathway analysis identified the PI3K–AKT signaling pathway as the most significantly enriched inflammatory pathway in epilepsy. Subsequent bioinformatic analysis to identify potential downstream substrates of PDGFA implicated PI3K and AKT as key effectors. Therefore, we measured PI3K and AKT phosphorylation. the primary effector molecules in the pathway, and found that in hippocampal brain tissue of *GPR35^f/f^* treated with KA for 4 weeks, phosphorylation of PI3K at Ser467, 199 and AKT at Ser473 was dramatically increased, whereas its expression was decreased in the KA‐induced *CX3CR1‐Cre;GPR35^f/f^* mice, compared with the control. In hippocampal tissue from *GPR35^f/f^* mice treated with KA for 4 weeks, phosphorylation of PI3K at Ser467/Ser199 and AKT at Ser473 was markedly increased, whereas these phosphorylation levels were reduced in KA‐induced *CX3CR1‐Cre;GPR35^f/f^* mice. Furthermore, in KA‐induced *GPR35^f/f^* mice and KA‐induced *CX3CR1‐Cre;GPR35^f/f^* mice, decreased p‐Ser467/Ser199‐PI3K and p‐Ser473‐AKT levels were accompanied by reduced GPR35 and PDGFA protein expression (Figure 7D–G; Tables S38–S40). Importantly, we confirmed a positive correlation among GPR35, PDGFA, p‐Ser467/Ser199‐PI3K, and p‐Ser473‐AKT levels. These data suggest that microglial GPR35 deficiency causes aberrant PDGFA expression, which leads to reduced phosphorylation of PI3K (Ser467/Ser199) and AKT (Ser473), thereby inhibiting anti‐inflammatory cytokine release and promoting pro‐inflammatory cytokine production. This dysregulation exacerbates inflammation and facilitates epileptogenesis.​

We further validated the regulatory role of GPR35 in inflammatory responses using a primary microglial model with glutamatergic stimulation. Enzyme‐linked immunosorbent assay (ELISA) revealed that GPR35 knockdown via shRNA significantly increased secretion of TNF‐α, IL‐1β, and IL‐6 (Figure 7H–J; Tables S41–S43), while markedly decreasing IL‐10 secretion under glutamatergic stimulation (Figure 7K; Table S44). Conversely, GPR35 overexpression significantly reduced TNF‐α, IL‐1β, and IL‐6 secretion and increased IL‐10 secretion following glutamatergic excitation (Figure 7L–O; Tables S45–S48). These results indicate that microglial GPR35 activation bidirectionally modulates key inflammatory cytokines under epileptiform conditions.

### 2.9 Microglia‐Specific Overexpression of PDGFA Activates PDGFA–PI3K–AKT Cascade in the Hippocampus

GPR35 transduces signals by structurally coupling with distinct ligand receptor complexes, triggering downstream signaling cascades. In this study, we report for the first time that PDGFA plays a predominant role in GPR35‐mediated inflammation‐related pathways. Based on RNA‐seq and validation data demonstrating a positive correlation between PDGFA and GPR35 expression, we examined PDGFA as a potential mediator of GPR35‐dependent regulation of epilepsy. Given that PDGFA expression is elevated under epileptic conditions, we investigated whether PDGFA deficiency alters the functional consequences of GPR35 activity.

**FIGURE 8:**
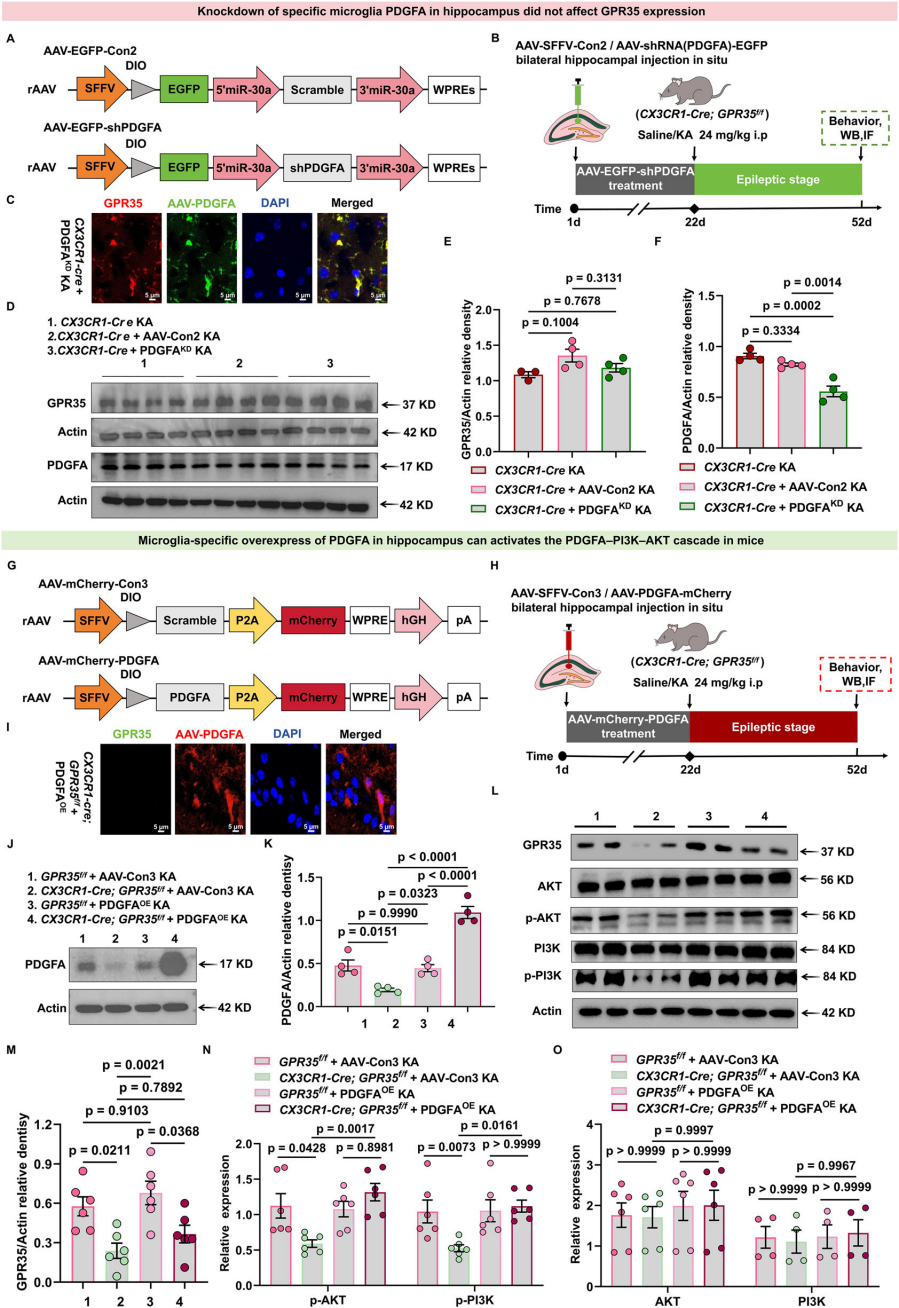
Inhibition of platelet‐derived growth factor A (PDGFA) in the microglia suppresses the PDGFA–PI3K‐AKT cascade in the hippocampus. (A) Schematic representation of spleen focus‐forming virus (SFFV) promoter‐driven enhanced green fluorescent protein (EGFP; (AAV‐Con2) or SFFV promoter‐driven shPDGFA with EGFP (AAV‐shPDGFA). (B) Schematic diagram of the experimental procedure. (C) Immunocytochemical analysis of GPR35 and AAV‐EGFP‐PDGFA localization in *CX3CR1‐Cre* mouse brain tissue infected with infection with AAV‐EGFP‐Con or AAV‐EGFP‐shPDGFA (PDGFA^KD^) (n = 3). Scale bars = 5 µm. (D–F) Representative Western blot (D) and quantification (E and F) of hippocampal GPR35 and PDGFA protein levels in *CX3CR1‐Cre* mice infected with AAV‐EGFP‐Con or AAV‐EGFP‐shPDGFA at 4‐week time points following KA‐induced or sham treatment (n = 4). One‐way ANCOVA was used for analysis. (G) Schematic representation of SFFV promoter‐driven mCherry (AAV‐mCherry‐Con3) or SFFV promoterdriven PDGFA with mCherry (AAV‐mCherry‐PDGFA). (H) Schematic diagram of the experimental procedure. (I) Immunocytochemical analysis of GPR35 and AAV‐mCherry‐PDGFA localization in *CX3CR1‐Cre;GPR35^f/f^* mouse brain tissue infected with AAV‐mCherry‐PDGFA (PDGFA^OE^) (n = 3). Scale bars = 5 µm. (J,K) Representative Western blot (J) and quantification (K) of hippocampal PDGFA protein levels in *CX3CR1‐Cre;GPR35^f/f^* mice infected with AAV‐mCherry‐Con3 or AAV‐mCherry‐PDGFA at 4‐week time points following KA‐induced or sham treatment (n = 4). One‐way ANCOVA was used for analysis. (L–O) Representative Western blot of GPR35, AKT, PI3K, p‐AKT, and p‐PI3K (L) and quantification of hippocampal GPR35, AKT, PI3K, p‐AKT, p‐PI3K protein levels (M–O) in *CX3CR1‐Cre;GPR35^f/f^* mice infected with AAV‐mCherry‐Con3 or AAV‐mCherry‐PDGFA at 4‐week time points following KA‐induced seizure or sham treatment (n = 4). Two‐way ANCOVA was used for analysis. Data are presented as mean ± standard error of the mean (SEM).

To test this hypothesis, we employed an improved viral delivery system to selectively target PDGFA in bilateral hippocampal microglia [26, 27]. *CX3CR1‐Cre* mice were injected with either rAAV‐SFFV‐DIO‐EGFP (AAV‐Con2) or rAAV‐SFFV‐DIO‐shPDGFA‐EGFP (PDGFA^KD^) (Figure 8A; Table S7), thereby achieving Cre‐dependent, microglia‐specific PDGFA knockdown (Figure S14A,C). KA‐induced seizures were administered 21 days after viral infection, and mice were subsequently maintained for a total of 4 weeks (Figure 8B). Immunofluorescence confirmed markedly reduced PDGFA expression in KA‐induced *CX3CR1‐Cre* mice treated with AAV‐shPDGFA, whereas GPR35 expression remained detectable (Figure 8C). Compared with KA‐induced *CX3CR1‐Cre* mice receiving AAV‐Con2, PDGFA^KD^ mice exhibited reduced PDGFA protein levels without significant alteration in GPR35 protein expression (Figure 8D–F; Tables S49 and S50). These data show that GPR35 expression is not affected by PDGFA modulation, confirming that GPR35 functions upstream of PDGFA.

To restore microglia‐specific PDGFA expression, the same viral delivery system was used to perform bilateral hippocampal stereotaxic injections of either rAAV‐SFFV‐DIO‐mCherry (AAV‐Con3) or rAAV‐SFFV‐DIO‐PDGFA‐mCherry (PDGFA^OE^) into *CX3CR1‐Cre;GPR35^f/f^* mice and control mice (Figure 8G). Expression was confirmed 21 days after viral injection (Supplementary Figure S14B,D), and mice were subsequently maintained for 4 weeks following KA administration (Figure 8H).​Immunofluorescence showed increased PDGFA expression in KA‐induced *CX3CR1‐Cre;GPR35^f/f^* mice receiving AAV‐PDGFA, whereas GPR35 expression remained significantly reduced (Figure 8I). Western blot analysis confirmed elevated PDGFA protein levels in KA‐induced* CX3CR1‐Cre;GPR35^f/f^* mice treated with AAV‐PDGFA compared with KA‐induced controls (Figure 8J,K; Table S51).​We next examined key effector molecules in the PI3K and AKT pathways. AAV‐PDGFA^OE^‐*CX3CR1‐Cre;GPR35^f/f^* mice exhibited significantly increased phosphorylation of PI3K at Ser467/Ser199 and AKT at Ser473 in hippocampal tissues, whereas phosphorylation levels were reduced in AAV‐Con3‐*CX3CR1‐Cre;GPR35^f/f^* mice compared with controls. Moreover, reduced p‐Ser467/Ser199‐PI3K and p‐Ser473‐AKT levels were associated with decreased GPR35 protein expression in *CX3CR1‐Cre;GPR35^f/f^* mice (Figure 8L–O; Tables S52–S54). These findings indicate that GPR35 activates the PDGFA‐PI3K‐AKT signaling cascade in microglia to exert its biological effects.

### 2.10 L‐Kyna, an Agonist of GPR35 Reduces Epileptogenesis and Epilepsy‐Related Cognitive Impairment in Mice

**FIGURE 9:**
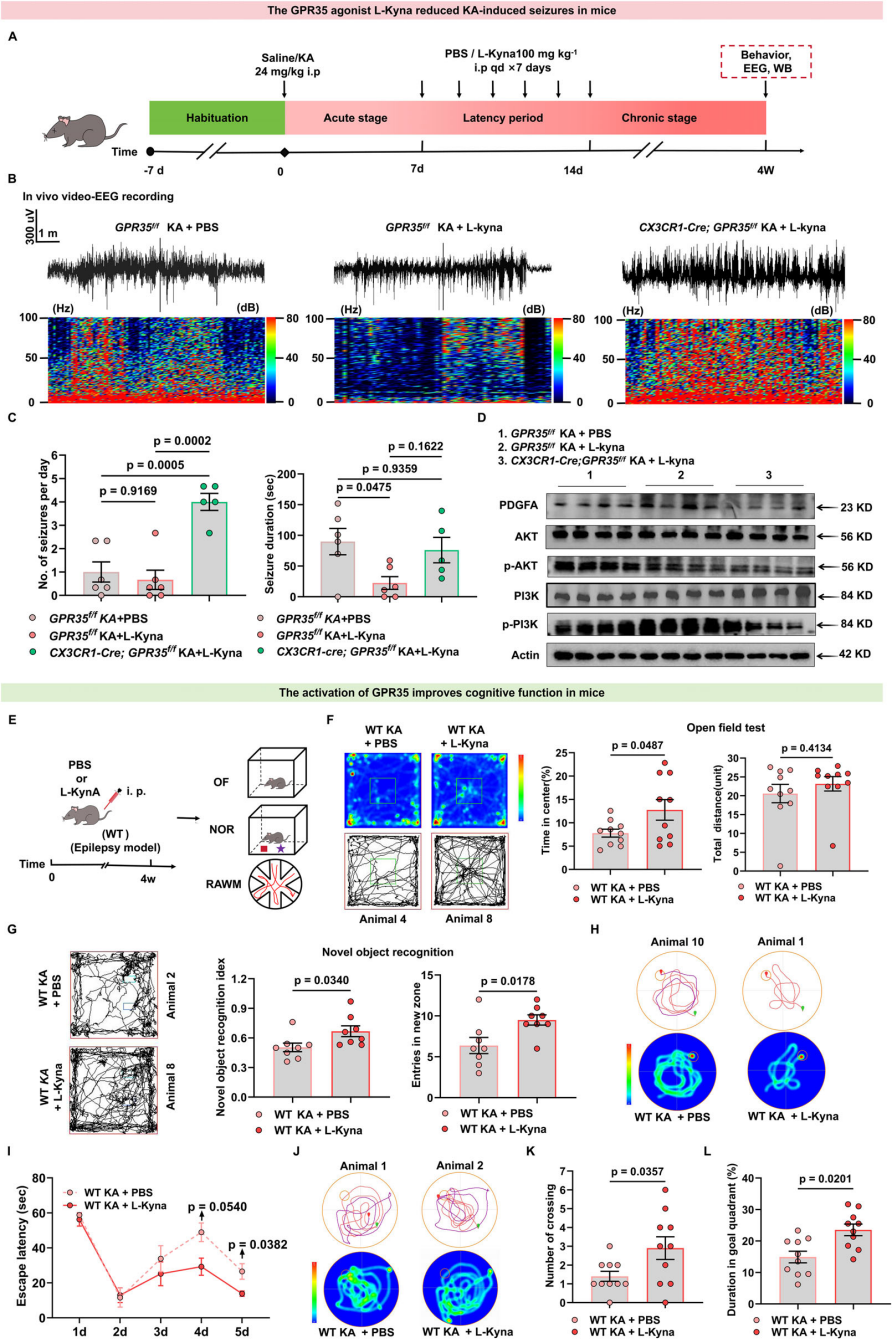
The GPR35 agonist L‐kynurenine (L‐Kyna) alleviates kainic acid (KA)‐induced epileptic seizures and epilepsy‐related cognitive deficits in mice. (A) Schematic of the administration of treatments to epileptic mice and the testing timeline. (B,C) Electrographic seizure‐like activities assessed at the chronic stage by in vivo EEG (*GPR35^f/f^* KA + PBS, n  =  6; *GPR35^f/f^* KA + L‐Kyna, n  =  6; *CX3CR1‐Cre;GPR35^f/f^* KA + L‐Kyna, n  =  5). One‐way ANCOVA was used for analysis. (D) Representative Western blot of platelet‐derived growth factor A (PDGFA), AKT, PI3K, p‐AKT, p‐PI3K protein levels in *GPR35^f/f^* or *CX3CR1‐Cre;GPR35^f/f^* mice treated with phosphate‐buffered saline (PBS) or L‐Kyna (100 mg kg^−1^ i.p., once daily for 7 days) at 4‐week time points following KA‐induced seizure or sham treatment (n = 4). (E) Schematic of the administration of treatments to epileptic mice and the cognitive testing timeline. (F) (Left) Representative traces of wild‐type (WT) KA mice treated with PBS or L‐Kyna (100 mg kg^−1^, i.p., once daily for 7 days during seizure latency) in the open‐field arena at 4 weeks. The square area indicates the center zone. (Right) Quantification of total distance traveled and time spent in the center (n = 10). Two‐tailed unpaired *t* test was used for analysis. Data are represented as mean ± standard error of the mean (SEM). (G) (Left) Representative traces of WT KA + PBS and WT KA + L‐Kyna mice in the NOR test for 4 weeks. The blue rectangle indicates the novel object zone. (Right) Quantification of the percentage of time exploring the novel object (n = 10). Two‐tailed unpaired *t* test was used for analysis. (H–L) Spatial learning and memory assessed using the Morris water maze in PBS‐treated or L‐Kyna‐treated KA‐induced WT mice. (H,I) Representative swim paths and heat map (H), and latency to find the hidden platform (I) (n  =  10). Two‐way ANCOVA was used for analysis. (J–L) Representative track plot of the probe test (J), quantification of numbers of platform crossings (K), and time spent in target quadrant (L). Two‐tailed unpaired *t* test was used for analysis. Data are presented as mean ± SEM.

KYNA, an endogenous agonist of the orphan receptor GPR35 [9], demonstrates significant clinical relevance in epilepsy. Animal models of epileptic spasms exhibit substantially reduced KYNA levels in brain tissue. Consistently, diminished KYNA concentrations have been documented in cerebrospinal fluid and serum samples from patients with epilepsy [28]. Notably, its precursor L‐kynurenine (L‐Kyna) readily crosses the blood‐brain barrier [29]. We investigated whether L‐Kyna supplementation could suppress KA‐induced epileptogenesis. *GPR35^f/f^* mice and *CX3CR1‐Cre;GPR35^f/f^* mice were administered KA to induce epileptogenesis. Following KA administration, the mice were maintained for 1 week post‐induction to establish baseline seizure latency. At this point, we initiated a 7‐day regimen of daily intraperitoneal injections of L‐Kyna (100 mg kg^−1^) or phosphate‐buffered saline (PBS) vehicle was then initiated in C57BL/6J WT mice. Mice were then continuously monitored and maintained for up to 4 weeks post‐KA to assess epileptogenesis (Figure 9A). During the chronic phase, L‐Kyna clearly reduced seizure duration; however, the frequency of spontaneous recurrent seizures between Days 29–31 remained unchanged (Figure 9B,C; Table S55), indicating that L‐Kyna alleviated the severity of KA‐induced chronic seizures. Western blot analysis revealed that KA‐induced *GPR35^f/f^* mice treated with L‐Kyna exhibited elevated PDGFA protein levels and significantly increased phosphorylation of PI3K (p‐Ser467/Ser199) and AKT (p‐Ser473) in hippocampal tissue compared with KA‐induced controls, whereas these effects were absent in L‐Kyna‐induced *CX3CR1‐Cre;GPR35^f/f^* mice (Figure 9D; Figure S15A–C). Furthermore, immunofluorescence quantification demonstrated a marked decrease in CD68‐positive microglia in L‐Kyna‐treated *GPR35^f/f^* mice, indicating reduced microglial phagocytic activity (Figure S15D). These data demonstrate that L‐Kyna ameliorates seizure severity through GPR35‐dependent activation of the PDGFA‐PI3K‐AKT pathway and modulation of microglial activity. Subsequently, we investigated the effect of L‐Kyna in the Glu‐induced cells model. By examining the dose‐dependent effects of different concentrations of L‐Kyna (12.5, 25, and 50 µm) on PDGFA and inflammatory cytokine mRNA expression in the in vitro model, we found that PDGFA mRNA expression in BV2 cells peaked following treated with 25 µm L‐Kyna (Figure S16A). Moreover, compared with the vehicle group, BV2 cells treated with 25 µm L‐Kyna exhibited a marked downregulation of pro‐inflammatory cytokines, including IL‐6, IL‐1β, and TNF‐α (Figure S16B–E). Consequently, 25 µm L‐Kyna was selected for subsequent experiments. Western blot analysis confirmed a significant increase in PDGFA protein levels in Glu‐induced primary microglia after L‐Kyna treatment compared with controls (Figure S16G). These findings indicate an effect of elevated L‐Kyna levels in the Glu‐induced in vitro model.

In addition, we evaluated whether GPR35 activation could improve epilepsy‐related cognitive function (Figure 9E). In the OFT, both groups traveled comparable distances. However, WT epileptic mice that were treated with L‐Kyna spent more time in the center of the arena, indicating reduced anxiety‐like behavior (Figure 9F; Table S56). During NOR testing, WT epileptic mice treated with L‐Kyna preferred the novel object over the familiar object, whereas PBS‐treated controls showed no preference (Figure 9G; Table S57). The Morris water maze was used to evaluate learning and spatial memory over 5 days of acquisition trials, followed by a day‐6 probe test. Compared with PBS‐treated WT epileptic mice, L‐Kyna‐treated WT epileptic mice exhibited shorter escape latencies, indicating improved learning ability (Figure 9H,I; Table S58). During the probe test, L‐Kyna‐treated epileptic mice crossed the former platform location more frequently and spent a higher percentage of time in the target quadrant (Figure 9J–L; Tables S59 and S60). These results demonstrate that L‐Kyna alleviates impaired spatial memory in epileptic mice. Together, these behavioral data indicate that L‐Kyna administration activates GPR35 and ameliorates cognitive function in epileptic mice.

Remarkably, *CX3CR1‐Cre;GPR35^f/f^* epileptic mice showed opposite results. We first evaluated cognitive functions in these mice (Figure S17A). *CX3CR1‐Cre;GPR35^f/f^* epileptic mice were reluctant to explore the central area and spent less time in the center than *GPR35^f/f^* epileptic mice, despite traveling comparable distances. Reduced center time indicated elevated anxiety‐ and depression‐like behavior (Figure S17B). Compared with control epileptic mice, *CX3CR1‐Cre;GPR35^f/f^* epileptic mice exhibited impaired preference for the novel object (NOR) and the novel arm (Y maze) (Figure S17C,D), indicating impaired spatial working memory. We then evaluated the effect of L‐Kyna on cognition in *CX3CR1‐Cre;GPR35^f/f^* epileptic mice (Figure S18A). In the OFT, L‐Kyna‐treated and PBS‐treated *CX3CR1‐Cre;GPR35^f/f^* epileptic mice showed comparable distances traveled and time in the center, indicating no improvement in anxiety‐like behavior (Figure S18B). During the NOR and Y maze tests, neither group showed preference (Figure S18C,D). In the Morris water maze test, L‐Kyna‐treated *GPR35^f/f^* epileptic mice, crossed the former platform location more frequently and spent significantly more time in the target (Figure S18E,F). These results demonstrate that L‐Kyna did not alleviate the impaired spatial memory in *CX3CR1‐Cre;GPR35^f/f^* epileptic mice. Together, these behavioral data indicate that the L‐Kyna administration does not restore cognitive function in *CX3CR1‐Cre;GPR35^f/f^* epileptic mice. Collectively, these findings demonstrate that L‐Kyna mitigates epileptogenesis and improves epilepsy‐related cognitive deficits through GPR35 activation, effects that are absent in microglial GPR35‐deficient mice.

## 3 Discussion

**FIGURE 10:**
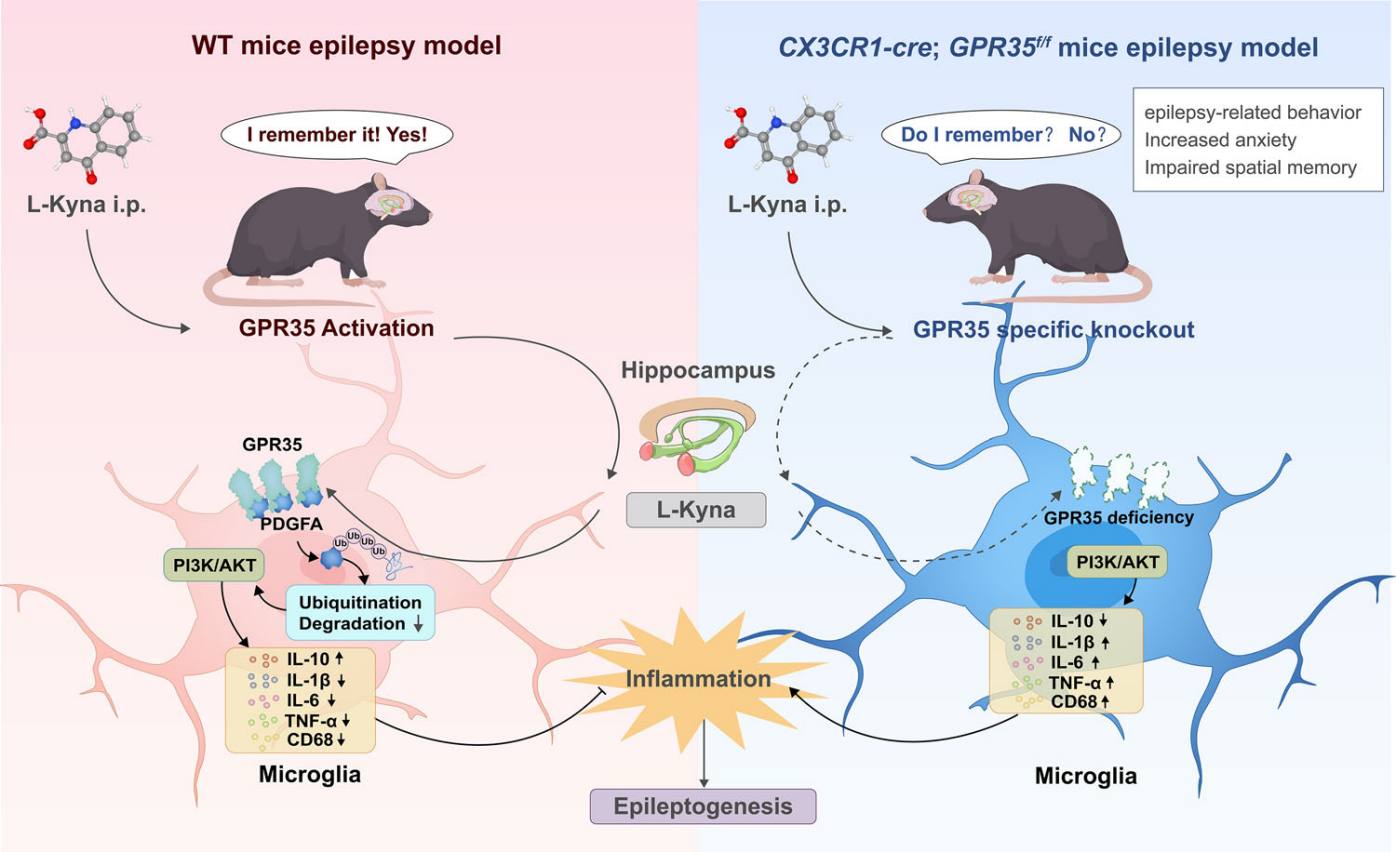
Schematic illustration of the microglial GPR35‐mediated neuroinflammatory cascade in the hippocampus and consequent progression of epileptogenesis. Activation of microglial GPR35 by its agonist L‐Kyna initiates a protective signaling cascade in the hippocampus. This pathway involves binding to PDGFA (via the PDG2 domain), activation of the PI3K‐AKT axis, suppression of pro‐inflammatory factor release, and promotion of anti‐inflammatory responses. Consequently, neuroinflammation is attenuated, leading to reduced epileptogenesis and improved cognitive deficits. GPR35 and its downstream effector PDGFA in hippocampal microglia are essential for modulating seizure susceptibility and associated cognitive impairments.

Our study is the first to elucidate the expression and function of GPR35 in epilepsy, demonstrating its neuroprotective role via the suppression of neuroinflammatory responses to mitigate epileptogenesis and associated cognitive comorbidities. We found that during epileptogenesis, microglia exhibited significant upregulation of GPR35 protein and mRNA. Global GPR35 knockout in C57BL/6 mice increased susceptibility to seizures and exacerbated cognitive comorbidities, whereas microglia‐specific GPR35 deletion increased seizure susceptibility, aggregated cognitive comorbidities, and elevated pro‐inflammatory cytokine expression. Conversely, the agonist L‐Kyna activated hippocampal microglial GPR35, which subsequently bound to PDGFA (PDG2), thereby activating the PI3K‐AKT signaling pathway. This activation inhibited the release of pro‐inflammatory factors and promoted the release of anti‐inflammatory factors, ultimately reducing neuroinflammatory responses and attenuating KA‐induced seizures and associated cognitive dysfunction. Critically, GPR35 and its downstream target PDGFA in hippocampal microglia are essential for epileptogenesis (Figure 10). Collectively, the GPR35‐PDGFA‐mediated neuroinflammatory cascade reveals an unexplored mechanism underlying the pathogenesis of epilepsy.

Previous studies by Kang et al. [30] and Miyamoto et al. [14] identified enriched GPR35 expression in brain tissue and cultured neurons, implicating its functional importance in neural processes. To evaluate GPR35 expression and function in vivo and in vitro, we used a post‐SE epilepsy model induced by KA or pilocarpine, a model involving repeated chemogenetic excitation of hippocampal neurons, and an in vitro glutamate‐stimulated model to demonstrate that GPR35 plays an important role in modulating epileptic activity. Critically, during the 4‐week chronic phase of epilepsy, GPR35 expression was significantly upregulated and functionally active within microglia, which was also observed in the in vitro model. This indicated that microglial GPR35 contributes to epileptogenesis. Collectively, these findings suggest that the observed increase in GPR35 levels likely represents a compensatory response to persistent epileptogenic inflammation aimed at counteracting disease progression.

To determine the role of GPR35 in epileptogenesis, we used two complementary strategies to suppress GPR35 expression. First, global GPR35 knockout mice were utilized to ablate GPR35 ubiquitously. However, the lack of cell type‐specificity inherent in this model prompted us to develop conditional knockout mice with microglia‐specific deletion (*CX3CR1‐Cre;GPR35^f/f^*) and hippocampal pyramidal neuron‐specific deletion (*CaMKIIα‐Cre;GPR35^f/f^*). This conditional strategy enables non‐invasive, and cell type‐specific deletion, providing superior spatial resolution than global knockout. Furthermore, the two conditional knockout models allowed direct assessment of GPR35's epileptogenic contributions in hippocampal pyramidal neurons vs. microglia, delineating cell type‐specific roles in disease pathogenesis. Both strategies exacerbated susceptibility to epilepsy, neuroinflammatory pathology, and cognitive comorbidities. Although microglia‐specific GPR35 knockout induced robust epileptic phenotypes, neuron‐specific deletion resulted in only mild seizure susceptibility. These findings establish GPR35 as a previously unrecognized protective factor and with cell type‐specific functional heterogeneity in epilepsy models. Notably, although *CX3CR1‐Cre* and *CaMKIIα‐Cre* mice are commonly used for cell type–specific studies, both can exhibit off‐target effects.Pre‐phenotypic assessments of *CaMKIIα‐Cre;GPR35^f/f^* and *CX3CR1‐Cre;GPR35^f/f^* mice revealed no abnormalities in morphology or other features. Cre‐only control experiments further confirmed that Cre expression alone does not affect systemic GPR35 expression or baseline inflammation, suggesting that the observed phenotypes primarily reflect GPR35 deletion rather than Cre artifacts. However, future studies using inducible *Cre^ERT2^* systems could provide additional validation of GPR35 function under physiological conditions.

Additionally, we demonstrated that microglial GPR35 orchestrates neuroinflammatory signaling in epilepsy. Mice with microglia‐specific GPR35 deletion exhibited exacerbated seizure severity upon KA exposure, aligning with the established role of neuroinflammation in hyperexcitability. Agudelo et al. [20] demonstrated that kynurenic acid activates GPR35 to promote anti‐inflammatory gene transcription. Miyamoto et al. [14] found that modulating KYNA‐mediated GPR35 signaling in CX3CR1^+^ macrophages markedly ameliorated experimental autoimmune encephalomyelitis. Our data demonstrated that seizure‐induced GPR35 activation drives anti‐inflammatory microglial polarization, whereas GPR35 ablation shifts microglia toward a pro‐inflammatory phenotype. Mechanistically, microglial GPR35 deletion significantly suppressed hippocampal IL‐10 (a validated anti‐epileptic cytokine) while elevating pro‐convulsive mediators (IL‐1β, IL‐6, TNF‐α), indicating that GPR35‐mediates neuroprotective reprogramming by enhancing enhances anti‐inflammatory signaling. Although this mechanism underlies GPR35's seizure‐suppressive effects, parallel pathways (e.g., impaired debris clearance) cannot be excluded. Importantly, emerging evidence counters the classical pro‐convulsive microglial viewpoint, demonstrating their protective roles [31, 32, 33]. Thus, targeted GPR35 modulation may represent a superior therapeutic alternative to global microglial suppression.

We identified PDGFA as a downstream target of GPR35. This ligand‐receptor interaction regulates microglial GPR35 function, as evidenced by coordinated upregulation of PDGFA and GPR35 in microglia during the 4‐week chronic phase of epilepsy. PDGFA expression was predominantly glial and decreased upon GPR35 knockdown, but increased following GPR35‐enhanced anti‐inflammatory activation. Conversely, PDGFA inhibition did not alter GPR35 expression, establishing PDGFA as a functional downstream effector of GPR35‐mediated microglial activation. We further demonstrated reduced hippocampal p‐PI3K and p‐AKT levels in epileptic mice, which were alleviated by pharmacological GPR35 agonism but remained unaltered in *CX3CR1‐Cre;GPR35^f/f^* mice. These findings indicate that microglial GPR35 activates the PI3K‐AKT pathway via PDGFA interactions to attenuate epileptogenesis. Thus, targeting GPR35 suppresses both neuroinflammation and PDGFA‐driven signaling. Future studies should incorporate EEG recordings to validate the effects of PDGFA conditional KO and to clarify the precise microglial functions of PDGFA.

We next investigated the mechanism by which microglial GPR35 suppresses epileptogenesis in hippocampal tissue. Our results demonstrated that the active N‐terminal peptide Ac87‐185 (corresponding to PDGFA structural domain 2) interacts with GPR35, elevates PDGFA protein levels, and enhances its phosphorylation state in microglia. Mechanistically, GPR35 binding to the PDGFA PDG2 domain impedes PDGFA degradation via the proteasomal pathway, thereby stabilizing PDGFA. Given that PDGFA activates the PI3K‐AKT pathway [34, 35], which is consistent with our KEGG analysis, we propose a novel signaling axis: GPR35 promotes PDGFA accumulation through PPIs, which subsequently activates PI3K‐AKT signaling to suppress neuroinflammation and attenuate seizure progression. Collectively, this study identified GPR35 and PDGFA as novel potential therapeutic targets against epilepsy and delineated the GPR35‐PDGFA‐PI3K/AKT signaling pathway as a previously unrecognized anti‐epileptogenic mechanism, providing new perspectives for targeted epilepsy therapeutics.

The high prevalence of neurobehavioral comorbidities in epilepsy is well established, severely compromising patient quality of life and highlighting the urgent need for effective therapies beyond currently limited options [36, 37]. Our neurobehavioral assessments demonstrated that global and microglia‐specific GPR35 deletion exacerbates anxiety/depression‐like behaviors and spatial memory deficits in epileptic mice, although OFTs revealed no changes in center time. This aligns with Cheng et al. [38] who demonstrated that a Gpr35‐modulated gut‐microbiota‐brain metabolic axis regulates depressive phenotypes. These findings contrast with reports that GPR35 knockout alleviates LPS‐induced depression (Kang et al.) [30] or Aβ_1__−__42_‐induced cognitive impairment (Liang et al.) [39], reflecting broader inconsistencies in behavioral studies influenced by experimental variables (age, sex, lighting). Similar contradictions exist for other targets: GLT‐1 deficiency reduces anxiety [40] whereas H_2_S‐induced GLT‐1 upregulation attenuates anxiety‐like behavior [41]; astrocytic ALKBH5 deletion produces antidepressant effects [42], whereas its overexpression mitigates LPS‐induced deficits [43]. Critically, L‐Kyna ameliorated cognitive dysfunction in control epileptic mice but failed to ameliorate deficits in microglia‐specific GPR35 knockouts, indicating cell‐type‐dependent duality. We propose that upregulation of microglial GPR35 serves as a defense mechanism aimed at restoring inflammatory homeostasis to maintain neuronal excitability after seizures. Its activation promotes the release of anti‐inflammatory mediators to suppress epileptogenesis, potentially overriding neuronal pro‐convulsive effects during chronic phases. This cellular specificity explains divergent outcomes across disease models, as exemplified by P2×7R antagonists exhibiting opposing convulsive effects in pilocarpine vs. KA models [44].

KYNA, a pharmacological agonist of GPR35and a tryptophan metabolite produced via the kynurenine pathway, exhibits antioxidant, neuroprotective, and anti‐inflammatory properties [45, 46]. Recent studies suggested that the kynurenine pathway may serve as a biomarker of neuroinflammation [47, 48, 49]. Dysregulation of the kynurenine pathway in epilepsy is linked to neuroinflammation; KYNA suppresses microglial activation and IL‐1β release, thereby reducing inflammation‐driven hyperexcitability in TLE [50] and has been proposed as a therapeutic strategy for infantile spasms [21, 51, 52]. However, the effects of GPR35 agonists in epilepsy remain unknown. Understanding how microglia respond to GPR35 agonists is crucial for optimizing targeted therapeutic strategies. In this study, we demonstrated that L‐Kyna, an endogenous GPR35 agonist, upregulates PDGFA expression, subsequently activates the PI3K‐AKT pathway, and suppresses neuroinflammation, collectively contributing to reduced epileptogenesis and improved cognitive performance. Importantly, the beneficial effects of L‐Kyna on seizure suppression and cognitive recovery were dependent on GPR35. These findings provide a mechanistic foundation for exploring GPR35‐targeted agonists as potential therapies for epilepsy and its associated comorbidities in future clinical trials.

## 4 Conclusion

Our results demonstrate that GPR35 suppresses neuroinflammation and mitigates epileptogenesis. Mechanistically, GPR35 interacts with PDGFA and inhibits its ubiquitination and subsequent degradation, thereby attenuating epilepsy development. This study elucidates, for the first time, the critical molecular mechanism linking GPR35 to neuroinflammation and epilepsy pathogenesis, offering a novel therapeutic target for potential interventions.

## 5 Experimental Section

### 5.1 Animals

C57BL/6 mice were used in the experiments. (Jiangsu Qinglongshan Biotechnology Co. Ltd). The GPR35 conditional knockout mice were *GPR35^f/f^* (Shanghai Southern Model Biological Corporation). Target transgenic mice *(CX3CR1‐Cre;GPR35^f/f^*) were generated by mating *GPR35^f/f^* mice with *CX3CR1^Cre^* mice (Stock No. 200079). Target transgenic mice (*CaMKIIα‐Cre;GPR35^f/f^*) were obtained by mating *GPR35^f/f^* mice with *CaMKIIα^Cre^* mice (Stock No. 005359). All mice (7–8 weeks old) were housed in individually ventilated cages at 23°C–25°C under a 12 h light‐dark cycle, with ad libitum access to food and water.

### 5.2 Analysis of Human snRNA‐seq Data Integration

Doublet removal and quality control (QC) were performed using Scanpy; batch correction was conducted using SCVI, and normalization and dimensionality reduction were performed using RAPIDS–single cell. Leiden clustering (resolution = 0.6) and UMAP scatter plots were generated. QC thresholds were based on median absolute deviation (MAD): log1p‐transformed total counts (Median ± 5 × MAD), log1p‐transformed gene counts (Median ± 5 × MAD), and percentage of counts in top 20 genes (Median ± 5 × MAD). Cell‐type annotation was conducted using SCSA, and GPR35 across cell types was visualized using Scanpy.

### 5.3 Seizure Models

#### 5.3.1 KA‐Induced Epilepsy Model

Adult C57BL/6J mice and transgenic mice were received KA (HY‐N2309, MedChemExpress). KA was freshly prepared under light‐protected conditions by dissolving 10 mg of KA powder in 5 mL of sterile saline to obtain a 2 mg mL^−1^ working solution. Mice were intraperitoneally injected with the working solution at a dose of 24 mg kg^−1^ body weight. Acute seizures were evaluated using a modified Racine scale (Table S8). Diazepam (2 mg kg^−1^, i.p.; Kelun Pharmaceutical Company, China) was administered 2 h after SE induction to terminate seizures. Mice were maintained for 4 weeks. Racine scores greater than 4 were considered indicative of successful epilepsy induction. If seizures did not reach grade 4 within 60 min, a supplementary dose of KA (1/3 of the initial dose) was administered only once. Animals not successfully induced were excluded from the study.

#### 5.3.2 Pilo‐Induced Epilepsy Model

Mice were received pilocarpine (250 mg kg^−1^, i.p.; HY‐b0726, MedChemExpress). Acute seizures were assessed using a modified Racine scale. Diazepam (2 mg kg^−1^, i.p., Kelun Pharmaceutical Company, China) was administered 2 h after SE induction, and mice were maintained for 4 weeks. Racine scores greater than 4 were considered indicative of successful epilepsy induction. If seizures did not reach grade 4 within 60 min, a supplementary dose of pilocarpine (1/3–1/2 of the initial dose) was administered only once. Animals not successfully induced were excluded from the study.

#### 5.3.3 Surgical Procedure and EEG Recording

Mice were anesthetized with pentobarbital sodium (80 mg kg^−1^, i.p) and positioned in a stereotaxic apparatus. Surface electrodes were implanted over the frontoparietal cortex, and electromyography (EMG) electrodes were placed subcutaneously in the neck. Electrodes were fixed with dental cement.

Mice were individually housed for 7 days. Electrodes were connected to a Pinnacle Technology data acquisition system for continuous 72 h EEG monitoring. Electrographic seizures were analyzed using Sirenia Seizure Pro (Pinnacle Technology), with seizure events defined as high‐amplitude waveforms (≥ 2 × baseline) lasting for ≥10 s.

#### 5.3.4 Chemogenetic Activation of Hippocampal Neurons

Mice were anesthetized with pentobarbital sodium (80 mg kg^−1^, i.p) and mounted in a stereotaxic frame. Either rAAV‐hSyn‐EGFP‐WPRE‐hGH polyA or rAAV‐hSyn‐hM3D(Gq)‐EGFP‐WPRE‐hGH polyA (BrainVTA Technology) was infused bilaterally into the dorsal hippocampus (coordinates: −2.06 mm anteroposterior (AP), ±2.35 mm mediolateral (ML), and −2.25 mm dorsoventral (DV) relative to bregma; 1 µL per hemisphere) over a 10 min. The micropipette remained in place for 5 min to prevent reflux. After surgery, the incisions were sutured and disinfected. Three weeks later, mice were administered clozapine‐N‐oxide (CNO; 2 mg kg^−1^, i.p.; CNO‐01, BrainVTA) every other day for 2 weeks.

#### 5.3.5 Seizure Analysis

Electrographic seizure activity was detected and analyzed using Sirenia Seizure Pro software (v2.2.8, Pinnacle Technology). Quantitative analysis of spontaneous recurrent seizures was performed on continuous video‐EEG recordings acquired during the chronic epileptic phase (≥ 4 weeks after kainic acid‐induced status epilepticus). Electrographic seizures were identified based on characteristic high‐amplitude, rhythmic polyspike or spike‐wave discharges and were required to meet all of the following criteria: (i) frequency ≥ 5 Hz, (ii) amplitude ≥ 2× baseline, and (iii) duration ≥ 10 s. Each detected event was verified manually to exclude movement or artifact‐related signals. Seizure metrics included daily seizure frequency (total number of seizures per 24 h), seizure duration (ictal onset‐to‐offset interval, determined by automated detection and confirmed by manual review), and total seizure burden (cumulative time in seizure per 24 h). Power spectral density heatmaps (0−100 Hz) were generated using the software's spectral analysis module to visualize dynamic changes in spectral power associated with seizure activity across experimental groups.

### 5.4 Neurobehavioral Assessments

#### 5.4.1 OFT

Mice were placed in a 40 × 40 × 40 cm open‐field arena with opaque walls for a 10‐min exploration session. Prior to testing, animals were habituated to the testing room for 30 min daily over 3 days to minimize stress. The illumination at the arena center was maintained at 120 ± 5 lux (63099, RWD Life Science, Shenzhen, China), and the center zone was defined as a 20 × 20 cm square. The arena was cleaned with 75% ethanol between trials. Behavior was recorded using an overhead high‐definition camera and analyzed automatically (Smart 3.0 video tracking system) for the following parameters: total distance traveled, distance moved in the center, time spent in the center, and total movement duration.

#### 5.4.2 NOR

The test consisted of three phases. During the adaptation phase, mice were placed in an empty chamber for 10 min per day over 3 consecutive days. On Day 4, the familiarization phase was conducted, during which mice underwent a 2‐min acclimation period followed by 10 min of exploration with two identical objects. After a 10‐min delay, the testing phase was performed, during which mice were allowed to explore one familiar object and one novel object for 10 min. The chamber and objects were cleaned between sessions. Exploration time for familiar vs. novel objects was quantified, and preferential exploration of the novel object indicated intact recognition memory.

#### 5.4.3 Y Maze

The maze consisted of three arms measuring 30 × 6 × 15 cm. In the acquisition phase, one arm was closed, and mice were allowed to freely explore, remaining two arms for 15 min. The test phase was conducted 1 h later, with all arms opened, and the amice allowed to explore the entire maze for 10 min. The time and distance explored in each arm were recorded. For those with memory impairment, the time spent and distance traveled in each arm were recorded, and reduced exploration of the novel arm was interpreted as an indication of memory impairment.

#### 5.4.4 Morris Water Maze

The Morris water maze test was performed in a circular pool (110 cm diameter) filled with water maintained at 20°C–22°C, with a 10 cm diameter escape platform submerged 1 cm below the water surface. During the 5‐day training phase, each mouse was released from four different starting points each day and allowed 60 s to locate the platform. If a mouse failed to reach the platform within this time, it was guided to it and allowed to remain there for 10 s. Escape latency was recorded, with longer latencies indicating impaired learning. In the testing phase, the platform was removed, and mice were allowed to swim freely for 60 s. The number of crossings over the former platform location and the time spent in the target quadrant were recorded, with reduced time in the target quadrant and fewer crossings indicating memory impairment.

### 5.5 Quantitative Proteomic Analysis

#### 5.5.1 Protein Extraction

Protein extraction was conducted by homogenizing samples in lysis buffer (1% SDS, 8 m urea) supplemented with protease inhibitors, followed by vortexing and mechanical disruption using a high‐throughput tissue grinder (FastPrep‐24TM5G, MP Biomedicals). After incubation at 4°C for 30 min with intermittent mixing, the lysate was centrifuged at 16 000 × *g* for 20 min at 4°C, and supernatant protein concentrations were quantified using the BCA assay (Pierce). For proteomic analysis, proteins were processed using an optimized workflow: denaturation in lysis buffer (95°C, 10 min), reduction with 10 mm TCEP, alkylation with 40 mM iodoacetamide, and tryptic digestion (Promega, 37°C, 2 h). Peptides were purified using C18 SPE columns and quantified using a colorimetric peptide assay (Pierce).

#### 5.5.2 LC‐MS/MS Analysis

LC‐MS/MS was performed on a TimsTOF Pro 2 mass spectrometer (Bruker Daltonics) coupled to a nanoflow LC system (UltiMate 3000, Thermo Scientific). A total of 200 ng of peptides were loaded onto a reverse‐phase analytical column (AUR3‐15075C18, 75 µm × 15 cm, IonOpticks) and separated using a 60‐min gradient (4%–90% acetonitrile/0.1% formic acid) at 400 nL/min. Data‐independent acquisition (DIA) was conducted in diaPASEF mode over m/z 349–1229 using 22 isolation windows (40 Th each) with mobility‐dependent collision energies (20–59 eV). For spectral library construction, high‐pH fractionated peptides were analyzed on an Orbitrap Fusion Lumos instrument (Thermo) using high‐resolution MS1 (120 000) and MS/MS (15 000) scans.

#### 5.5.3 Data Analysis

Raw data were analyzed in Spectronaut 18 (Biognosys) using strict quality control criteria: trypsin specificity, carbamidomethylation (fixed), methionine oxidation (variable), and dynamic retention‐time calibration. Protein identification applied a 1% FDR at peptide and protein levels using a mutated decoy strategy. Quantitative comparisons were performed using the MaxLFQ algorithm with local normalization. Functional annotation included Gene Ontology (GO), KEGG, and COG/KOG classifications, and extended analysis incorporated Pfam domain prediction, transcription‐factor classification (plantTFDB/animalTFDB), and subcellular localization prediction (WoLFPSORT). Differentially expressed proteins were identified using Student's *t* test with Benjamini‐Hochberg correction (FDR < 0.05, |fold change| > 1.2), followed by enrichment analysis (hypergeometric test) and PPI network construction (STRING, Cytoscape).

#### 5.5.4 MD Dynamics Simulation

MD simulations of receptor protein‐protein complexes were performed using GROMACS 2020 with the AMBER99SB‐ILDN force field and the TIP3P water model. Systems were solvated with a 1.0 nm buffer distance between protein atoms and box edges, and neutralized with Na^+^/Cl^−^ ions. The simulation protocol consisted of: (1) two‐stage—first with constrained protein heavy atoms (10 000 steps: 5000 steepest‐descent + 5000 conjugate‐gradient on water) followed by full‐system minimization using the same scheme; (2) gradual heating to 300 K over 50 ps; (3) 50 ps equilibration under NPT conditions; and (4) 100 ns production simulation under NPT with trajectory snapshots saved every 5 ps. Binding free energies were calculated using gmxMMPBSA in GROMACS 2020.

#### 5.5.5 Microglia‐Selective Overexpression and Neuron‐Selective Knockdown of PDGFA

Viral injections were administered to *GPR35^f/f^*, *CX3CR1‐Cre;GPR35^f/f^* and *CaMKIIα‐Cre;GPR35^f/f^* mice. For microglia‐specific PDGFA manipulation, rAAV‐SFFV‐DIO‐PDGFA‐P2A‐mCherry‐WPRE‐hGH pA (6.32 × 10^12^ viral particles mL^−1^) or rAAV‐SFFV‐DIO‐mCherry‐WPRE‐hGH pA (5.01 × 10^12^ viral particles mL^−1^) was bilaterally injected into CA1 (AP: −2.0 mm; ML: ±1.5 mm; DV: −1.5 mm). For neuron‐specific PDGFA knockdown, rAAV‐*CaMKIIα‐Cre*‐WPRE‐pA (5.67 × 10^12^ viral particles mL^−1^) or rAAV‐CMV‐DIO‐(EGFP‐U6)‐shRNA(PDGFA)‐WPRE‐hGH polyA (5.07 × 10^12^ viral particles mL^−1^) was injected at the same coordinates. Following microinjection, pipettes were left in place for 5 min to prevent reflux. Incisions were sutured and disinfected. Immunofluorescence and Western blot experiments were performed 3 weeks post‐injection to confirm the efficiency of PDGFA protein knockdown or overexpression efficiency.

### 5.6 Cell Culture

#### 5.6.1 BV2 Cells Culture

BV2 microglial cells (murine microglial cell line) were obtained from PuRuiNuoSai Company (product number: CL‐0493A). Cells were cultured in DMEM (HyClone, SH300022.01B) supplemented with 10% fetal bovine serum (FBS) (Lonsera, S711‐001) and 1% penicillin‐streptomycin (Beyotime, C0222) at 37°C in a humidified incubator with 5% CO_2_. Medium was replaced every 2–3 days. At 80%–90% confluence, cells were detached using 0.25% trypsin‐EDTA (Beyotime, C0201) and subcultured at an appropriate split ratio. For experiments, cells were seeded in suitable plates and grown to the desired confluence before treatment.

#### 5.6.2 HEK293T Cells Culture

HEK293T cells (SV40 large T‐antigen‐transformed human embryonic kidney cells) were obtained from CellCook Biotech Co., Ltd. (product number: CC4003) and maintained in high‐glucose DMEM (HyClone, SH300022.01B) supplemented with 10% FBS (Lonsera, S711‐001) at 37°C in a humidified incubator with 5% CO_2_. Medium was replaced every 2–3 days. Cells were passaged at 80%–90% confluence using 0.25% trypsin‐EDTA and subcultured at a 1:3–1:5 split ratio. Detached cells were allowed to reattach by leaving the culture vessel undisturbed at 37°C. For experiments, cells in the logarithmic growth phase were seeded in appropriate plates and grown to the desired confluence prior to treatment.

#### 5.6.3 Primary Microglia Culture

Primary microglial cells were isolated from the hippocampi of postnatal day 0 C57BL/6 mice. Hippocampal tissue was dissected, minced, and digested with 0.125× trypsin (Beyotime, C0201) in PBS (Lonsera, S711‐001) at 37°C for 20 min, with digestion terminated by the addition of FBS. The tissue was mechanically dissociated, filtered through a 100 µm cell strainer (BD Falcon, 352360), and centrifuged at 500 × g for 10 min at 4°C. Cells were resuspended in DMEM/F12 (Gibco, 11320033) supplemented with 10% FBS and 1% penicillin‐streptomycin, then plated and incubated at 37°C with 5% CO_2_. Medium was replaced after 24 h and every 4 days thereafter. After 10–14 days, microglia were collected by gentle shaking (200 rpm, 2 h), seeded into appropriate plates, and allowed to adhere prior to experimental treatments.

#### 5.6.4 Western Blotting

Mouse brain proteins were separated by 10% SDS–PAGE and transferred to a BioTrace NT nitrocellulose membrane (#10600001, Cytiva, Massachusetts, USA). Membranes were blocked in 5% bovine serum albumin (#4240GR100, BioFroxx) for 2 h at room temperature and then incubated overnight at 4°C with the following primary antibodies: anti‐GPR35 (1:1000, rabbit, NBP2‐24640SS, Novus Biologicals, Littleton, CO, USA), anti‐PDGFA (1:100, mouse, A19548, Santa Cruz, California, USA), anti‐phospho‐TAZ resistant (Ser893) (1:1000, rabbit, 59971 S, Cell Signaling Technology, Danvers, MA, USA), anti‐phospho‐YAP (Ser127) (1:1000, rabbit, 13 008 T, phospho‐Yap (Ser127), Cell Signaling Technology, Danvers, MA, USA), anti‐β‐actin (1:500, rabbit, AF5006, Beyotime, Shanghai, China), and anti‐GAPDH (1:2000, AF1186, Beyotime, Shanghai, China). After washing three times with TBST, membranes were incubated with secondary antibodies for 1–2 h and detected using chemiluminescent HRP substrate (#WBKLS0050, Merck‐Millipore, Burlington, MA, USA). Blots were imaged using the ChemiDoc XRS system (Bio‐Rad, Hercules, California, USA), and band intensities were quantified using ImageJ. Protein levels were normalized to β‐actin or GAPDH, which served as loading controls.

#### 5.6.5 Immunofluorescence Staining

Tissue sections and cultured cells were fixed with 4% paraformaldehyde, permeabilized with 0.1% Triton X‐100 in PBS for 30 min, and blocked with 3% goat serum for 2 h. Brain slices were incubated overnight at 4°C with primary antibodies: anti‐NeuN (1:200, mouse, #94403S, Cell Signaling Technology) and anti‐GPR35 (1:200, rabbit, #NBP2‐24640SS, Novus Biologicals). After washing with PBS, samples were incubated for 2 h at room temperature (in the dark) with the following secondary antibodies: DyLight 594‐conjugated goat anti‐rabbit IgG (1:500, #E032420‐01, EarthOx) or DyLight 488‐conjugated goat anti‐mouse IgG (1:500, #E032210‐01, EarthOx). Nuclei were counterstained with DAPI (#P0131‐25 mL, Beyotime) and images were acquired using a ZEISS LSM800 confocal microscope.

#### 5.6.6 Nissl's Staining

The cryosectioned brain slices were rinsed with distilled water and dried in a 37°C oven. Sections were then covered with Nissl staining solution and incubated at 37°C for 8 min, followed by two washes with distilled water. Dehydration was performed using a graded ethanol series (70%, 90%, and 100% ethanol, 1 min each). The sections were cleared in xylene for 2 min, rinsed in absolute ethanol for 1 min, and washed with distilled water before being mounted with glycerol for fluorescence microscopy.

#### 5.6.7 Quantitative PCR

Total RNA was extracted using TRIzol reagent (Thermo Fisher Scientific) and reverse transcribed into cDNA with Hifair III first Strand cDNA Synthesis SuperMix (Yeasen). Quantitative PCR was performed using Hieff UNICON qPCR SYBR Green Master Mix (Yeasen) on a Roche LightCycler 480 system. The 20 µL reaction mixture contained 10 µL Master Mix, 0.4 µm of each primer, and 2 µL cDNA. Amplification conditions were: 95°C for 5 min; 40 cycles of 95°C for 10 s, 60°C for 20 s, and 72°C for 20 s. Melt curve analysis confirmed amplification specificity. Gene expression was normalized to GAPDH and analyzed using the 2^−ΔΔ^CT method. Table S9 lists all primers.

#### 5.6.8 Immunoprecipitation (IP)

Cells were lysed in IP lysis buffer supplemented with phenylmethylsulfonyl fluoride (PMSF; Beyotime, ST505) and a protease inhibitor cocktail (Biosharp, P1005; China). A total of 500 µg of whole‐cell lysate protein was pre‐cleared using 1.0 µg of PDGFA antibody (overnight incubation at 4°C), with IgG serving as a control. The mixture was subsequently incubated with Protein A/G agarose beads (Beyotime, P2012; China) for 5 h at 4°C. After three washes with cold PBS (pH 7.4), bound proteins were eluted by boiling in 2 × SDS loading buffer and analyzed by Western blotting.

#### 5.6.9 ELISA

Cell samples were processed for total protein extraction according to the manufacturer's protocol. Protein concentration was determined using a BCA assay kit, and samples were diluted with an appropriate buffer to approximately 0.5 µg/µL. Cytokine levels (IL‑6, IL‑1β, TNF‑α, and IL‑10) were quantified using a mouse‐specific ELISA kit (E‑EL‑M0044c, Elabscience) following the manufacturer's instructions. All samples were analyzed in at least three independent replicates.

#### 5.6.10 Ubiquitination Assays

BV2 cells were treated with cycloheximide (CHX; 200 µg/mL) for 3, 6, 12, and 24 h to assess PDGFA protein stability; vehicle‐treated cells served as controls. PDGFA protein levels were analyzed by Western blotting. For proteasome inhibition, MG132 (10 µm, MCE, HY‐13259) was added to BV2 cells for 6 h, followed by lysis win IP buffer for 30 min. Lysates were immunoprecipitated overnight at 4°C with anti‐PDGFA antibody or IgG. Protein A/G agarose beads were added and incubated for 2 h at 4°C. After five washes, proteins were eluted by boiling in 2× SDS loading buffer and analyzed by Western blotting with anti‐ubiquitin antibody (ET1609‐21).

### 5.7 Statistical Analysis

All statistical analyses were performed using GraphPad Prism (GraphPad Software, USA). Data normality was assessed using the Shapiro‐Wilk test. For normally distributed data, comparisons were conducted using two‐tailed paired or unpaired Student's *t*‐tests, one‐way analysis of variance (ANOVA), two‐way ANOVA, or two‐way repeated‐measures ANOVA, as appropriate. When ANOVA revealed significant effects, Bonferroni's post hoc test was applied for multiple comparisons. For data not following a normal distribution, appropriate non‐parametric tests with corresponding post hoc pairwise comparisons were employed.

All data are presented as mean ± standard error of the mean (SEM), and sample sizes (n) are specified in the corresponding figure legends. Although sample sizes were not determined by prior statistical power analysis, they were consistent with those reported in comparable studies. Experimental procedures, including animal assignment and treatment order, were randomized. Investigators were blinded to genotype and treatment group during data collection and analysis. Imaging and acquisition settings were maintained consistently across all experimental conditions to minimize technical variability.

## Author Contributions

Yu Wang, Zhimig Zhou, and Bin Wang conceived the project concept. Qi Wang, Bin Wang, Zhiming Zhou, and Yu Wang designed the study. Tinging Qu, Lei Wang, Hanli Li, Jianyun Sun, Fangliang Chen, Ziyin Xuan, Xiangjian Lei, Jinshuai Liu, and Zifan Yang developed the methodology. Qi Wang, Tinging Qu, Ran Li, Qibing Sun, and Junfei Dong performed the experiments. Qi Wang, Tinging Qu, Qibing Sun, and Ran Li conducted the initial data analysis. Qian Yang, Ran Li, Qibing Sun, Tinging Qu, and Yuming Du contributed to figure preparation and typesetting. Qi Wang drafted the manuscript. Yu Wang, Zhiming Zhou, Bin Wang, and Qi Wang provided funding support and revised the manuscript. All authors approved the final manuscript.

## Ethics Statement

All animal procedures were approved by the Ethics Committee of Anhui Medical University (Approval number 2019–151, Hefei, China). Experiments were performed in accordance with the National Institutes of Health Guide for the Care and Use of Laboratory Animals, with efforts made to minimize animal use and reduce suffering.

## Conflicts of Interest

The authors declare no conflicts of interest.

## Supporting information




**Supporting File**: advs74012‐sup‐0001‐SuppMat.pdf.

## Data Availability

The data supporting the findings of the present study are available from the corresponding authors upon request.
