# Supplementary material for: Microglial GPR35 Ameliorates Epileptogenesis and Neuroinflammation via PDGFA Domain 2 Signaling
Source: Adv Sci (Weinh). 2026 Jan 28;13(19):e19642. doi: 10.1002/advs.202519642 (PMC13045304; doi:10.1002/advs.202519642)

Supporting Information

for

Microglial GPR35 Ameliorates Epileptogenesis and Neuroinflammation via PDGFA domain 2 Signaling

Qi Wang[1,2#], Tingting Qu[1,3#], Qibing Sun[1#], Ran Li[1#], Junfei Dong[2], Yuming Du[2], Ziyin Xuan[1], Lei Wang[1], Hanli Li[1], Jianyun Sun[1], Fangliang Chen[1], Jinshuai Liu[1], Zifan Yang[1], Jianxiang Lei[1], Qian Yang[2], Bin Wang[4], Zhiming Zhou[2*], Yu Wang[1,3*]*

Supplemental Tables

Table S1. Primer information for quantitative real-time PCR.
(*GPR35^{KO}*)

Primer	Sequence (5'→3')	Primer Type
**P1 (*Gpr35^{KO}*)**	GACTCAGGCTCTAACGTCCG	Wild type
**P2 (*Gpr35^{KO}*)**	CCCTTTTGCCCAACCCTACA	Common
**P3 (*Gpr35^{KO}*)**	GGGTTTGGCCCTTAGGATGA	Mutant

Table S2. Primer information for quantitative real-time PCR.
(*CaMKIIα-Cre;GPR35$^{f/f}$*)

Primer	Sequence (5'→3')	Primer Type
**P1 (*Gpr35^{flox}*)**	GACTCAGGCTCTAACGTCCG	Forward
**P2 (*Gpr35^{flox}*)**	CCCTTTTGCCCAACCCTACA	Reverse
**P3 (*CamKIIα-Cre*)**	GGGGAGGTAGGAAGAGCGATGA	Transgene Forward
**P4 (*CamKIIα-Cre*)**	CATCGACCGGTAATGCAG	Transgene Reverse
**P5 (*CamKIIα-Cre*)**	CAAATGTTGCTTGTCTGGTG	Internal Positive Control Forward
**P6 (*CamKIIα-Cre*)**	GTCAGTCGAGTGCACAGTTT	Internal Positive Control Reverse

Table S3. Primer information for quantitative real-time PCR.
(*CX3CR1-Cre;GPR35$^{f/f}$*)

Primer	Sequence (5'→3')	Primer Type
**P1 (*Gpr35^{flox}*)**	GCCTATCTGGCAGACCATCC	Forward
**P2 (*Gpr35^{flox}*)**	CCCCCTTTTCCACCTGGTT	Reverse
**P3 (*CX3CR1-Cre*)**	AGGATGAGTCTGACGGCTCT	Wild type
**P4 (*CX3CR1-Cre*)**	GTGTTTTCTCCCGCTTGCTG	Common
**P5 (*CX3CR1-Cre*)**	GTTGTTCAGCTTGCACCAGG	Mutant

Table S4. The molecular dynamics results of two target protein.

Protein1	Protein2	Contact Sites (protein1)	Contact Sites (protein2)	Combination Type
**GPR35**	**PDGFA**	SER-298, HIS-296, ASP-204, SER-292, ASN-293, ALA-203, GLN-207, ARG-121, THR-106, TYR-113, ARG-123	ASN-72, ALA-65, ARG-84, SER-62, ALA-58, GLN-45, GLU-49, GLU-24, TRP-4, LEU-15, GLU-28	Salt bridge, Hydrogen bond, Hydrophobic interaction

Table S5. The binding energy by MMGBSA (kcal/mol).

Type	Complex
$\boldsymbol{EVDW}$ (van der Waals energy)	-206.89 ± 9.7
$\boldsymbol{E_{ELE}}$ (eletrostatic energy)	-230.67 ± 70.58
$\boldsymbol{E_{GB}}$ (polar solvation energy)	284.09 ± 57.62
$\boldsymbol{E_{SA}}$ (solvent accessible surface energy)	-22.29 ± 0.49
$\boldsymbol{G_{binding\ energy}}$	-175.76 ± 61.63

Table S6. All information related to plasmid.

Manufacturers	Sample ID	Plasmid name
MiaoLingPlasmid	G57384	pCMV-Pdgfa(mouse)-3 × FLAG-Neo
MiaoLingPlasmid	G57383	pCMV-Pdgfa(mouse)(185-211aa)-3 × FLAG-Neo
MiaoLingPlasmid	G57382	pCMV-Pdgfa(mouse)(87-211aa)-3 × FLAG-Neo
MiaoLingPlasmid	G57381	pCMV-Pdgfa(mouse)(1-185aa)-3 × FLAG-Neo
MiaoLingPlasmid	G57380	pCMV-Pdgfa(mouse)(1-87aa)-3 × FLAG-Neo
MiaoLingPlasmid	G57384	pCMV-Pdgfa(mouse)-3 × FLAG-Neo
MiaoLingPlasmid	P37795	HA-Ubiquitin

Table S7. All information related to viruses.

Manufacturers	Sample ID	Sample Name
BrainVTA	9-152-K240530	rAAV-hSyn-hM3D(Gq)-EGFP-WPRE-hGH polyA
BrainVTA	9-190-S240229	rAAV-hSyn-EGFP-WPRE-hGH polyA

BrainVTA	9-2644-S240407	rAAV-CMV-DIO-(EGFP-U6)-shRNA(scramble) -WPRE-hGH polyA (5’ to 3’) CCTAAGGTTAAGTCGCCCTCG
BrainVTA	9-11912-K241022	rAAV-CMV-DIO-(EGFP-U6)-shRNA(PDGFA)-WPRE-hGH polyA (5’ to 3’) GGTGGCCAAAGTGGAGTATGT
BrainVTA	MG1.2-8295-K240514	rAAV-SFFV-DIO-EGFP-5'miR-30a-shRNA(scramble) -3'miR-30a-WPREs(5’ to 3’) CCTAAGGTTAAGTCGCCCTCG
BrainVTA	MG1.2-11835-K241011	rAAV-SFFV-DIO-EGFP-5'miR-30a-shRNA1 (PDGFA) -3'miR-30a-WPREs(5’ to 3’) GGTGGCCAAAGTGGAGTATGT
BrainVTA	CNO-01	CNO-231020
BrainVTA	MG1.2-13040-K250225	rAAV-SFFV-DIO-PDGFA-P2A-mCherry-WPRE-hGH pA
BrainVTA	MG1.2-7282-K250225	rAAV-SFFV-DIO-mCherry-WPRE-hGH pA
GenePharma	LV3-Gpr35-Mus-1100	LV-Gpr35 (5’ to 3’) AGGTCTCCCTGAACCTCAATA
GenePharma	LV3-NC	LV-Scr (5’ to 3’) TTCTCCGAACGTGTCACGT

Table S8. modified Racine scale (Racine Score).

Tests	Score
freezing behavior	1
rigid posture with raised tail	2
continuous head bobbing and forepaw shaking	3
rearing, falling and jumping	4
continuous rearing or jumping	5
loss of posture, generalized convulsion activity or death	6
Mice with scores greater than 4 were considered successful models and then included in subsequent researches.	

Table S9. Primer information for quantitative real-time PCR.

Target gene	Forward (5' to 3')	Reverse (5' to 3')
m-IL10_qPCR_74bp	TGGACAACATACTGCTAACCGA	TTCCGATAAGGCTTGGCAAC
m-IL1β_qPCR_135bp	ACCTGTTCTTTGAAGTTGACGGA	ATACTGCCTGCCTGAAGCTCT
m-IL6_qPCR_134bp	CTTCTTGGGACTGATGCTGGT	CATGTGTAATTAAGCCTCCGACT

m-TNFα_qPCR_92bp	AAGGGAGAGTGGTCAGGTTGC	CAGGGAAGAATCTGGAAAGGTC
m-Gpr35_qPCR_122bp	CATTGCTGTGGACCGCTATGT	CTACCAGGGAGGTGACCACTATC
m-PDGF-A_qPCR_120bp	GATGTGAGGTGAGATGAGCCG	GAGAACAAAGACCGCACGC
GAPDH	GAACGGGAAGCTCACTGG	GCCTGCTTCACCACCTTCT

Table S10. Hippocampal GPR35 protein in sham and KA-induced mice. Numeric data on Figure 1F.

Figure 1F	Group	Relative TNF-α/Actin density	DF	*t*	p
**1d**	**Sham**	0.415 ± 0.030	-	-	-
	**KA**	0.623 ± 0.020	5.118	5.770	0.0121
**3d**	**Sham**	0.393 ± 0.022	-	-	-
	**KA**	0.700 ± 0.056	3.910	5.072	0.0445
**7d**	**Sham**	0.381 ± 0.032	-	-	-
	**KA**	0.864 ± 0.069	4.201	6.356	0.0158
**2w**	**Sham**	0.419 ± 0.027	-	-	-
	**KA**	1.197 ± 0.086	3.605	8.661	0.0092
**4w**	**Sham**	0.429 ± 0.025	-	-	-
	**KA**	1.520 ± 0.065	3.880	15.58	0.0007
**8w**	**Sham**	0.462 ± 0.028	-	-	-
	**KA**	1.196 ± 0.045	5.091	13.90	0.0002

Table S11. Hippocampal GPR35 mRNA in 4wsham and 4wKA mice. Numeric data on Figure 1G.

Fig 1G	Relative GPR35 mRNA in the hippocampus	DF	*t*	p
**4wSham**	0.923 ± 0.077	-	-	-
**4wKA**	1.596 ± 0.069	8	6.532	0.0002

Table S12. Cell viability of BV2 cells treated with glutamate. Numeric data on Figure 1J.

	Figure 1J	5 mM	10 mM	20 mM
	**0h**	1.00 ± 0.00	1.00 ± 0.00	1.00 ± 0.00
	**6h**	0.985 ± 0.035	0.901 ± 0.029	0.726 ± 0.021
	**12h**	0.711 ± 0.009	0.562 ± 0.025	0.353 ± 0.018
	**24h**	0.555 ± 0.002	0.475 ± 0.010	0.421 ± 0.006
		**DF**	**q**	**p**
**6h**	**5 mM *vs.* 10 mM**	3.838	2.618	0.2697
	**5 mM *vs.* 20 mM**	3.250	8.954	0.0130
	**10 mM *vs.* 20 mM**	3.659	7.000	0.0207

**12h**	**5 mM *vs.* 10 mM**	2.531	8.081	0.0322
	**5 mM *vs.* 20 mM**	2.914	24.74	0.0010
	**10 mM *vs.* 20 mM**	3.703	9.606	0.0070
**24h**	**5 mM *vs.* 10 mM**	2.085	11.14	0.0256
	**5 mM *vs.* 20 mM**	2.271	32.87	0.0016
	**10 mM *vs.* 20 mM**	3.137	6.733	0.0324
**5 mM**	**0h *vs.* 6h**	2	0.6060	0.9681
	**0h *vs.* 12h**	2	45.18	0.0012
	**0h *vs.* 24h**	2	431.0	<0.0001
	**6h *vs.* 12h**	2	13.70	0.0261
	**6h *vs.* 24h**	2	17.98	0.0154
	**12h *vs.* 24h**	2	28.63	0.0063
**10 mM**	**0h *vs.* 6h**	2	4.900	0.1772
	**0h *vs.* 12h**	2	25.22	0.0081
	**0h *vs.* 24h**	2	74.04	<0.0001
	**6h *vs.* 12h**	2	19.29	0.0135
	**6h *vs.* 24h**	2	32.35	0.0046
	**12h *vs.* 24h**	2	6.060	0.1223
**20 mM**	**0h *vs.* 6h**	2	18.59	0.0144
	**0h *vs.* 12h**	2	49.79	0.0007
	**0h *vs.* 24h**	2	146.4	<0.0001
	**6h *vs.* 12h**	2	81.87	<0.0001
	**6h *vs.* 24h**	2	27.23	0.0070
	**12h *vs.* 24h**	2	7.431	0.0843

Table S13. GPR35 mRNA in primary microglia induced by varying glutamate concentrations. Numeric data on Figure 1K.

**Figure 1K**	**Relative GPR35 mRNA in Glu-induced primary microglia**		
**0 mM**	0.955 ± 0.052		
**5 mM**	1.002 ± 0.014		
**10 mM**	1.420 ± 0.041		
**20 mM**	1.183 ± 0.077		
	**DF**	***q***	**p**
**0 mM *vs.* 5 mM**	12	3.041	0.0304
**0 mM *vs.* 10 mM**	12	8.697	<0.0001
**0 mM *vs.* 20 mM**	12	1.171	0.6020

Table S14. GPR35 protein in BV2 cells induced with 10 mM glutamate for 12 h compared with controls. Numeric data on Figure 1N.

**Figure 1N**	**GPR35/Actin relative density**	**DF**	***t***	**p**
**Ctrl1**	0.534 ± 0.083	-	-	-
**Glu**	1.030 ± 0.085	10	4.174	0.0019

Table S15. GPR35 protein inprimary microglia induced with 10 mM glutamate for 12 h compared with controls. Numeric data on Figure 1O.

Figure 1O	GPR35/Actin relative density	DF	*t*	p
**Ctrl2**	0.611 ± 0.118	-	-	-
**Glu**	1.488 ± 0.185	6	4.001	0.0071

Table S16. The Racine scale in sham and Pilo mice. Numeric data on Figure 2B.

Figure 2B	Raccine score	DF	*t*	p
**Sham**	0.000 ± 0.000	-	-	-
**Pilo**	3.636 ± 0.244	22	15.65	<0.0001

Table S17. GPR35 protein in 4wsham and 4wPilo mice. Numeric data on Figure 2D.

Figure 2D	GPR35/Actin relative density	DF	*t*	p
**4wSham**	0.480 ± 0.069	-	-	-
**4wPilo**	0.899 ± 0.073	6	4.159	0.0060

Table S18. GPR35 protein in 4wsham and 4wPilo mice. Numeric data on Figure 2E.

Figure 2E		Relative expression	DF	*t*	p
**IL-1β**	**4wSham**	0.975 ± 0.031	-	-	-
	**4wPilo**	1.194 ± 0.049	5.029	3.787	0.0375
**IL-6**	**4wSham**	1.050 ± 0.022	-	-	-
	**4wPilo**	1.494 ± 0.049	4.129	8.233	0.0031
**TNF-α**	**4wSham**	0.966 ± 0.035	-	-	-
	**4wPilo**	3.311 ± 0.181	3.217	12.70	0.0022

Table S19. Hippocampal GPR35 protein in repeated chemogenetic excitation of hippocampal neurons and sham. Numeric data on Figure 2K.

Figure 2K	GPR35/Actin relative density	DF	*t*	p
**Veh**	0.363 ± 0.046	-	-	-
**CNO**	0.805 ± 0.054	6	6.253	0.0008

Table S20. Hippocampal inflammatory cytokines mRNA (IL-1β, IL-6, TNF-α and IL-10) in repeated chemogenetic excitation of hippocampal neurons and sham. Numeric data on Figure 2L.

Figure 2L		Relative expression	DF	*t*	p
**IL-1β**	**Veh**	1.002 ± 0.019	-	-	-
	**CNO**	1.256 ± 0.094	3.252	2.647	0.1979
**IL-6**	**Veh**	1.206 ± 0.142	-	-	-

	**CNO**	2.725 ± 0.155	5.953	7.211	0.0011
**TNF-α**	**Veh**	0.979 ± 0.009	-	-	-
	**CNO**	1.617 ± 0.042	3.250	15.07	0.0012

Table S21. The Racine scale in WT KA and GPR35^{KO} KA mice. Numeric data on Figure 3B.

Figure 3B	Raccine score	DF	*t*	p
**WT KA**	3.8 ± 0.2	-	-	-
**GPR35^{KO} KA**	4.7 ± 0.3	18	2.741	0.0134

Table S22. Electrographic seizure-like in 4wWT KA and 4wGPR35^{KO} KA mice. Numeric data on Figure 3E.

Figure 3E	Group	Data	DF	*t*	p
**No of seizures per day**	**WT KA**	1.44 ± 0.61	-	-	-
	**GPR35^{KO} KA**	4.39 ± 0.83	10	2.850	0.0172
**Seizure duration**	**WT KA**	21.76 ± 8.27	-	-	-
	**GPR35^{KO} KA**	88.22 ± 17.11	10	3.496	0.0058

Table S23. Open field test in 4wWT KA and 4wGPR35^{KO} KA mice. Numeric data on Figure 3G.

Figure 3G	Group	Data	DF	*t*	p
**Time in center (%)**	**WT KA**	12.88 ± 1.62	-	-	-
	**GPR35^{KO} KA**	3.75 ± 1.26	14	4.448	0.0006
**Total distance (unit)**	**WT KA**	20.05 ± 2.99	-	-	-
	**GPR35^{KO} KA**	14.38 ± 1.53	14	1.691	0.1131

Table S24. Novel object recognition in 4wWT KA and 4wGPR35^{KO} KA mice. Numeric data on Figure 3H.

Figure 3H	Group	Data	DF	*t*	p
**Novel object recognition idex**	**WT KA**	0.721 ± 0.035	-	-	-
	**GPR35^{KO} KA**	0.440 ± 0.040	14	5.283	0.0001
**Entries in new zone**	**WT KA**	12.38 ± 1.12	-	-	-
	**GPR35^{KO} KA**	5.50 ± 1.21	14	4.175	0.0009

Table S25. Y maze in 4wWT KA and 4wGPR35^{KO} KA mice. Numeric data on Figure 3I.

Figure 3I	Novel arm pefernce index (%)	DF	*t*	p
**WT KA**	0.316 ± 0.022	-	-	-
**GPR35^{KO} KA**	0.216 ± 0.025	14	2.977	0.0100

Table S26. Hippocampal GPR35 mRNA and GPR35 protein in *GPR35$^{f/f}$*, *CaMKIIα-Cre;GPR35$^{f/f}$* and *CX3CR1-Cre;GPR35$^{f/f}$* mice. Numeric data on Figure 4C.

Fig 4C	Group	Data		
**Relative GPR35 mRNA in the hippocampus**	***GPR35$^{f/f}$***	1.050 ± 0.063		
	CaMKIIα-Cre;GPR35$^{f/f}$	0.566 ± 0.121		
	CX3CR1-Cre;GPR35$^{f/f}$	0.714 ± 0.037		
		**DF**	***q***	**p**
	GPR35$^{f/f}$* vs. *CaMKIIα-Cre;GPR35$^{f/f}$	9	4.213	0.0042
	GPR35$^{f/f}$* vs. *CX3CR1-Cre;GPR35$^{f/f}$	9	2.919	0.0307
**GPR35/Actin relative density**	***GPR35$^{f/f}$***	1.094 ± 0.103		
	CaMKIIα-Cre;GPR35$^{f/f}$	0.614 ± 0.067		
	CX3CR1-Cre;GPR35$^{f/f}$	0.537 ± 0.097		
		**DF**	***q***	**p**
	GPR35$^{f/f}$* vs. *CaMKIIα-Cre;GPR35$^{f/f}$	9	3.773	0.0081
	GPR35$^{f/f}$* vs. *CX3CR1-Cre;GPR35$^{f/f}$	9	4.377	0.0033

Table S27. Hippocampal GPR35 mRNA and GPR35 protein in KA-induced *GPR35$^{f/f}$*, KA-induced *CaMKIIα-Cre;GPR35$^{f/f}$* and KA-induced *CX3CR1-Cre;GPR35$^{f/f}$* mice. Numeric data on Figure 4D.

Fig 4D	Group	Data		
**Relative GPR35 mRNA in the hippocampus**	***GPR35$^{f/f}$***	1.247 ± 0.049		
	CaMKIIα-Cre;GPR35$^{f/f}$	0.654 ± 0.045		
	CX3CR1-Cre;GPR35$^{f/f}$	0.973 ± 0.048		
		**DF**	***q***	**p**
	GPR35$^{f/f}$* vs. *CaMKIIα-Cre;GPR35$^{f/f}$	6	8.866	0.0002
	GPR35$^{f/f}$* vs. *CX3CR1-Cre;GPR35$^{f/f}$	6	4.093	0.0114
**GPR35/Actin relative density**	***GPR35$^{f/f}$***	1.046 ± 0.048		
	CaMKIIα-Cre;GPR35$^{f/f}$	0.648 ± 0.108		
	CX3CR1-Cre;GPR35$^{f/f}$	0.556 ± 0.060		
		**DF**	***q***	**p**
	GPR35$^{f/f}$* vs. *CaMKIIα-Cre;GPR35$^{f/f}$	6	3.684	0.0182
	GPR35$^{f/f}$* vs. *CX3CR1-Cre;GPR35$^{f/f}$	6	4.529	0.0071

Table S28. Electrographic seizure-like inKA-induced *GPR35$^{f/f}$*, KA-induced *CaMKIIα-Cre;GPR35$^{f/f}$* and KA-induced *CX3CR1-Cre;GPR35$^{f/f}$* mice. Numeric data on Figure 4F.

Fig 4F	Group	Data		
No. of seizures per day	*GPR35$^{f/f}$* KA	1.056 ± 0.526		
	CaMKIIα-Cre;GPR35$^{f/f}$ KA	1.328 ± 0.458		
	CX3CR1-Cre;GPR35$^{f/f}$ KA	3.222 ± 0.703		
		DF	*q*	p
	GPR35$^{f/f}$ KA *vs.* *CaMKIIα-Cre;GPR35$^{f/f}$* KA	15	0.3367	0.9212
	GPR35$^{f/f}$ KA *vs.* *CX3CR1-Cre;GPR35$^{f/f}$* KA	15	2.680	0.0314
Seizure duration (sec)	*GPR35$^{f/f}$* KA	35.39 ± 13.96		
	CaMKIIα-Cre;GPR35$^{f/f}$ KA	62.80 ± 13.07		
	CX3CR1-Cre;GPR35$^{f/f}$ KA	121.79 ± 23.50		
		DF	*q*	p
	GPR35$^{f/f}$ KA *vs.* *CaMKIIα-Cre;GPR35$^{f/f}$* KA	15	1.108	0.4526
	GPR35$^{f/f}$ KA *vs.* *CX3CR1-Cre;GPR35$^{f/f}$* KA	15	3.492	0.0062

Table S29. PDGFA protein in 4wSham and 4wKA mice. Numeric data on Figure 5J.

Figure 5J	PDGFA/Actin relative density	DF	*t*	p
4wSham	0.385 ± 0.027	-	-	-
4wKA	0.674 ± 0.056	6	4.663	0.0035

Table S30. PDGFA protein in Glu-treated and control primary microglia from the hippocampal tissue of WT mice. Numeric data on Figure 5K.

Figure 5K	PDGFA/Actin relative density	DF	*t*	p
Ctrl2	0.710 ± 0.050	-	-	-
Glu	0.995 ± 0.030	6	4.902	0.0027

Table S31. GPR35 and PDGFA protein in Glu-treated primary microglia from GPR35^{KO} mice compared with controls. Numeric data on Figure 5L.

Figure 5L	Group		DF	*t*	p
GPR35	Glu	1.248 ± 0.173	-	-	-
	GPR35^{KO} + Glu	0.453 ± 0.025	12.00	5.439	0.0003
GPR35^{KO} + Glu	Glu	1.213 ± 0.075	-	-	-
	GPR35^{KO} + Glu	0.595 ± 0.081	12.00	4.229	0.0023

Table S32. Partial correlation analysis of PDGFA and GPR35 protein expression in Glu-induced primary microglia from GPR35KO mice compared with controls. Numeric data on Figure 5M.

Figure 5M	Data	F	R squared	p
**GPR35**	0.851 ± 0.171	-	-	-
**PDGFA**	0.904 ± 0.128	7.099	0.5419	0.0373

Table S33. PDGFA mRNA in the BV2 cells with transfected LV-GPR35 or LV-Scr2. Numeric data on Figure 6A.

Figure 6A	Relative PDGFA mRNA in the BV2 cells	DF	*t*	p
**LV-Scr2 Glu**	1.080 ± 0.041	-	-	-
**LV-GPR35 Glu**	1.062 ± 0.075	10	0.2069	0.8402

Table S34. PDGFA mRNA in the BV2 cells with transfected LV-shGPR35 or LV-Scr1. Numeric data on Figure 6B.

Figure 6B	Relative PDGFA mRNA in the BV2 cells	DF	*t*	p
**LV-Scr1 Glu**	1.201 ± 0.202	-	-	-
**LV-shGPR35 Glu**	1.230 ± 0.124	6	0.1246	0.9049

Table S35. PDGFA protein in CHX-treated GPR35^{KD} or GPR35^{OE}cells. Numeric data on Figure 6E part 1.

Figure 6E	PDGFA/Actin relative density
**0h LV-shGPR35 Glu**	1.008 ± 0.012
**3h LV-shGPR35 Glu**	0.985 ± 0.023
**6h LV-shGPR35 Glu**	0.623 ± 0.036
**12h LV-shGPR35 Glu**	0.454 ± 0.080
**24h LV-shGPR35 Glu**	0.382 ± 0.044
**0h LV-Scr1 Glu**	0.996 ± 0.045
**3h LV-Scr1 Glu**	0.972 ± 0.016
**6h LV-Scr1 Glu**	0.902 ± 0.001
**12h LV-Scr1 Glu**	0.465 ± 0.037
**24h LV-Scr1 Glu**	0.411 ± 0.111
**0h LV-GPR35 Glu**	1.033 ± 0.008
**3h LV-GPR35 Glu**	1.043 ± 0.042
**6h LV-GPR35 Glu**	0.960 ± 0.025
**12h LV-GPR35 Glu**	0.811 ± 0.031
**24h LV-GPR35 Glu**	0.438 ± 0.039
**0h LV-Scr2 Glu**	1.000 ± 0.113
**3h LV-Scr2 Glu**	0.979 ± 0.081
**6h LV-Scr2 Glu**	0.847 ± 0.082

**12h LV-Scr2 Glu**	0.423 ± 0.058
**24h LV-Scr2 Glu**	0.323 ± 0.143

Table S36. PDGFA protein in CHX-treated GPR35^{KD} or GPR35^{OE}cells. Numeric data on Figure 6E part 2.

Figure 6E		DF	*q*	p
**0h**	**LV-shGPR35 Glu *vs.* LV-Scr1 Glu**	2.289	0.3383	0.9940
	**LV-shGPR35 Glu *vs.* LV-GPR35 Glu**	3.427	2.446	0.4297
	**LV-shGPR35 Glu *vs.* LV-Scr2 Glu**	2.046	0.09556	0.9999
	**LV-Scr1 Glu *vs.* LV-GPR35 Glu**	2.122	1.119	0.8554
	**LV-Scr1 Glu *vs.* LV-Scr2 Glu**	2.619	0.04038	>0.9999
	**LV-GPR35 Glu *vs.* LV-Scr2 Glu**	2.019	0.4080	0.9894
**3h**	**LV-shGPR35 Glu *vs.* LV-Scr1 Glu**	3.587	0.6496	0.9638
	**LV-shGPR35 Glu *vs.* LV-GPR35 Glu**	3.084	1.712	0.6607
	**LV-shGPR35 Glu *vs.* LV-Scr2 Glu**	2.314	0.1048	0.9998
	**LV-Scr1 Glu *vs.* LV-GPR35 Glu**	2.569	2.223	0.5146
	**LV-Scr1 Glu *vs.* LV-Scr2 Glu**	2.156	0.1122	0.9998
	**LV-GPR35 Glu *vs.* LV-Scr2 Glu**	3.002	0.9932	0.8905
**6h**	**LV-shGPR35 Glu *vs.* LV-Scr1 Glu**	2.004	10.81	0.0412
	**LV-shGPR35 Glu *vs.* LV-GPR35 Glu**	3.556	10.76	0.0083
	**LV-shGPR35 Glu *vs.* LV-Scr2 Glu**	2.764	3.533	0.2478
	**LV-Scr1 Glu *vs.* LV-GPR35 Glu**	2.008	3.276	0.3323
	**LV-Scr1 Glu *vs.* LV-Scr2 Glu**	2.001	0.9515	0.8997
	**LV-GPR35 Glu *vs.* LV-Scr2 Glu**	2.376	1.875	0.6204
**12h**	**LV-shGPR35 Glu *vs.* LV-Scr1 Glu**	2.801	0.1788	0.9991
	**LV-shGPR35 Glu *vs.* LV-GPR35 Glu**	2.571	5.864	0.0927
	**LV-shGPR35 Glu *vs.* LV-Scr2 Glu**	3.626	0.4402	0.9879
	**LV-Scr1 Glu *vs.* LV-GPR35 Glu**	3.877	10.21	0.0075
	**LV-Scr1 Glu *vs.* LV-Scr2 Glu**	3.397	0.8683	0.9218
	**LV-GPR35 Glu *vs.* LV-Scr2 Glu**	3.051	8.396	0.0274
**24h**	**LV-shGPR35 Glu *vs.* LV-Scr1 Glu**	2.619	0.3512	0.9934
	**LV-shGPR35 Glu *vs.* LV-GPR35 Glu**	3.930	1.366	0.7753
	**LV-shGPR35+Glu *vs.* LV-Scr2 Glu**	2.378	0.5500	0.9760
	**LV-Scr1 Glu *vs.* LV-GPR35 Glu**	2.478	0.3256	0.9947
	**LV-Scr1 Glu *vs.* LV-Scr2 Glu**	3.766	0.6866	0.9582
	**LV-GPR35 Glu *vs.* LV-Scr2 Glu**	2.290	1.097	0.8616

Table S37. IBA1 and GFAP protein in hippocampus from KA-induced *CX3CR1-Cre;GPR35$^{f/f}$* and KA-induced *GPR35$^{f/f}$* mice. Numeric data on Figure 7C.

Figure 7C	Group	Data	DF	*t*	p
IBA1	*CX3CR1-Cre;GPR35$^{f/f}$*	0.513 ± 0.059	-	-	-
	CX3CR1-Cre;GPR35$^{f/f}$ + KA	1.240 ± 0.105	11	6.706	<0.0001
GFAP	*CX3CR1-Cre;GPR35$^{f/f}$*	0.616 ± 0.054	-	-	-
	CX3CR1-Cre;GPR35$^{f/f}$ + KA	1.170 ± 0.075	11	5.405	0.0004

Table S38. GPR35 and PDGFA protein in hippocampus from KA-induced *CX3CR1-Cre;GPR35$^{f/f}$* and KA-induced *GPR35$^{f/f}$* mice. Numeric data on Figure 7E.

Figure 7E	Group	Relative expression		
GPR35	*GPR35$^{f/f+}$* + PBS	0.503 ± 0.060		
	GPR35$^{f/f}$ + KA	0.754 ± 0.0350		
	CX3CR1-Cre;GPR35$^{f/f}$ + KA	0.260 ± 0.026		
PDGFA	*GPR35$^{f/f}$* + PBS	0.264 ± 0.036		
	GPR35$^{f/f}$ + KA	0.573 ± 0.065		
	CX3CR1-Cre;GPR35$^{f/f}$ + KA	0.078 ± 0.014		
		**DF**	***t***	**p**
GPR35	*GPR35$^{f/f}$* + PBS *vs. GPR35$^{f/f}$* + KA	18	4.090	0.0041
	GPR35$^{f/f}$ + PBS *vs. CX3CR1-Cre;GPR35$^{f/f}$* + KA	18	3.977	0.0053
	GPR35$^{f/f}$ + KA *vs. CX3CR1-Cre;GPR35$^{f/f}$* + KA	18	8.067	<0.0001
PDGFA	*GPR35$^{f/f}$* + PBS *vs. GPR35$^{f/f}$* + KA	18	5.052	0.0005
	GPR35$^{f/f}$ + PBS *vs. CX3CR1-Cre;GPR35$^{f/f}$* + KA	18	3.035	0.0419
	GPR35$^{f/f}$ + KA *vs. CX3CR1-Cre;GPR35$^{f/f}$* + KA	18	8.088	<0.0001

Table S39. p-AKT and p-PI3K protein in hippocampus from KA-induced *CX3CR1-Cre;GPR35$^{f/f}$* and KA-induced *GPR35$^{f/f}$* mice. Numeric data on Figure 7F.

Figure 7F		Relative expression		
p-AKT	*GPR35$^{f/f}$* + PBS	0.828 ± 0.038		
	GPR35$^{f/f}$ + KA	0.649 ± 0.017		
	CX3CR1-Cre;GPR35$^{f/f}$ + KA	0.532 ± 0.051		
p-PI3K	*GPR35$^{f/f}$* + PBS	0.779 ± 0.016		
	GPR35$^{f/f}$ + KA	0.576 ± 0.059		
	CX3CR1-Cre;GPR35$^{f/f}$ + KA	0.226 ± 0.019		
		**DF**	***t***	**p**
p-AKT	*GPR35$^{f/f}$* + PBS *vs. GPR35$^{f/f}$* + KA	18	3.374	0.0201
	GPR35$^{f/f}$ + PBS *vs. CX3CR1-Cre;GPR35$^{f/f}$* + KA	18	5.573	0.0002
	GPR35$^{f/f}$ + KA *vs. CX3CR1-Cre;GPR35$^{f/f}$* + KA	18	2.200	0.2228
p-PI3K	*GPR35$^{f/f}$* + PBS *vs. GPR35$^{f/f}$* + KA	18	3.832	0.0073
	GPR35$^{f/f}$+PBS *vs. CX3CR1-Cre;GPR35$^{f/f}$*+KA	18	10.44	<0.0001
	GPR35$^{f/f}$+KA *vs. CX3CR1-Cre;GPR35$^{f/f}$*+KA	18	6.607	<0.0001

Table S40. AKT and PI3K protein in hippocampus from KA-induced *CX3CR1-Cre;GPR35$^{f/f}$* and KA-induced *GPR35$^{f/f}$* mice. Numeric data on Figure 7G.

Figure 7G		Relative expression		
AKT	*GPR35$^{f/f}$* + PBS	0.911 ± 0.064		
	GPR35$^{f/f}$ + KA	0.792 ± 0.036		
	CX3CR1-Cre;GPR35$^{f/f}$ + KA	0.995 ± 0.027		
PI3K	*GPR35$^{f/f}$* + PBS	0.930 ± 0.051		
	GPR35$^{f/f}$ + KA	0.767 ± 0.017		
	CX3CR1-Cre;GPR35$^{f/f}$ + KA	0.768 ± 0.080		
		DF	*t*	p
AKT	*GPR35$^{f/f}$* + PBS *vs.* *GPR35$^{f/f}$* + KA	18	1.661	0.5163
	GPR35$^{f/f}$ + PBS *vs.* *CX3CR1-Cre;GPR35$^{f/f}$* + KA	18	1.171	0.8314
	GPR35$^{f/f}$ + KA *vs.* *CX3CR1-Cre;GPR35$^{f/f}$* + KA	18	2.833	0.0644
PI3K	*GPR35$^{f/f}$* + PBS *vs.* *GPR35$^{f/f}$* + KA	18	2.270	0.1963
	GPR35$^{f/f}$ + PBS *vs.* *CX3CR1-Cre;GPR35$^{f/f}$* + KA	18	2.258	0.2005
	GPR35$^{f/f}$ + KA *vs.* *CX3CR1-Cre;GPR35$^{f/f}$* + KA	18	0.01170	>0.9999

Table S41. TNF-α protein in Glu-BV2 cells treated with LV-shRNA-GPR35 or control. Numeric data on Figure 7H.

Figure 7H	TNF-α (ng/mL)		
Ctrl	65.82 ± 0.21		
Glu	122.34 ± 0.31		
LV-Scr1 + Glu	120.87 ± 4.99		
LV-shGPR35 + Glu	150.64 ± 7.24		
	DF	*t*	p
Ctrl *vs.* Glu	12	9.086	<0.0001
Ctrl *vs.* LV-Scr1 + Glu	12	8.849	<0.0001
Ctrl *vs.* LV-shGPR35 + Glu	12	13.63	<0.0001
Glu *vs.* LV-Scr1 + Glu	12	0.2371	>0.9999
Glu *vs.* LV-shGPR35 + Glu	12	4.548	0.0040
LV-Scr1 + Glu *vs.* LV-shGPR35 + Glu	12	4.785	0.0027

Table S42. IL-1β protein in Glu-BV2 cells treated with LV-shRNA-GPR35 or control. Numeric data on Figure 7I.

Figure 7I	IL-1β (ng/mL)
Ctrl	115.00 ± 3.84
Glu	153.86 ± 3.19
LV-Scr1 + Glu	163.51 ± 4.55
LV-shGPR35 + Glu	185.27 ± 4.51

	DF	*t*	p
**Ctrl *vs.* Glu**	16	6.762	<0.0001
**Ctrl *vs.* LV-Scr1 + Glu**	16	8.442	<0.0001
**Ctrl *vs.* LV-shGPR35 + Glu**	16	12.23	<0.0001
**Glu *vs.* LV-Scr1 + Glu**	16	1.679	0.5114
**Glu *vs.* LV-shGPR35 + Glu**	16	5.467	0.0003
**LV-Scr1 + Glu *vs.* LV-shGPR35 + Glu**	16	3.788	0.0096

Table S43. IL-6 protein in Glu-BV2 cells treated with LV-shRNA-GPR35 or control. Numeric data on Figure 7J.

Figure 7J	IL-6 (ng/mL)		
**Ctrl**	63.87 ± 4.90		
**Glu**	145.83 ± 7.95		
**LV-Scr1 + Glu**	132.69 ± 2.89		
**LV-shGPR35 + Glu**	216.62 ± 10.59		
	**DF**	***t***	**p**
**Ctrl *vs.* Glu**	12	8.043	<0.0001
**Ctrl *vs.* LV-Scr1 + Glu**	12	6.754	0.0001
**Ctrl *vs.* LV-shGPR35 + Glu**	12	14.99	<0.0001
**Glu *vs.* LV-Scr1 + Glu**	12	1.289	0.7779
**Glu *vs.* LV-shGPR35 + Glu**	12	6.948	<0.0001
**LV-Scr1+Glu *vs.* LV-shGPR35+Glu**	12	8.237	<0.0001

Table S44. IL-10 protein in Glu-BV2 cells treated with LV-shRNA-GPR35 or control. Numeric data on Figure 7K.

Figure 7K	IL-10 (ng/mL)		
**Ctrl**	65.44 ± 1.57		
**Glu**	178.70 ± 5.09		
**LV-Scr1 + Glu**	165.94 ± 1.69		
**LV-shGPR35 + Glu**	104.14 ± 3.60		
	**DF**	***t***	**p**
**Ctrl *vs.* Glu**	12	24.10	<0.0001
**Ctrl *vs.* LV-Scr1 + Glu**	12	21.38	<0.0001
**Ctrl *vs.* LV-shGPR35 + Glu**	12	8.233	<0.0001
**Glu *vs.* LV-Scr1 + Glu**	12	2.715	0.1075
**Glu *vs.* LV-shGPR35 + Glu**	12	15.86	<0.0001
**LV-Scr1 + Glu *vs.* LV-shGPR35 + Glu**	12	13.15	<0.0001

Table S45. TNF-α protein in Glu-BV2 cells treated with LV-GPR35 or control. Numeric data on Figure 7L.

Figure 7L	TNF-α (ng/mL)		
Ctrl	69.40 ± 3.46		
Glu	125.03 ± 3.10		
LV-Scr2 + Glu	126.06 ± 2.05		
LV-GPR35 + Glu	83.85 ± 6.50		
	DF	*t*	p
Ctrl *vs.* Glu	12	9.541	<0.0001
Ctrl *vs.* LV-Scr2 + Glu	12	9.719	<0.0001
Ctrl *vs.* LV-GPR35 + Glu	12	2.478	0.1623
Glu *vs.* LV-Scr2 + Glu	12	0.1774	>0.9999
Glu *vs.* LV-GPR35 + Glu	12	7.064	<0.0001
LV-Scr2 + Glu *vs.* LV-GPR35 + Glu	12	7.241	<0.0001

Table S46. IL-1β protein in Glu-BV2 cells treated with LV-GPR35 or control. Numeric data on Figure 7M.

Figure 7M	IL-1β (ng/mL)		
Ctrl	126.19 ± 0.36		
Glu	180.54 ± 8.86		
LV-Scr2 + Glu	185.56 ± 8.36		
LV-GPR35 + Glu	144.75 ± 8.79		
	DF	*t*	p
Ctrl *vs.* Glu	12	5.116	0.0015
Ctrl *vs.* LV-Scr2 + Glu	12	5.588	0.0007
Ctrl *vs.* LV-GPR35 + Glu	12	1.747	0.4903
Glu *vs.* LV-Scr2 + Glu	12	0.472	0.9980
Glu *vs.* LV-GPR35 + Glu	12	3.369	0.0330
LV-Scr2 + Glu *vs.* LV-GPR35 + Glu	12	3.841	0.0140

Table S47. IL-6 protein in Glu-BV2 cells treated with LV-GPR35 or control. Numeric data on Figure 7N.

Figure 7N	IL-6 (ng/mL)		
Ctrl	62.00 ± 5.74		
Glu	161.65 ± 0.33		
LV-Scr2 + Glu	165.06 ± 0.44		
LV-GPR35 + Glu	83.13 ± 3.57		
	DF	*t*	p
Ctrl *vs.* Glu	12	20.78	<0.0001
Ctrl *vs.* LV-Scr2 + Glu	12	21.49	<0.0001

**Ctrl *vs.* LV-GPR35 + Glu**	12	4.406	0.0051
**Glu *vs.* LV-Scr2 + Glu**	12	0.7101	0.9827
**Glu *vs.* LV-GPR35 + Glu**	12	16.38	<0.0001
**LV-Scr2 + Glu *vs.* LV-GPR35 + Glu**	12	17.09	<0.0001

Table S48. IL-10 protein in Glu-BV2 cells treated with LV-GPR35 or control. Numeric data on Figure 7O.

Figure 7O	IL-10 (ng/mL)		
**Ctrl**	75.51 ± 5.55		
**Glu**	121.37 ± 8.16		
**LV-Scr2 + Glu**	136.47 ± 8.89		
**LV-GPR35 + Glu**	169.47 ± 7.90		
	**DF**	***t***	**p**
**Ctrl *vs.* Glu**	12	4.197	0.0074
**Ctrl *vs.* LV-Scr2 + Glu**	12	5.579	0.0007
**Ctrl *vs.* LV-GPR35 + Glu**	12	8.599	<0.0001
**Glu *vs.* LV-Scr2 + Glu**	12	1.382	0.7219
**Glu *vs.* LV-GPR35 + Glu**	12	4.402	0.0052
**LV-Scr2 + Glu *vs.* LV-GPR35 + Glu**	12	3.020	0.0623

Table S49. Hippocampal GPR35 protein in *CX3CR1-Cre* mice infected with AAV-EGFP-Con or AAV-EGFP-shPDGFA at 4-week time points following KA-induced or sham treatment. Numeric data on Figure 8E.

Figure 8E	GPR35/Actin relative density		
CX3CR1-Cre **KA**	1.086 ± 0.043		
CX3CR1-Cre **+ AAV-Con2 KA**	1.354 ± 0.090		
CX3CR1-Cre **+ PDGFA^{KD} KA**	1.183 ± 0.061		
	**DF**	***t***	**p**
CX3CR1-Cre **KA *vs.* *CX3CR1-Cre* + AAV-Con2 KA**	8	2.541	0.1004
CX3CR1-Cre **KA *vs.* *CX3CR1-Cre* + PDGFA^{KD} KA**	8	0.9182	0.7678
CX3CR1-Cre **+ AAV-Con2 KA *vs.* *CX3CR1-Cre* + PDGFA^{KD} KA**	8	1.753	0.3131

Table S50. Hippocampal PDGFA protein in *CX3CR1-Cre* mice infected with AAV-EGFP-Con or AAV-EGFP-shPDGFA at 4-week time points following KA-induced or sham treatment. Numeric data on Figure 8F.

Figure 8F	PDGFA/Actin relative density
CX3CR1-Cre **KA**	0.906 ± 0.027
CX3CR1-Cre **+ AAV-Con2 KA**	0.822 ± 0.016

CX3CR1-Cre + PDGFA^{KD} KA	0.557 ± 0.052		
	DF	*q*	p
CX3CR1-Cre KA *vs.* *CX3CR1-Cre* + AAV-Con2 KA	9	1.684	0.3334
CX3CR1-Cre KA *vs.* *CX3CR1-Cre* + PDGFA^{KD} KA	9	7.017	0.0002
CX3CR1-Cre + AAV-Con2 KA *vs.* *CX3CR1-Cre* + PDGFA^{KD} KA	9	5.333	0.0014

Table S51. Hippocampal PDGFA protein in *CX3CR1-Cre;GPR35$^{f/f}$* mice infected with AAV-mCherry-Con3 or AAV-mCherry-PDGFA at 4-week time points following KA-induced or sham treatment. Numeric data on Figure 8K.

Figure 8K	PDGFA/Actin relative density		
GPR35$^{f/f}$ + AAV-Con3 KA	0.477 ± 0.064		
CX3CR1-Cre; GPR35$^{f/f}$ + AAV-Con3 KA	0.194 ± 0.021		
GPR35$^{f/f}$ + PDGFA^{OE} KA	0.446 ± 0.041		
CX3CR1-Cre; GPR35$^{f/f}$ + PDGFA^{OE} KA	1.092 ± 0.070		
	DF	*t*	p
GPR35$^{f/f}$ + AAV-Con3 KA *vs.* *CX3CR1-Cre; GPR35$^{f/f}$* + AAV-Con3 KA	12	3.798	0.0151
GPR35$^{f/f}$ + AAV-Con3 KA *vs.* *GPR35$^{f/f}$* + PDGFA^{OE} KA	12	0.4162	0.9990
GPR35$^{f/f}$ + AAV-Con3 KA *vs.* *CX3CR1-Cre; GPR35$^{f/f}$* + PDGFA^{OE} KA	12	8.255	<0.0001
CX3CR1-Cre; GPR35$^{f/f}$ + AAV-Con3 KA *vs.* *GPR35$^{f/f}$* + PDGFA^{OE} KA	12	3.382	0.0323
CX3CR1-Cre; GPR35$^{f/f}$ + AAV-Con3 KA *vs.* *CX3CR1-Cre; GPR35$^{f/f}$* + PDGFA^{OE} KA	12	12.05	<0.0001
GPR35$^{f/f}$ + PDGFA^{OE} KA *vs.* *CX3CR1-Cre; GPR35$^{f/f}$* + PDGFA^{OE} KA	12	8.671	<0.0001

Table S52. Hippocampal GPR35 protein in *CX3CR1-Cre;GPR35$^{f/f}$* mice infected with AAV-mCherry-Con3 or AAV-mCherry-PDGFA at 4-week time points following KA-induced or sham treatment. Numeric data on Figure 8M.

Figure 8M	GPR35/Actin relative density		
GPR35$^{f/f}$ + AAV-Con3 KA	0.577 ± 0.072		
CX3CR1-Cre; GPR35$^{f/f}$ + AAV-Con3 KA	0.238 ± 0.058		
GPR35$^{f/f}$ + PDGFA^{OE} KA	0.679 ± 0.089		
CX3CR1-Cre; GPR35$^{f/f}$ + PDGFA^{OE} KA	0.366 ± 0.067		
	DF	*t*	p
GPR35$^{f/f}$ + AAV-Con3 KA *vs.* *CX3CR1-Cre; GPR35$^{f/f}$* + AAV-Con3 KA	20	3.302	0.0211
GPR35$^{f/f}$ + AAV-Con3 KA *vs.* *GPR35$^{f/f}$* + PDGFA^{OE} KA	20	0.9966	0.9103
GPR35$^{f/f}$ + AAV-Con3 KA *vs.* *CX3CR1-Cre; GPR35$^{f/f}$* +	20	2.060	0.2770

	DF	*t*	p
**PDGFA^{OE} KA**			
CX3CR1-Cre; GPR35$^{f/f}$ **+ AAV-Con3 KA *vs.* *GPR35$^{f/f}$* + PDGFA^{OE} KA**	20	4.299	0.0021
CX3CR1-Cre; GPR35$^{f/f}$ **+ AAV-Con3 KA *vs.* *CX3CR1-Cre; GPR35$^{f/f}$* + PDGFA^{OE} KA**	20	1.242	0.7892
GPR35$^{f/f}$ **+ PDGFA^{OE} KA *vs.* *CX3CR1-Cre; GPR35$^{f/f}$* + PDGFA^{OE} KA**	20	3.057	0.0368

Table S53. Hippocampal p-AKT and p-PI3K protein in *CX3CR1-Cre;GPR35$^{f/f}$* mice infected with AAV-mCherry-Con3 or AAV-mCherry-PDGFA at 4-week time points following KA-induced or sham treatment. Numeric data on Figure 8N.

	Figure 8N	Relative expression		
**p-AKT**	***GPR35$^{f/f}$*** **+ AAV-Con3 KA**	1.123 ± 0.172		
	CX3CR1-Cre; GPR35$^{f/f}$ **+ AAV-Con3 KA**	0.591 ± 0.053		
	GPR35$^{f/f}$ **+ PDGFA^{OE} KA**	1.077 ± 0.109		
	CX3CR1-Cre; GPR35$^{f/f}$ **+ PDGFA^{OE} KA**	1.316 ± 0.123		
**p-PI3K**	***GPR35$^{f/f}$*** **+ AAV-Con3 KA**	1.043 ± 0.161		
	CX3CR1-Cre; GPR35$^{f/f}$ **+ AAV-Con3 KA**	0.525 ± 0.048		
	GPR35$^{f/f}$ **+ PDGFA^{OE} KA**	1.054 ± 0.155		
	CX3CR1-Cre; GPR35$^{f/f}$ **+ PDGFA^{OE} KA**	1.118 ± 0.084		
		**DF**	***t***	**p**
**p-AKT**	***GPR35$^{f/f}$*** **+ AAV-Con3 KA *vs.* *CX3CR1-Cre; GPR35$^{f/f}$* + AAV-Con3 KA**	40	3.090	0.0428
	GPR35$^{f/f}$ **+ AAV-Con3 KA *vs.* *GPR35$^{f/f}$* + PDGFA^{OE} KA**	40	0.2666	>0.9999
	GPR35$^{f/f}$ **+ AAV-Con3 KA *vs.* *CX3CR1-Cre; GPR35$^{f/f}$* + PDGFA^{OE} KA**	40	1.120	0.9769
	CX3CR1-Cre; GPR35$^{f/f}$ **+ AAV-Con3 KA *vs.* *GPR35$^{f/f}$* + PDGFA^{OE} KA**	40	2.823	0.0850
	CX3CR1-Cre; GPR35$^{f/f}$ **+ AAV-Con3 KA *vs.* Group D**	40	4.210	0.0017
	GPR35$^{f/f}$ **+ PDGFA^{OE} KA *vs.* *CX3CR1-Cre; GPR35$^{f/f}$* + PDGFA^{OE} KA**	40	1.386	0.8981
**p-PI3K**	***GPR35$^{f/f}$*** **+ AAV-Con3 KA *vs.* *CX3CR1-Cre; GPR35$^{f/f}$* + AAV-Con3 KA**	40	3.008	0.0530
	GPR35$^{f/f}$ **+ AAV-Con3 KA *vs.* *GPR35$^{f/f}$* + PDGFA^{OE} KA**	40	0.06394	>0.9999
	GPR35$^{f/f}$ **+ AAV-Con3 KA *vs.* *CX3CR1-Cre; GPR35$^{f/f}$* + PDGFA^{OE} KA**	40	0.4377	>0.9999
	***vs.* *GPR35$^{f/f}$* + PDGFA^{OE} KA**	40	3.072	0.0448
	CX3CR1-Cre; GPR35$^{f/f}$ **+ AAV-Con3 KA *vs.* *CX3CR1-Cre; GPR35$^{f/f}$* + PDGFA^{OE} KA**	40	3.446	0.0161
	GPR35$^{f/f}$ **+ PDGFA^{OE} KA *vs.* *CX3CR1-Cre; GPR35$^{f/f}$* + PDGFA^{OE} KA**	40	0.3738	>0.9999

Table S54. Hippocampal AKT and PI3K protein in *CX3CR1-Cre;GPR35$^{f/f}$* mice infected with AAV-mCherry-Con3 or AAV-mCherry-PDGFA at 4-week time points following KA-induced or sham treatment. Numeric data on Figure 8O.

	Figure 8O	Relative expression		
AKT	*GPR35$^{f/f}$* + AAV-Con3 KA	1.759 ± 0.301		
	CX3CR1-Cre; GPR35$^{f/f}$ + AAV-Con3 KA	1.709 ± 0.262		
	GPR35$^{f/f}$ + PDGFA^{OE} KA	1.988 ± 0.358		
	CX3CR1-Cre; GPR35$^{f/f}$ + PDGFA^{OE} KA	2.003 ± 0.371		
PI3K	*GPR35$^{f/f}$* + AAV-Con3 KA	1.214 ± 0.268		
	CX3CR1-Cre; GPR35$^{f/f}$ + AAV-Con3 KA	1.108 ± 0.285		
	GPR35$^{f/f}$ + PDGFA^{OE} KA	1.231 ± 0.290		
	CX3CR1-Cre; GPR35$^{f/f}$ + PDGFA^{OE} KA	1.324 ± 0.323		
		DF	*q*	p
AKT	*GPR35$^{f/f}$* + AAV-Con3 KA *vs.* *CX3CR1-Cre; GPR35$^{f/f}$* + AAV-Con3 KA	32.00	0.1169	>0.9999
	GPR35$^{f/f}$ + AAV-Con3 KA *vs.* *GPR35$^{f/f}$* + PDGFA^{OE} KA	32.00	0.5376	>0.9999
	GPR35$^{f/f}$ + AAV-Con3 KA *vs.* *CX3CR1-Cre; GPR35$^{f/f}$* + PDGFA^{OE} KA	32.00	0.5740	>0.9999
	CX3CR1-Cre; GPR35$^{f/f}$ + AAV-Con3 KA *vs.* *GPR35$^{f/f}$* + PDGFA^{OE} KA	32.00	0.6545	0.9998
	Group B *vs.* Group D	32.00	0.6909	0.9997
	GPR35$^{f/f}$ + PDGFA^{OE} KA *vs.* *CX3CR1-Cre; GPR35$^{f/f}$* + PDGFA^{OE} KA	32.00	0.03643	>0.9999
PI3K	*GPR35$^{f/f}$* + AAV-Con3 KA *vs.* *CX3CR1-Cre; GPR35$^{f/f}$* + AAV-Con3 KA	32.00	0.4395	>0.9999
	GPR35$^{f/f}$ + AAV-Con3 KA *vs.* *GPR35$^{f/f}$* + PDGFA^{OE} KA	32.00	0.08844	>0.9999
	GPR35$^{f/f}$ + AAV-Con3 KA *vs.* *CX3CR1-Cre; GPR35$^{f/f}$* + PDGFA^{OE} KA	32.00	0.4523	>0.9999
	CX3CR1-Cre; GPR35$^{f/f}$ + AAV-Con3 KA *vs.* *GPR35$^{f/f}$* + PDGFA^{OE} KA	32.00	0.3510	>0.9999
	CX3CR1-Cre; GPR35$^{f/f}$ + AAV-Con3 KA *vs.* Group D	32.00	0.8918	0.9967
	GPR35$^{f/f}$ + PDGFA^{OE} KA *vs.* *CX3CR1-Cre; GPR35$^{f/f}$* + PDGFA^{OE} KA	32.00	0.5407	>0.9999

Table S55. Electrographic seizure-like activities assessed at the chronic stage in *GPR35*$^{f/f}$ KA + PBS, *GPR35*$^{f/f}$ KA + L-Kyna and *CX3CR1-Cre;GPR35*$^{f/f}$ KA + L-Kyna mice. Numeric data on Figure 9C.

Figure 9C	Group	Data		
No. of seizures per day	*GPR35*$^{f/f}$ KA + PBS	1.000 ± 0.430		
	GPR35$^{f/f}$ KA + L-Kyna	0.667 ± 0.413		
	CX3CR1-Cre; GPR35$^{f/f}$ KA + L-Kyna	4.000 ± 0.365		
Seizure duration (sec)	*GPR35*$^{f/f}$ KA + PBS	89.9 ± 21.5		
	GPR35$^{f/f}$ KA + L-Kyna	22.4 ± 10.3		
	CX3CR1-Cre; GPR35$^{f/f}$ KA + L-Kyna	76.0 ± 20.7		
		**DF**	***t***	**p**
No. of seizures per day	*GPR35*$^{f/f}$ KA + PBS *vs.* *GPR35*$^{f/f}$ KA + L-Kyna	14	0.5916	0.9169
	GPR35$^{f/f}$ KA + PBS *vs.* *CX3CR1-Cre; GPR35*$^{f/f}$ KA + L-Kyna	14	5.077	0.0005
	GPR35$^{f/f}$ KA + L-Kyna *vs.* *CX3CR1-Cre; GPR35*$^{f/f}$ KA + L-Kyna	14	5.641	0.0002
Seizure duration (sec)	*GPR35*$^{f/f}$ KA + PBS *vs.* *GPR35*$^{f/f}$ KA + L-Kyna	14	2.735	0.0475
	GPR35$^{f/f}$ KA + PBS *vs.* *CX3CR1-Cre; GPR35*$^{f/f}$ KA + L-Kyna	14	0.5369	0.9359
	GPR35$^{f/f}$ KA + L-Kyna *vs.* *CX3CR1-Cre; GPR35*$^{f/f}$ KA + L-Kyna	14	2.071	0.1622

Table S56. Open field in WT KA mice treated with PBS or L-Kyna. Numeric data on Figure 9F.

Figure 9F	Group	Data	DF	*t*	p
Time in center(%)	WT KA + PBS	7.77 ± 0.84	-	-	-
	WT KA + L-Kyna	12.75 ± 2.20	18	2.114	0.0487
Total distance(unit)	WT KA + PBS	20.57 ± 2.43	-	-	-
	WT KA + L-Kyna	23.17 ± 1.91	18	0.8373	0.4134

Table S57. Novel object recognition in WT KA mice treated with PBS or L-Kyna. Numeric data on Figure 9G.

Figure 9G	Group	Data	DF	*t*	p
Novel object recognition idex	WT KA + PBS	0.50 ± 0.04	-	-	-
	WT KA + L-Kyna	0.67 ± 0.05	14	2.350	0.0340
Entries in new zone	WT KA + PBS	6.38 ± 0.98	-	-	-
	WT KA + L-Kyna	9.50 ± 0.63	14	2.685	0.0178

Table S58. Morris water maze (latency to find the hidden platform) in WT KA mice treated with PBS or L-Kyna. Numeric data on Figure 9I.

Figure 9I	Group	Data	DF	*t*	p
1d	WT KA + L-Kyna	56.22 ± 3.78	-	-	-
	WT KA + PBS	58.76 ± 1.24	9	1.000	0.8780
2d	WT KA + L-Kyna	12.94 ± 4.20	-	-	-
	WT KA + PBS	11.59 ± 5.42	9	0.1907	>0.9999
3d	WT KA + L-Kyna	25.18 ± 6.83	-	-	-
	WT KA + PBS	33.83 ± 7.37	9	1.080	0.8417
4d	WT KA + L-Kyna	29.22 ± 4.86	-	-	-
	WT KA + PBS	48.90 ± 5.34	9	3.188	0.0540
5d	WT KA + L-Kyna	13.82 ± 1.54	-	-	-
	WT KA + PBS	26.52 ± 4.41	9	3.409	0.0382

Table S59. Morris water maze (platform crossings) in WT KA mice treated with PBS or L-Kyna. Numeric data on Figure 9K.

Figure 9K	Number of crossing	DF	*t*	p
WT KA + PBS	1.40 ± 0.27	-	-	-
WT KA + L-Kyna	2.90 ± 0.60	18	2.270	0.0357

Table S60. Morris water maze (time spent in the target quadrant) in WT KA mice treated with PBS or L-Kyna. Numeric data on Figure 9L.

Figure 9L	Duration in goal quadrant (%)	DF	*t*	p
WT KA + PBS	14.90 ± 1.85	-	-	-
WT KA + L-Kyna	23.53 ± 1.84	18	2.550	0.0201

Supplemental Figures and Figure Legends

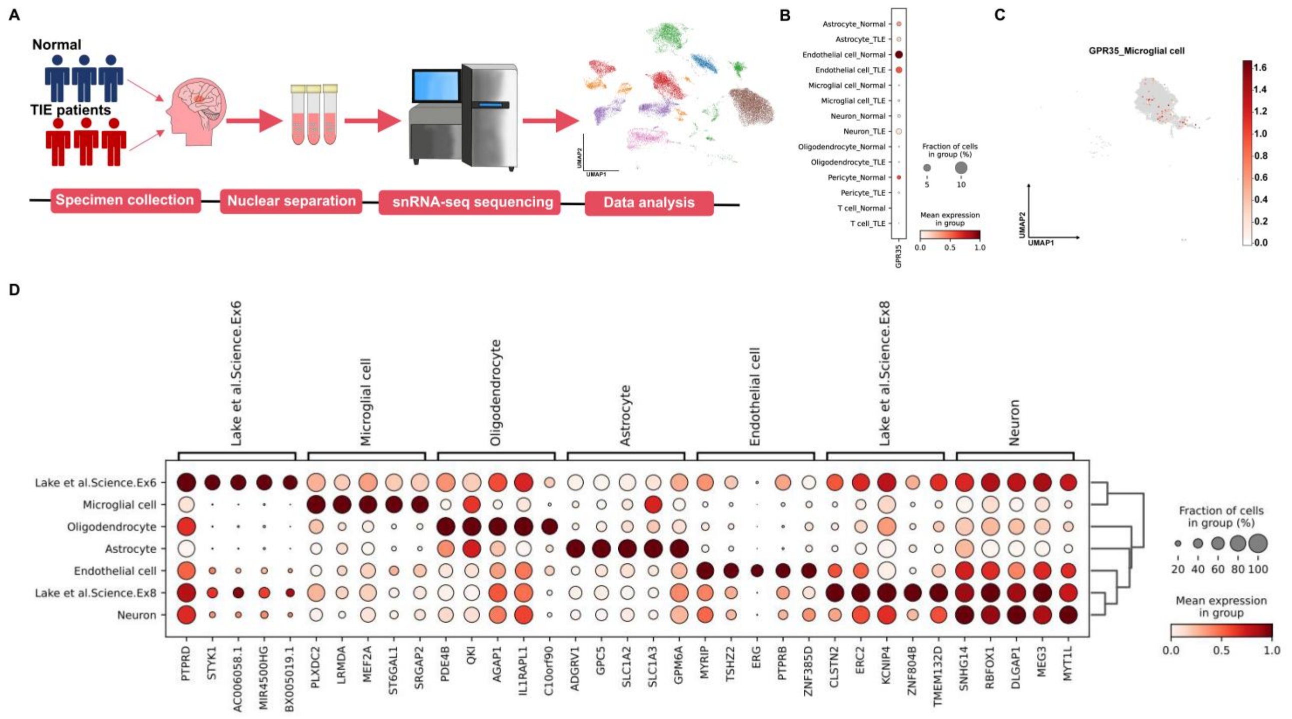

Supplementary Figure S1. Single-nucleus RNA sequencing reveals GPR35 upregulation in microglia from patients with temporal lobe epilepsy (TLE). A) Schematic representation of the single-nucleus RNA sequencing (snRNA-seq) analysis performed on tissue samples from patients with TLE and non-epileptic controls (GSE140393 and GSE190452). B) Dot plot depicting GPR35 expression across major cell populations. C) GPR35 expression in microglias. D) Dot size corresponds to the percentage of nuclei expressing the gene in each cluster, and color represents the average gene expression level.

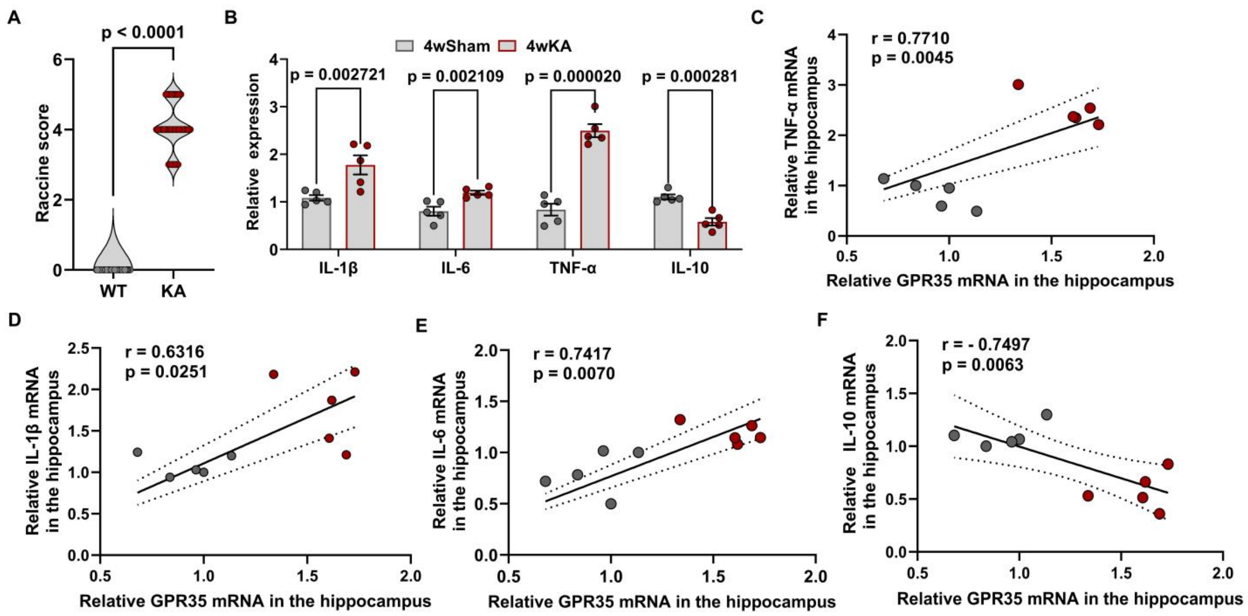

Supplementary Figure S2. Epileptic Model in Mice. Related to Figure 1. A) Behavioral seizures were evaluated at the acute stage using the Racine scale (Sham, n = 10; KA, n = 10). Student's *t* test was used for analysis. B) qRT-PCR analysis of inflammatory cytokines expression (IL-1β, IL-6, TNF-α, IL-10) in hippocampal tissue of KA-induced mice or Sham controls (n = 4). Two-way analysis of covariance (ANCOVA) was used for analysis. C-F) Partial correlation analysis of TNF-α (C), IL-1β (D), IL-6 (E), IL-10 (F) and (H) protein expression and GPR35 expression in hippocampal tissue samples from KA-induced mice (n = 10) compared to Sham controls (n = 10) at 4 weeks. Pearson's correlation coefficient was used for analysis. Data are presented as mean ± standard error of the mean (SEM).

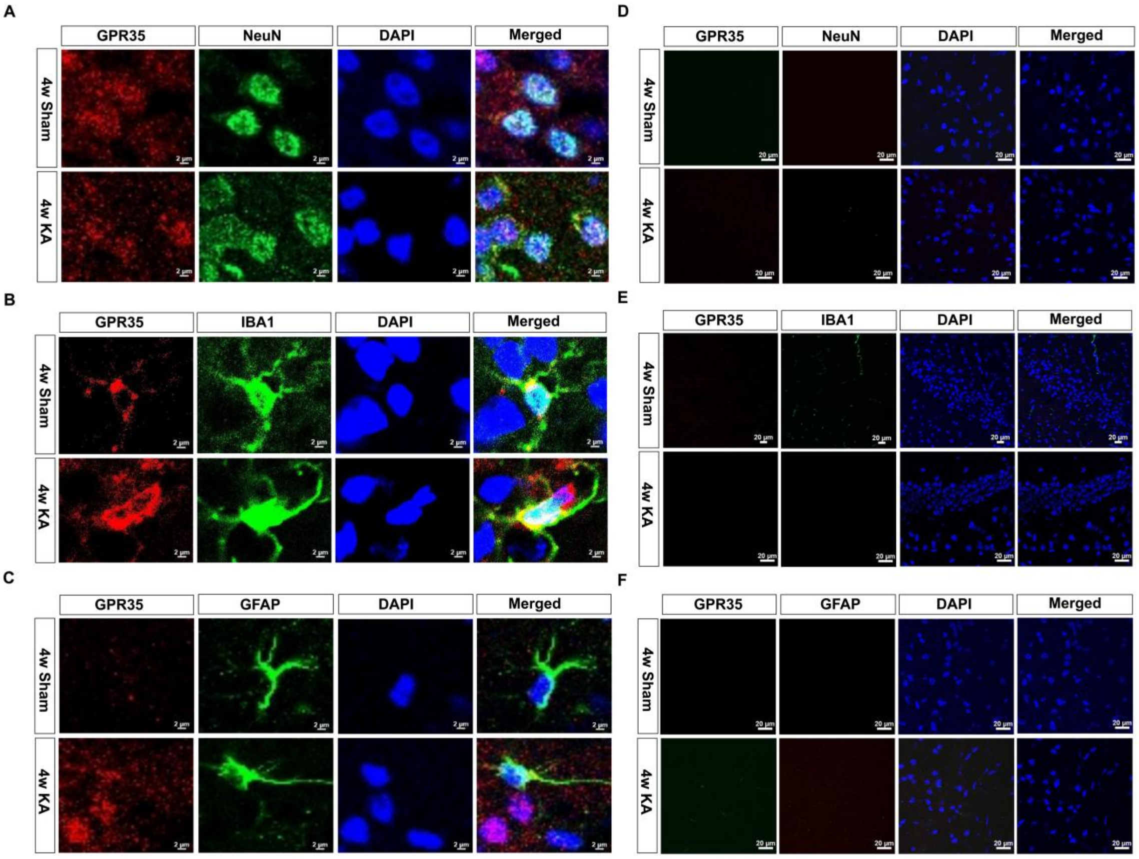

Supplementary Figure S3. Co-localization of GPR35 with neurons in the KA-induced epilepsy model. Representative images of immunofluorescence staining showing the expression of GPR35 expression in neurons (A), microglia (B), and astrocytes (C) after 4-week Sham and 4-week KA treatment. Scale bar: 2 μm. Representative images of immunofluorescence showed background staining of GPR35, NeuN (D), Iba1 (E), and GFAP (F), including regions of signal overlap. Brain tissue samples were obtained from the hippocampus (n = 3). Scale bar: 20 μm.

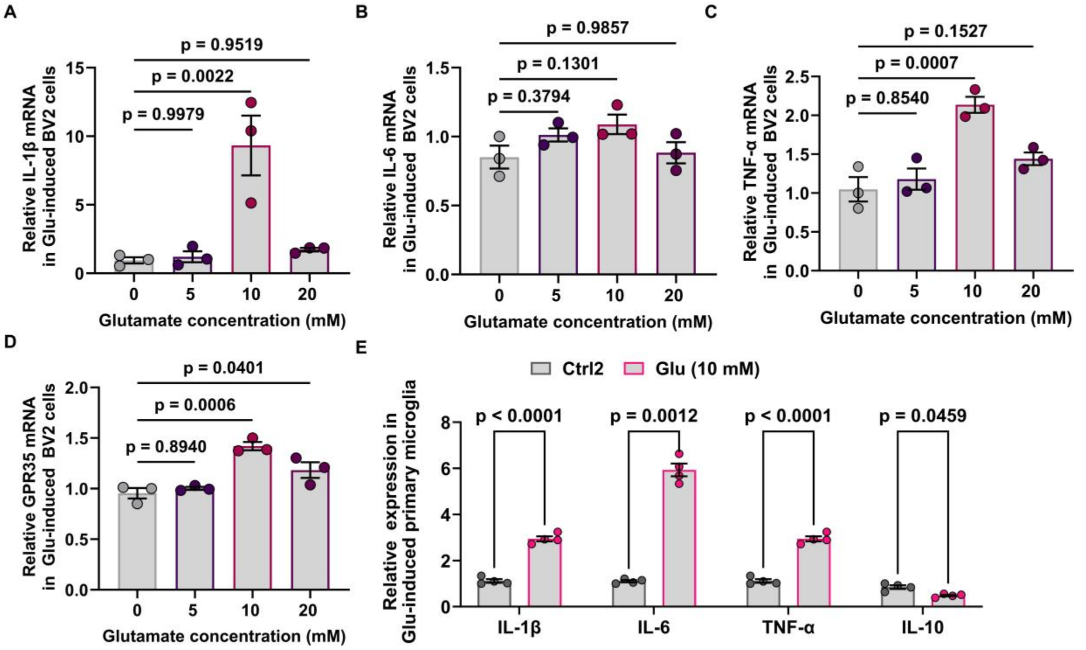

Supplementary Figure S4. *In vivo* Model in cells. Related to Figure 1. A-D) qRT-PCR analysis of inflammatory cytokines expression of IL-1β (A), IL-6 (B), TNF-α (C), and GPR35 (D) in BV2 cells treated with glutamate (5 mM, 10 mM, 20 mM) or vehicle control (n = 3). One-way analysis of covariance (ANCOVA) was used for analysis. E) qRT-PCR analysis of inflammatory cytokines expression of IL-1β, IL-6, TNF-α, and IL-10 in primary microglia treated with glutamate (10 mM) or vehicle control, n = 4). Two-way analysis of covariance (ANCOVA) was used for analysis. Data are presented as mean ± standard error of the mean (SEM)..

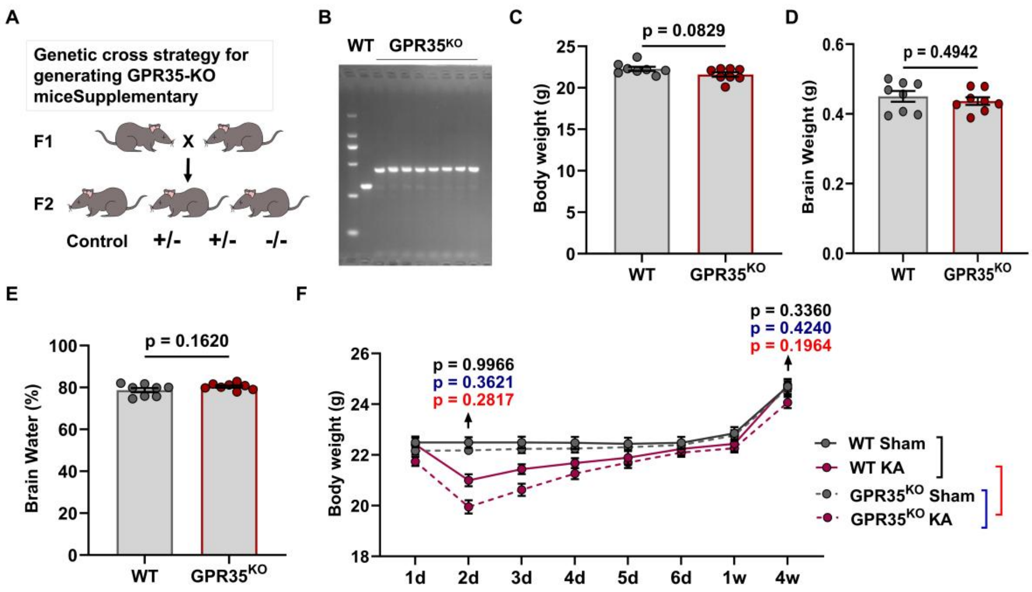

Supplementary Figure S5. Construction of GPR35^{KO} mice. Related to Figure 2. A) Schematic illustration of the genetic crossing strategy for generating GPR35 knockout mice. B) Agarose gel electrophoresis was performed to confirm the efficacy of the GPR35 gene knockout strategy. Distinct DNA bands corresponding to expected fragment sizes were resolved in lanes containing different experimental samples. C) The effect of GPR35 knockout mice on body weight (n = 10). D) Brain weight of GPR35 knockout mice (n = 10). E) Percentage of brain water content of knockout of GPR35 mice (n = 10). One-way analysis of covariance (ANCOVA) was used for analysis. F) GPR35 knockout in mice did not affect body weight (n = 10). Two-way ANCOVA was used for analysis. Dataare presented as mean ± standard error of the mean (SEM).

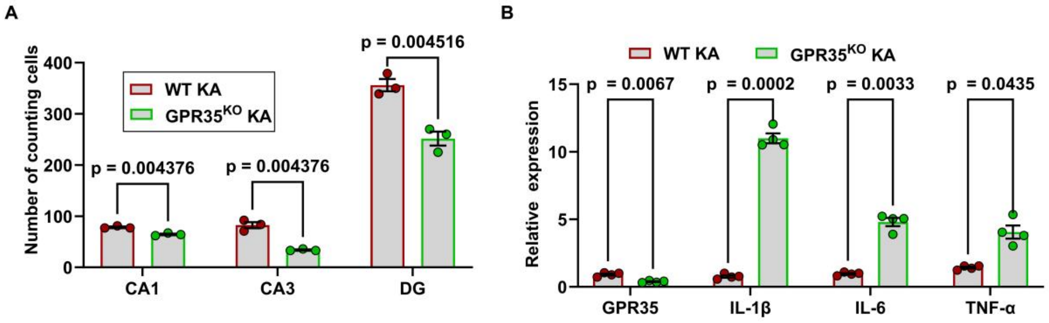

Supplementary Figure S6. Neurodegeneration and GPR35 mRNA and inflammatory cytokine mRNA Expression in GPR35^{KO} mice. Related to Figure 2. A) Nissl staining reveals altered neuronal morphology in the hippocampus of GPR35^{KO} mice, with a significant

reduction in neuronal density compared with wild-type (WT) mice (n = 3). Two-tailed unpaired *t* test was used for analysis. B) qRT-PCR analysis of inflammatory cytokines expression of GPR35, IL-1β, IL-6, and TNF-α in $GPR35^{KO}$ mice or WT mice. (n = 4). Two-way analysis of covariance (ANCOVA) was used for analysis. Data are presented as mean ± standard error of the mean (SEM).

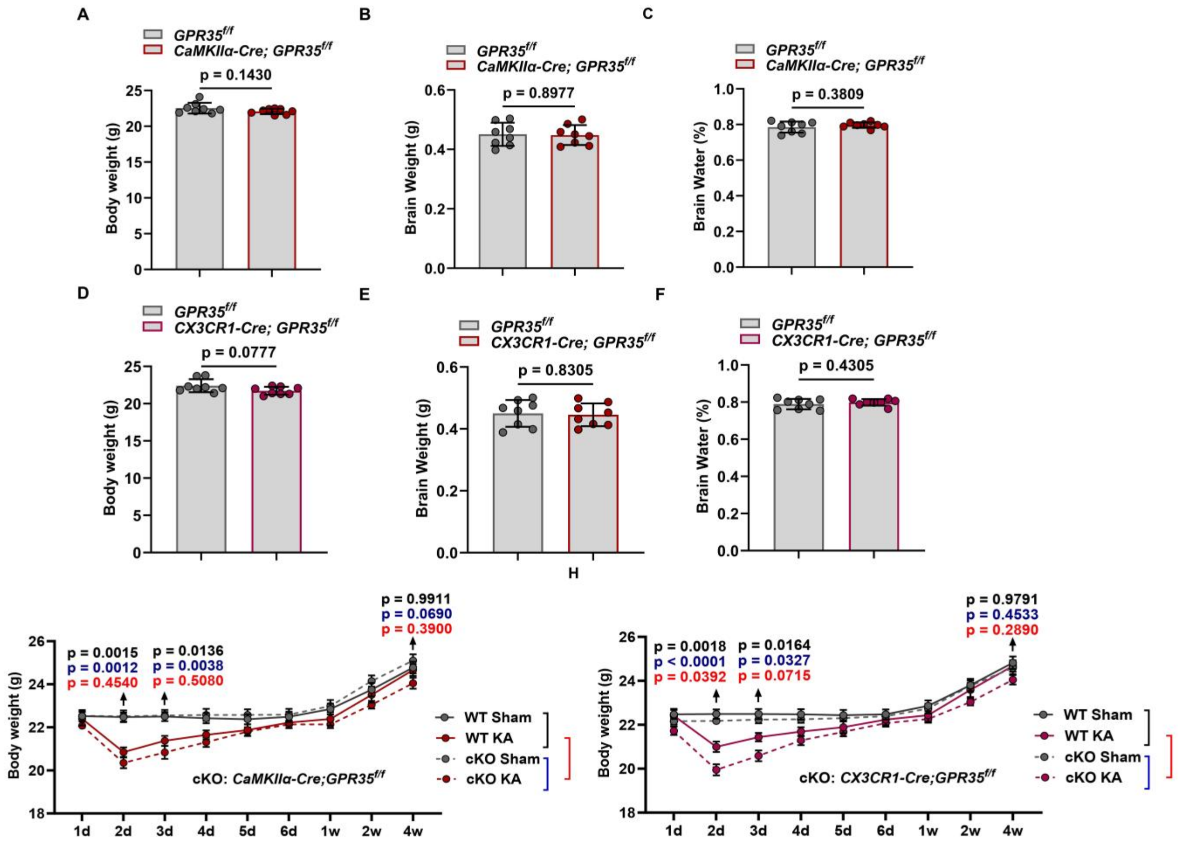

Supplementary Figure S7. Conditional deletion of GPR35 had no effect on development. A and D) The effect of neuron-specific knockout of GPR35 (A) and microglia-specific knockout of GPR35 (D) in mice on body weight (n = 10). B and E) Brain weight of $GPR35^{f/f}$ and $CaMKII\alpha\text{-}Cre;GPR35^{f/f}$ (B) or $CX3CR1\text{-}Cre;GPR35^{f/f}$ (E) mice (n = 10). C and F) Brain water content of $GPR35^{f/f}$ and $CaMKII\alpha\text{-}Cre;GPR35^{f/f}$ (C) or $CX3CR1\text{-}Cre;GPR35^{f/f}$ (F) mice (n = 10). Two-tailed unpaired *t* test was used for analysis. G-H) Neuron-specific knockout of GPR35 (G) and microglia-specific knockout of GPR35 (H) in mice did not affect body weight. Two-way analysis of covariance (ANCOVA) was used for analysis. Data are presented as mean ± standard error of the mean (SEM).

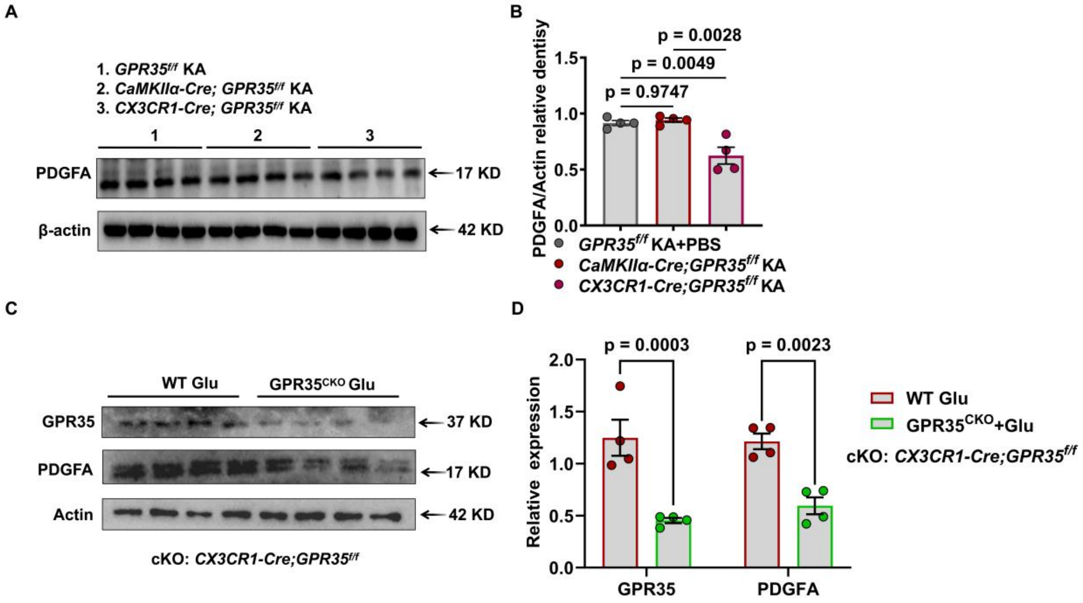

Supplementary Figure S8. PDGFA protein expression in different cell types. A-B) Representative Western blot of PDGFA (A) and quantification of PDGFA expression (B) in the hippocampus in KA-induced *GPR35$^{f/f}$*, *CaMKIIα-Cre;GPR35$^{f/f}$* and *CX3CR1-Cre;GPR35$^{f/f}$* mice (n = 4). One-way analysis of covariance (ANCOVA) was used for analysis. C-D) Representative Western blot of PDGFA (C) and quantification of PDGFA expression (D) in primary microglia were treated with glutamate (n = 4). One-way analysis of covariance (ANCOVA) was used for analysis. Data are presented as mean ± standard error of the mean (SEM).

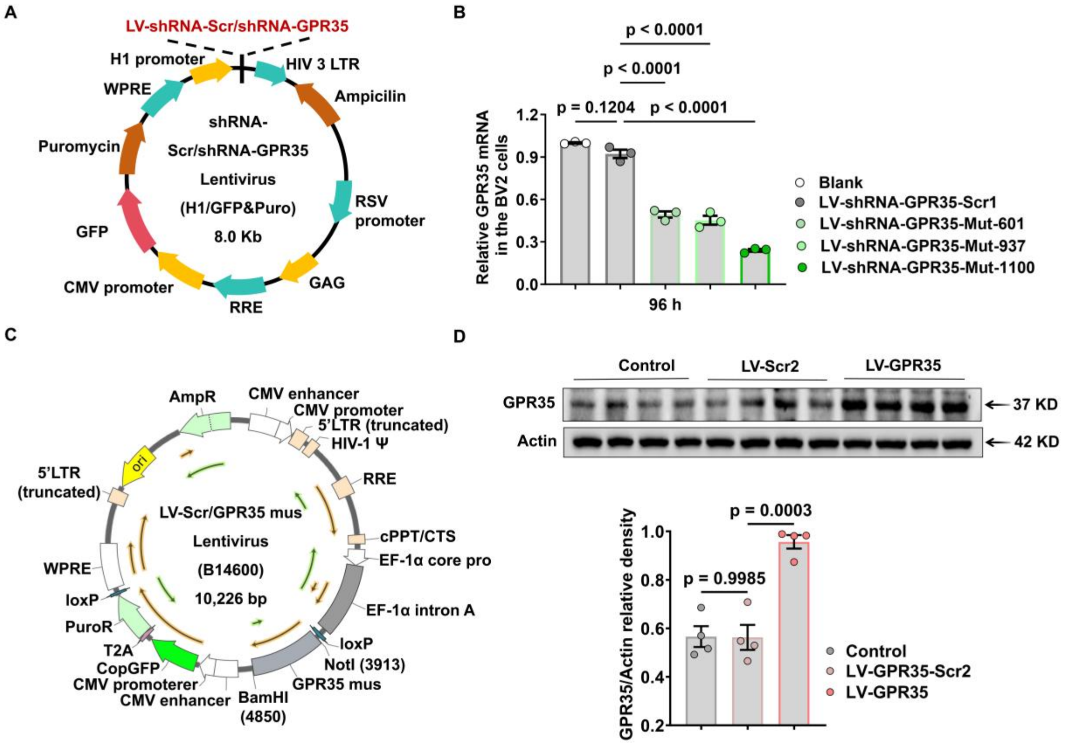

Supplementary Figure S9. Regarding GPR35 lentiviral validation. Related to Figure 4. A) Schematic showing the lentivirus vector encoding LV-GPR35 for GPR35 knockdown. B) LV3-Scr represents shRNA-Scr group, LV-shRNA-GPR35-601 represents shRNA-GPR35 sequence 1, LV-shRNA-GPR35-937 represents shRNA-GPR35 sequence 2, and LV-shRNA-GPR35-1100 represents shRNA-GPR35 sequence 3. There were 3 replicates for each gene in each sample, and the precision met the requirements of further analysis (n = 3). One-way analysis of covariance (ANCOVA) was used for analysis. C) Schematic showing the lentivirus vector encoding GPR35 LV-GPR35 over-expression. D) Western blot analysis of GPR35 exprssion in BV2 cells transfected with LV-GPR35 overexpression (n = 4). One-way ANCOVA was used for analysis. Data are presented as mean ± standard error of the mean (SEM).

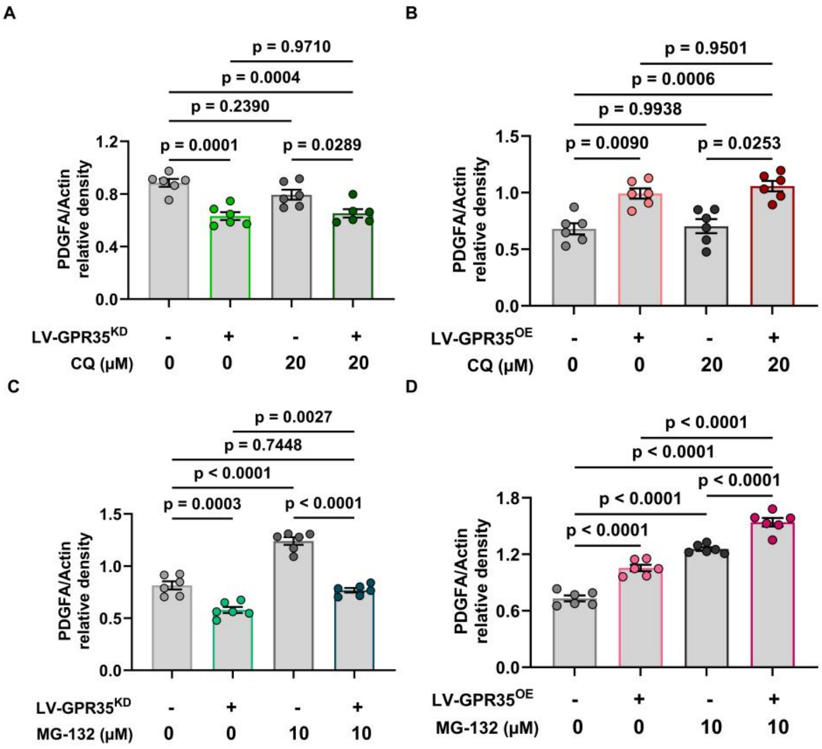

Supplementary Figure S10. Related to Figure 6F-I. Statistical analysis of PDGFA expression in cells transfected with LV-shGPR35 or LV-GPR35. A-B) Cells transfected with LV-shGPR35 or LV-GPR35 were treated with the autophagy/lysosome inhibitor chloroquine (CQ, 20 μM) for 24 hours. Statistical analysis was performed using one-way analysis of covariance (ANCOVA) (n = 6). C-D) Cells transfected with LV-shGPR35 or LV-GPR35 overexpression lentivirus were treated with MG132 (10 μM, proteasome inhibitor) for 24 hours. One-way ANCOVA was used for analysis. Data are presented as mean ± standard error of the mean (SEM).

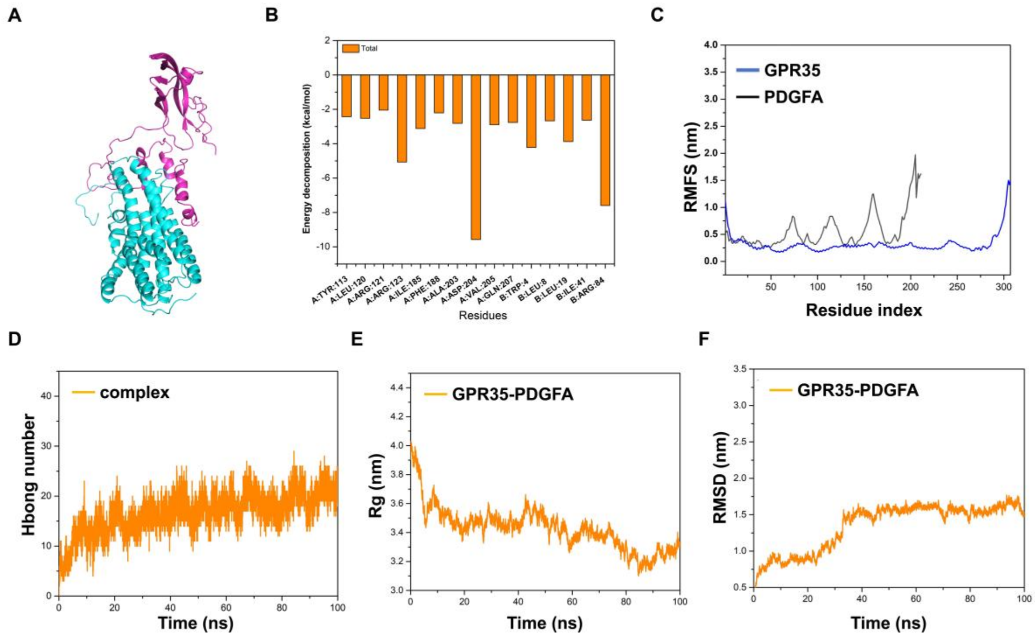

Supplementary Figure S11. The binding mode of the complex PDGFA with GPR35 after 100 ns MD simulation. A) The backbone of protein was rendered in tube and colored in blue (PDGFA) and pink (GPR35). B) The energy decomposition for the interaction between protein. C) RMSF plot during molecular dynamics simulations for protein complex. D) The hydrogen bond number between protein and protein. E) The Rg changes of protein complex during the molecular dynamics. F) RMSD plot during molecular dynamics simulations for the protein complex.

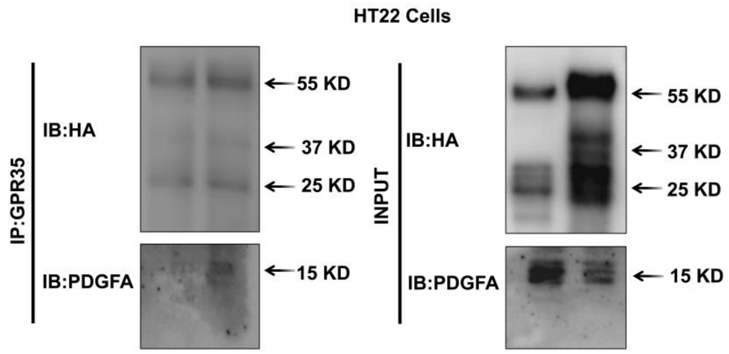

Supplementary Figure S12. Co-immunoprecipitation confirms that GPR35 directly binds to PDGFA. HT22 cells were transfected with HA-GPR35 and HA-PDGFA and subjected to anti-HA immunoprecipitation.

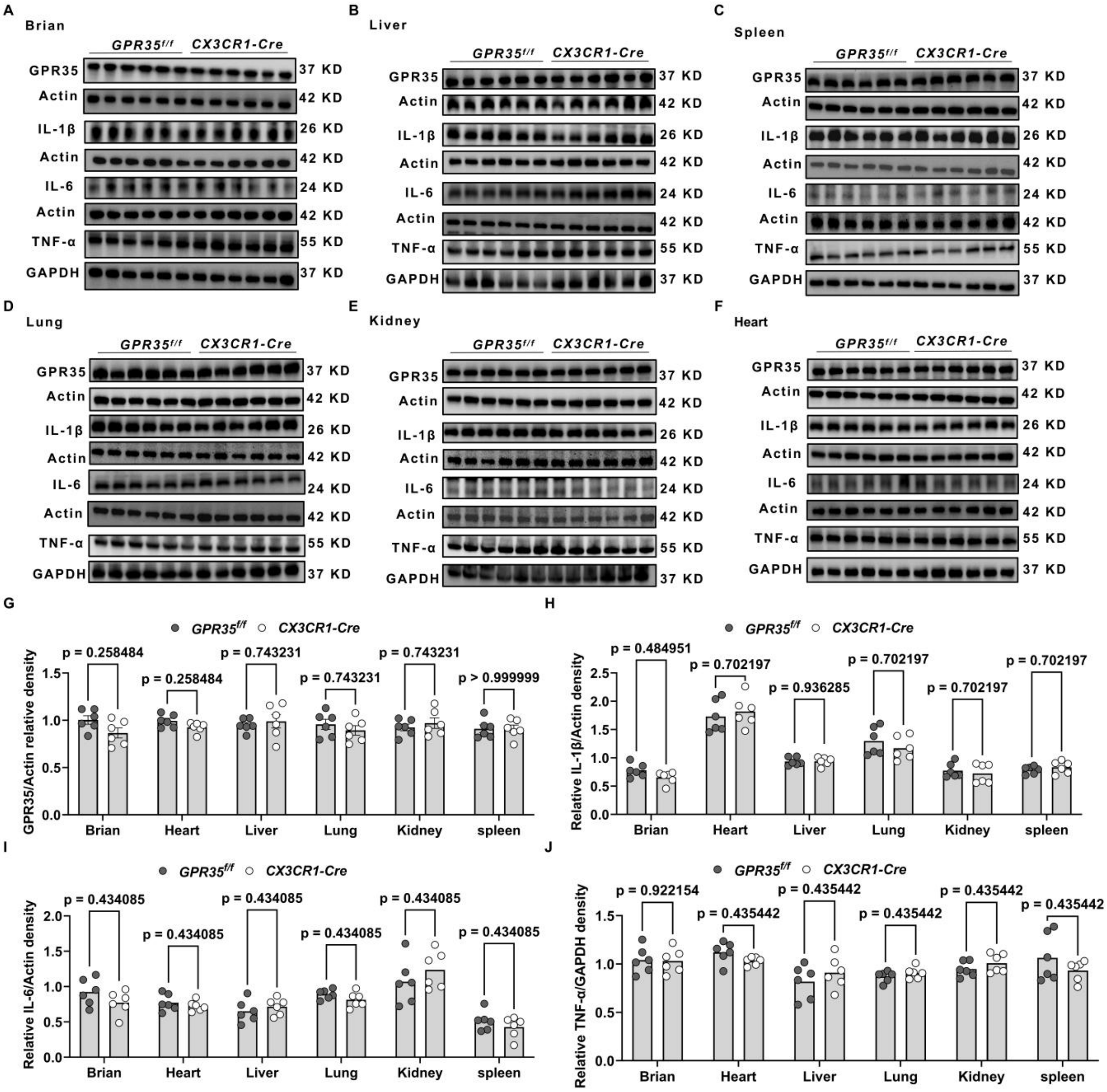

Supplementary Figure S13. Confirmation of Cre expression effects on GPR35 and basal inflammatory protein levels. Western blot analysis of GPR35 and inflammatory proteins (IL-1β, IL-6, TNF-α) in various tissues from *GPR35$^{f/f}$* and *CX3CR1-Cre* mice. (A-F) Representative Western blot (A-F) and quantification (G-J) of hippocampal GPR35 protein and inflammatory proteins (IL-1β, IL-6, TNF-α) protein levels in various tissues, including brain (A), liver (B), spleen (C), lung (D), kidney (E), and heart (F), from *GPR35$^{f/f}$* and *CX3CR1-Cre* mice. One-way analysis of covariance (ANCOVA) was used for analysis. Data are presented as mean ± standard error of the mean (SEM).

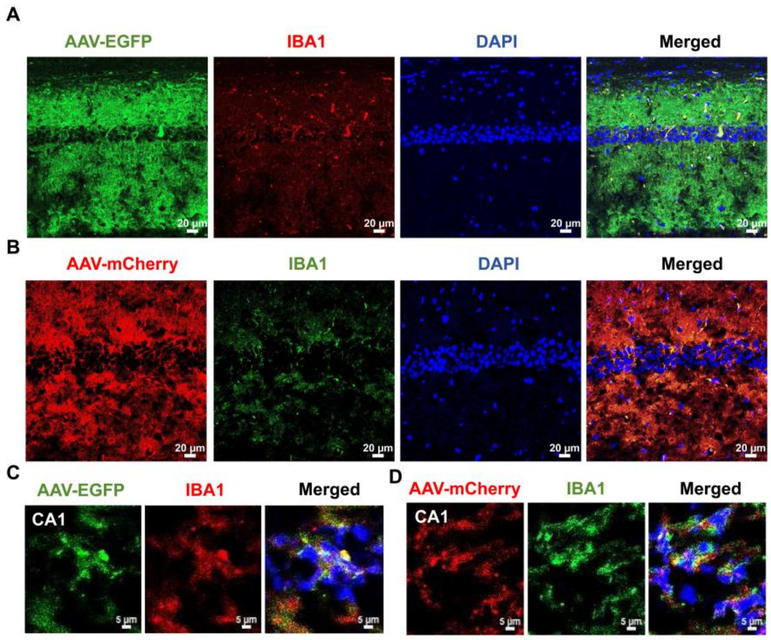

Supplementary Figure S14. Immunocytochemical analysis of IBA1 and AAV-mediated expression in the hippocampus of *CX3CR1-Cre* and *CX3CR1-Cre;GPR35*$^{f/f}$ mice. (A and C) Localization of IBA1 (red) and AAV-EGFP-shPDGFA (green) in hippocampal tissue from *CX3CR1-Cre* mice infected with AAV-EGFP-shPDGFA (n = 3). (B and D) Localization of IBA1 (green) and AAV-mCherry-PDGFA (red) in hippocampal tissue from *CX3CR1-Cre;GPR35*$^{f/f}$ mice infected with AAV-mCherry-PDGFA (n = 3). Scale bars: 20 µm (A, B) and 5 µm (C, D).

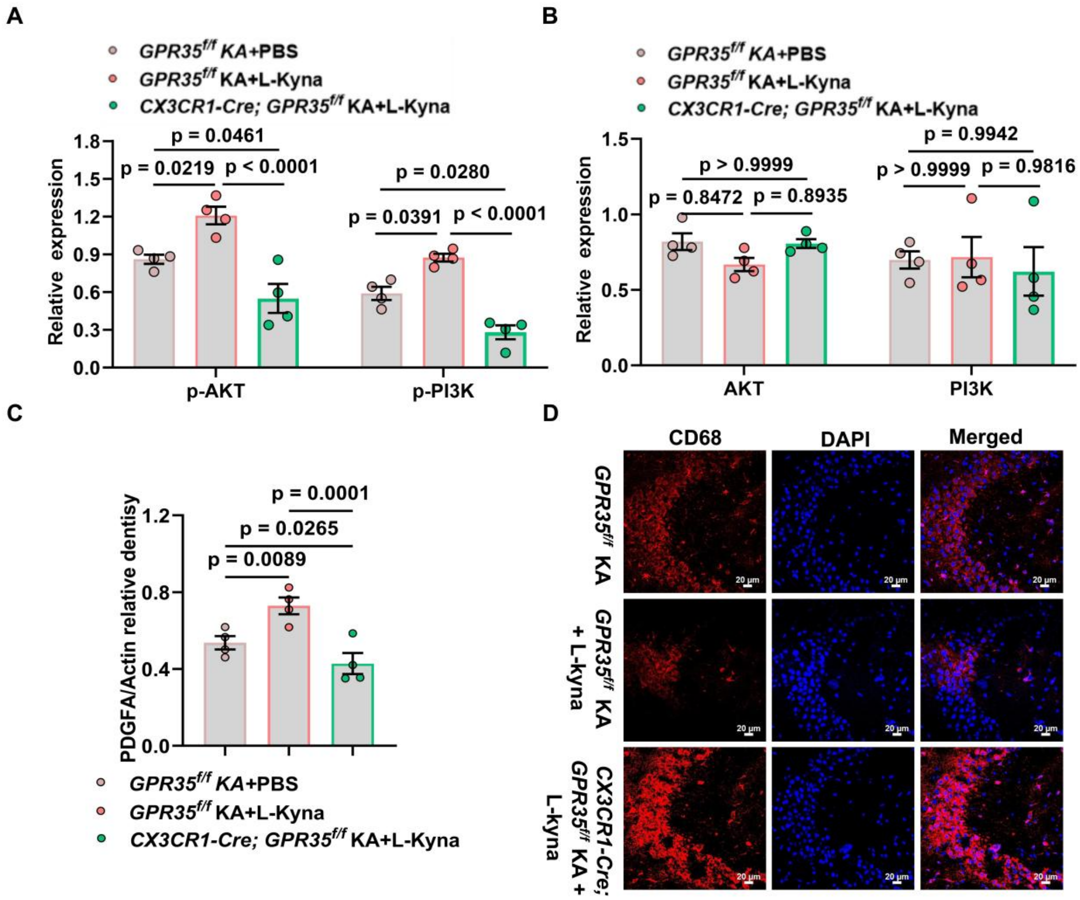

Supplementary Figure S15. ***In vivo*** **model treated with L-Kyna. Related to Figure 8.** A-C) Quantification of hippocampal p-AKT and p-PI3K (A), AKT and PI3K (B) and PDGFA (C) protein levels in *GPR35*$^{f/f}$ or *CX3CR1-Cre;GPR35*$^{f/f}$ mice treated with PBS or L-Kyna (100 mg kg^{-1} i.p qd) for 7 days at 4 weeks time points following KA-induced seizure or sham procedure in mice (n = 4). Two-way ANCOVA was used for analysis. D) Representative images of immunofluorescence staining showing the CD68 expression in hippocampal slices after *GPR35*$^{f/f}$ KA + PBS, *GPR35*$^{f/f}$ KA + L-kyna and *CX3CR1-Cre; GPR35*$^{f/f}$ KA + L-kyna. Scale bar: 20 μm. Brain tissue samples were obtained from the hippocampus (n = 3).

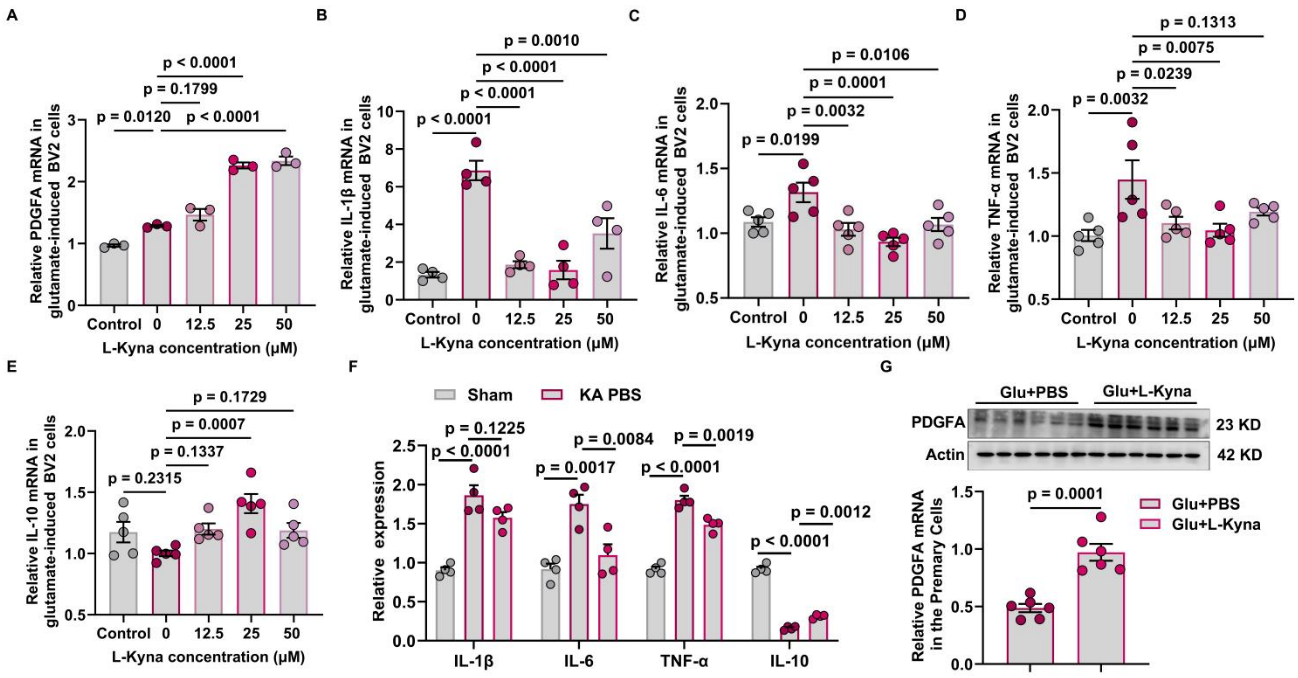

Supplementary Figure S16. *In vivo* model treated with L-Kyna. A-E) qRT-PCR analysis of PDGFA (A) (n = 5) and inflammatory cytokines expression of IL-1β (B), IL-6 (C), TNF-α (D), and IL-10 (E)) in Glu-induced BV2 cells treated with L-Kyna (12.5 μM, 25 μM, 50 μM) or vehicle control (n = 5). One-way analysis of covariance (ANCOVA) was used for analysis. F) qRT-PCR analysis of IL-1β, IL-6, TNF-α and IL-10 expression in Glu-induced BV2 cells treated with L-Kyna (25 μM) or vehicle control (n = 4). Two-way ANCOVA was used for analysis. G) Representative Western blot and quantification of hippocampal PDGFA protein levels in Glu-induced BV2 cells treated with L-Kyna (25 μM) or vehicle control (n = 4). Two-tailed unpaired *t* test was used for analysis. Data are presented as mean ± standard error of the mean (SEM).

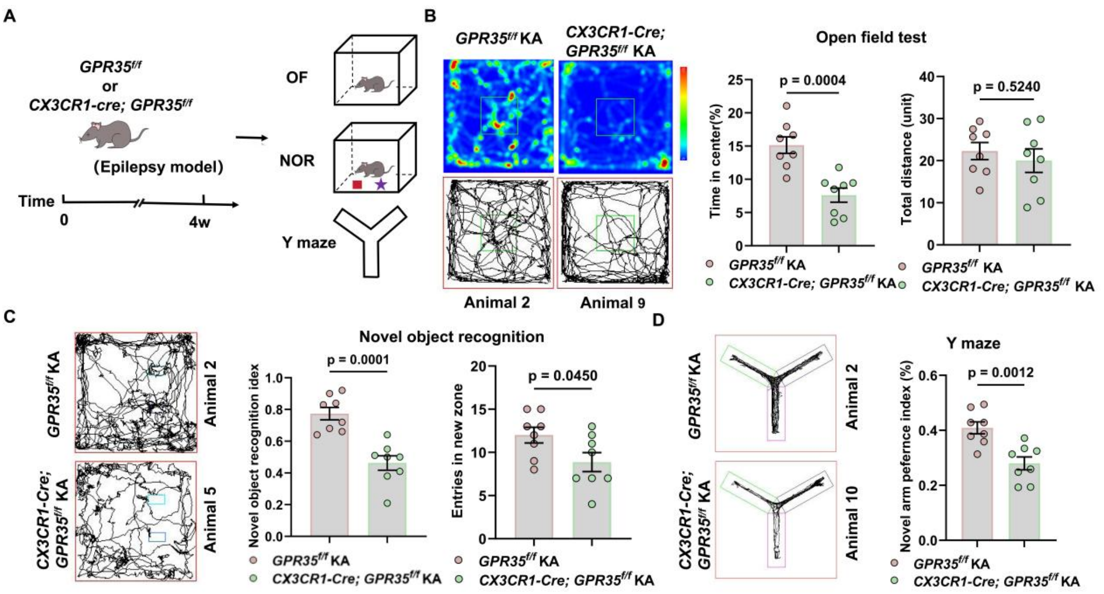

Supplementary Figure S17. Microglia-specific knockout of GPR35 on cognition following kainic acid-induced epilepsy. A) Schematic of the administration of treatments to epileptic mice and the cognitive testing timeline. B) (Left) Representative traces of a KA-induced *GPR35$^{f/f}$* mouse (*GPR35$^{f/f}$* KA) and KA-induced a *CX3CR1-Cre;GPR35$^{f/f}$* mouse (*CX3CR1-Cre;GPR35$^{f/f}$* KA) for 4 weeks in the open-field arena. The square area marked out the center zone for calculating center zone exploration in the open field. (Right) Quantification of the total distance traveled and time spent in the center zone in the open-field test (n = 8). One-way analysis of covariance (ANCOVA) was used for analysis. C) (Left) Representative traces of *GPR35$^{f/f}$* KA and *CX3CR1-Cre;GPR35$^{f/f}$* KA for 4 weeks by NOR. The blue rectangle area marked out the new zone. (Right) Quantification of the percentage of time exploring the novel object in NOR (n = 8). One-way ANCOVA was used for analysis. D) (Left) Representative traces from *GPR35$^{f/f}$* KA and *CX3CR1-Cre;GPR35$^{f/f}$* KA for 4 weeks in littermates by Y maze test. (Right) Quantification of time spent in novel arm in Y maze test (n = 8). One-way ANCOVA was used for analysis. Data are presented as mean ± standard error of the mean (SEM).

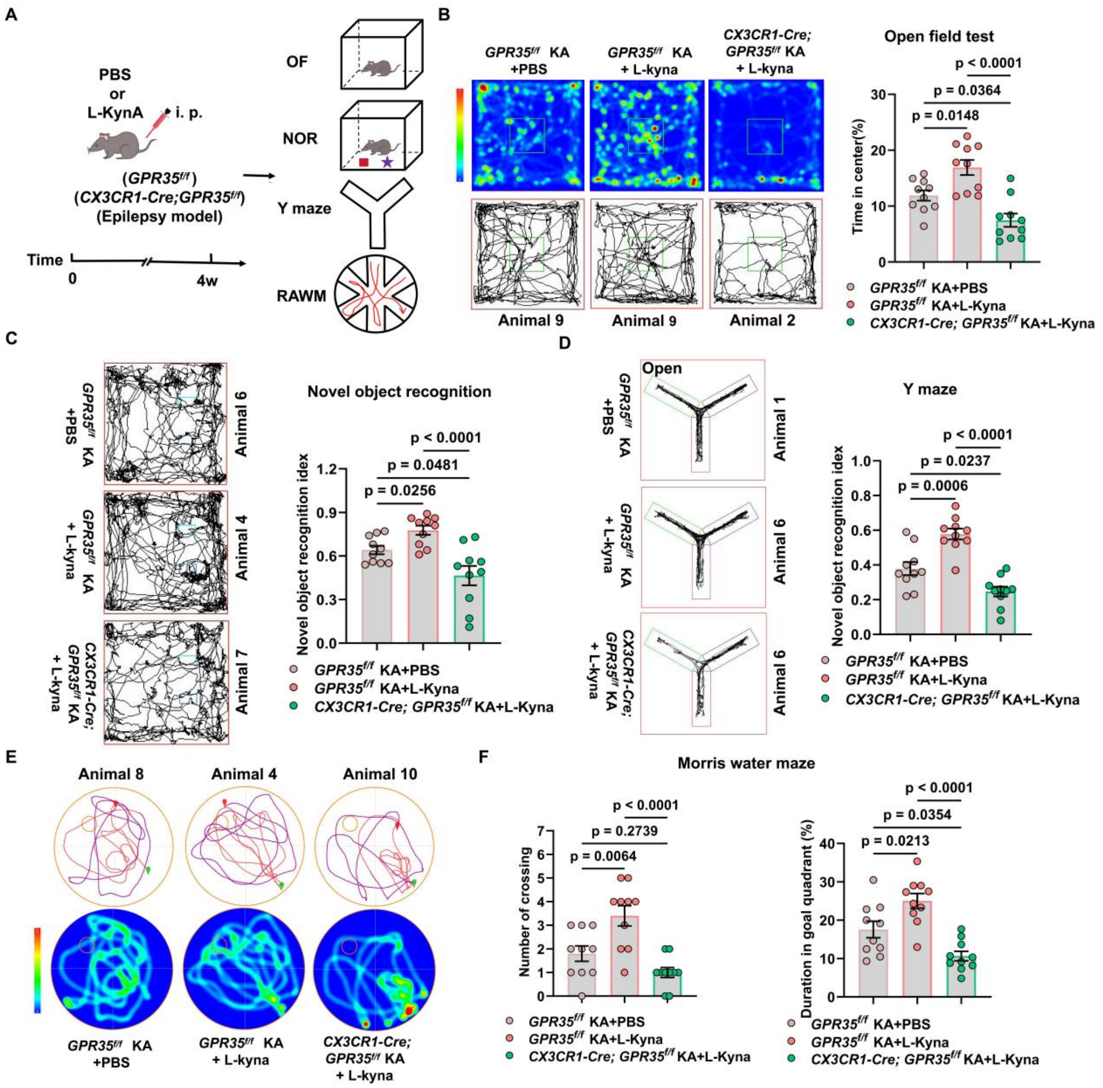

Supplementary Figure S18. The GPR35 agonist L-Kyna doses not improve cognitive impairment in KA-induced *CX3CR1-Cre;GPR35$^{f/f}$* mice. A) Schematic timeline illustrating drug administration and behavioral tests in epileptic mice. B) (Left) Representative movement traces of KA-induced *CX3CR1-Cre;GPR35$^{f/f}$* or *GPR35$^{f/f}$* mice treated with PBS or L-Kyna (100 mg kg^{-1}, i.p., once daily for 7 days during seizure latency) recorded 4 weeks post-treatment in the open field test. The square area marked out the center zone for calculating center zone exploration in the open field test. (Right) Quantification of the total distance traveled and exploring time into center in the open-field test (n = 10). One-way analysis of covariance (ANCOVA) was used for analysis. C) (Left) Representative traces of KA-induced *CX3CR1-Cre;GPR35$^{f/f}$* or *GPR35$^{f/f}$* mice treated with PBS or L-Kyna (100 mg kg^{-1}, i.p., once daily for 7 days during seizure latency) for 4 weeks by NOR. The blue rectangle area marked out the new zone. (Right) Quantification of the percentage of time

exploring the novel object in NOR (n = 10). One-way ANCOVA was used for analysis. E-F) Representative track chart of the probe test was displayed (E), quantification of the numbers of platform crossings and time spent in the goal quadrant (F) (n = 10). One-way ANCOVA was used for analysis. Data are presented as mean ± standard error of the mean (SEM).

Notes

- **Left-side images:** the normally exposed cropped raw bands, used for quantitative analysis.
- **Right-side images:** the bright-field exposed reconstructed membranes, assembled from cropped sections to verify sample loading consistency,

Figure 1E.

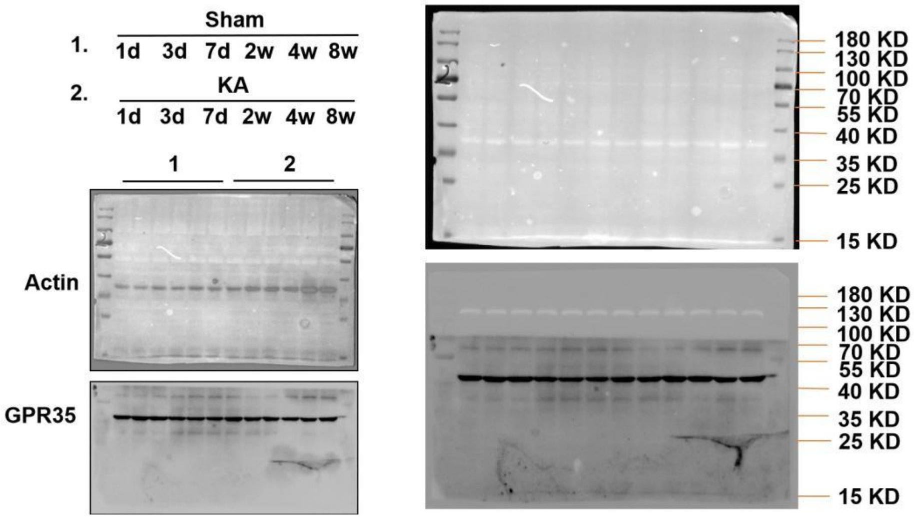

Figure 1E.

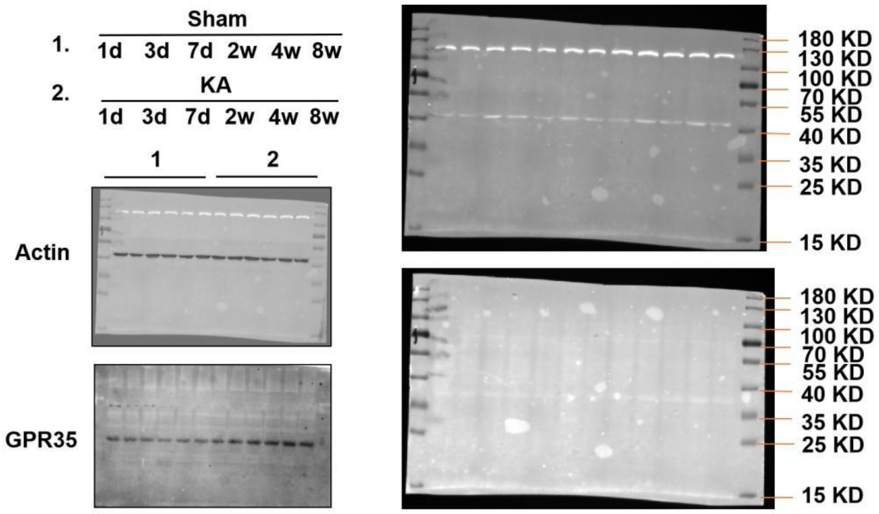

Figure 1E.

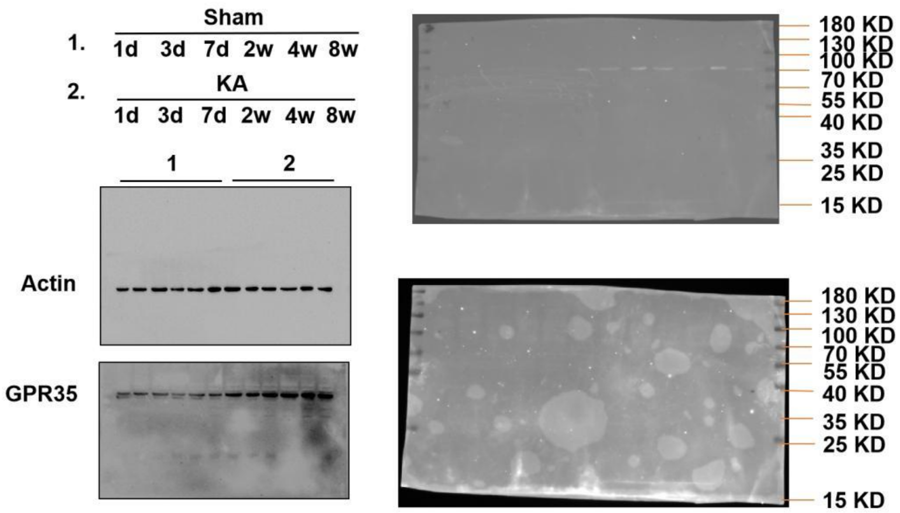

Figure 1E.

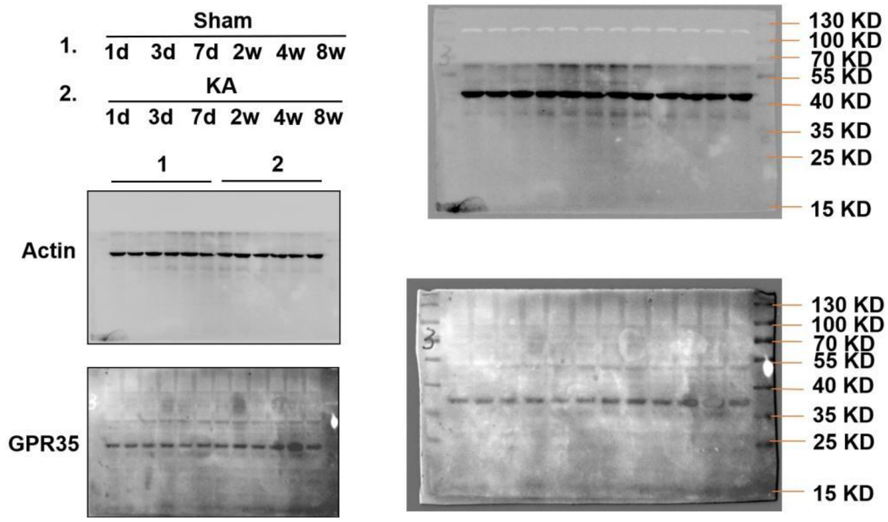

Figure 1L.

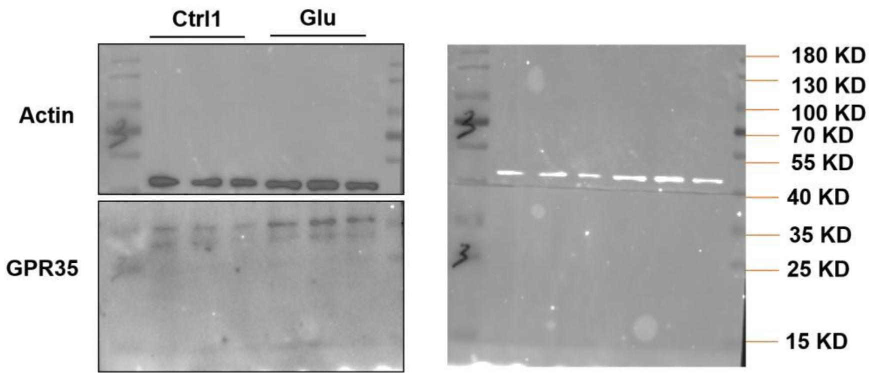

Figure 1M.

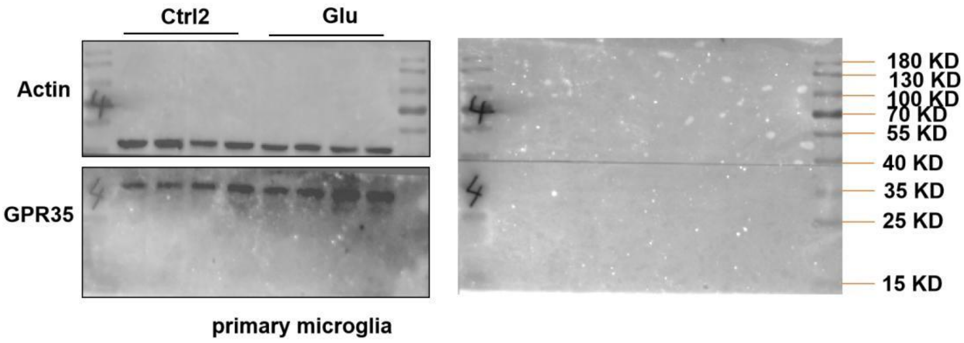

Figure 2C.

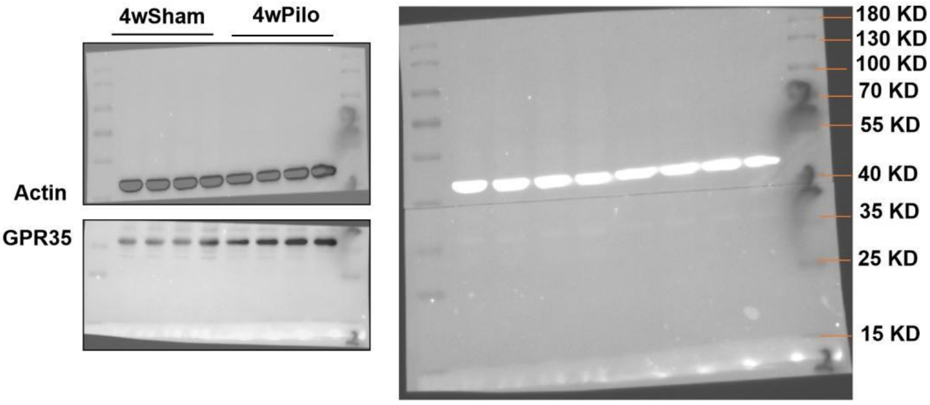

Figure 2J.

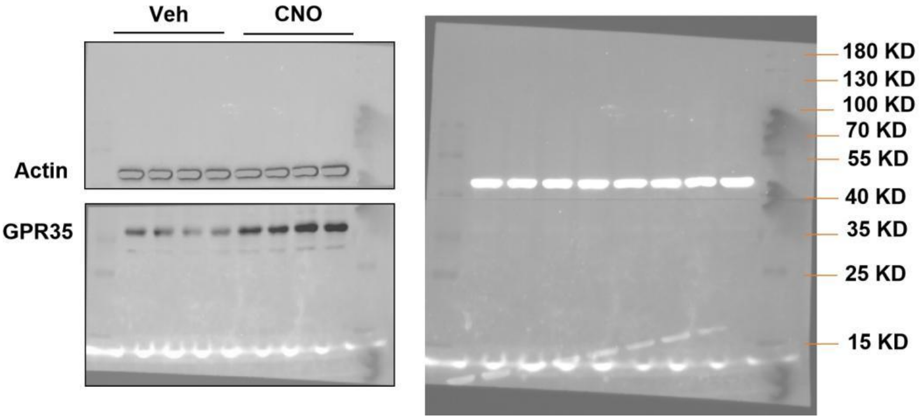

Figure 4C.

1.*GPR35*$^{f/f}$
2.*CaMKIIα-Cre; GPR35*$^{f/f}$
3.*CX3CR1-Cre; GPR35*$^{f/f}$

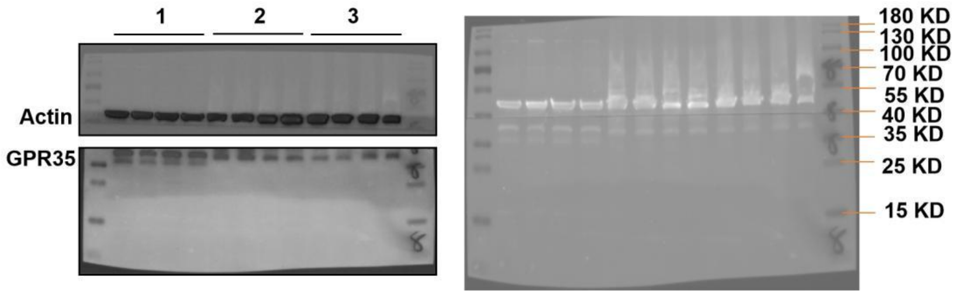

Figure 4D.

1.*GPR35*$^{f/f}$ KA
2.*CaMKIIα-Cre; GPR35*$^{f/f}$ KA
3.*CX3CR1-Cre; GPR35*$^{f/f}$ KA

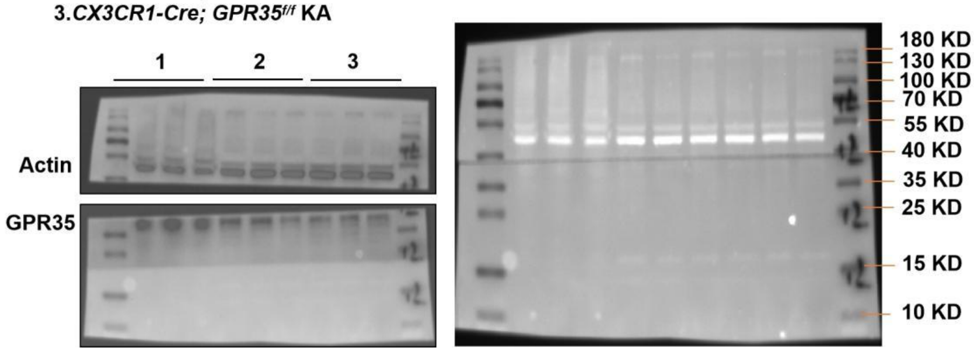

Figure 5G.

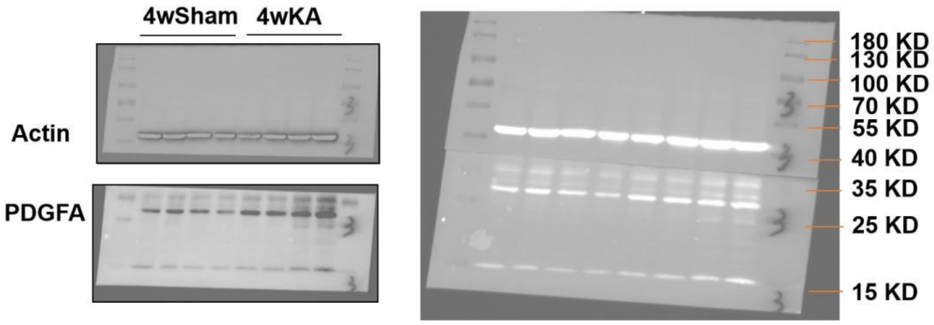

Figure 5H.

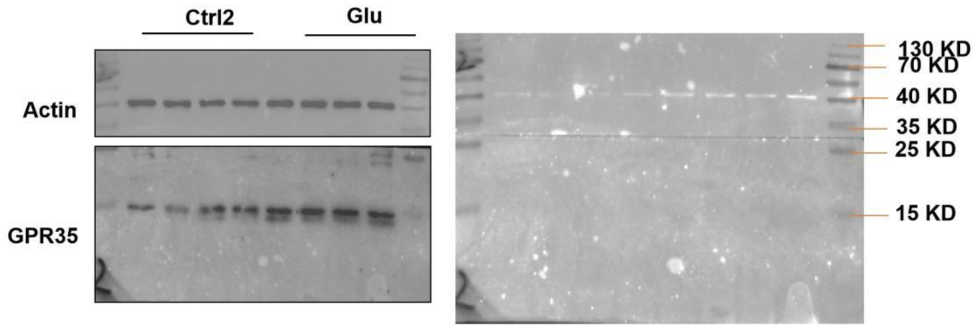

Figure 5I.

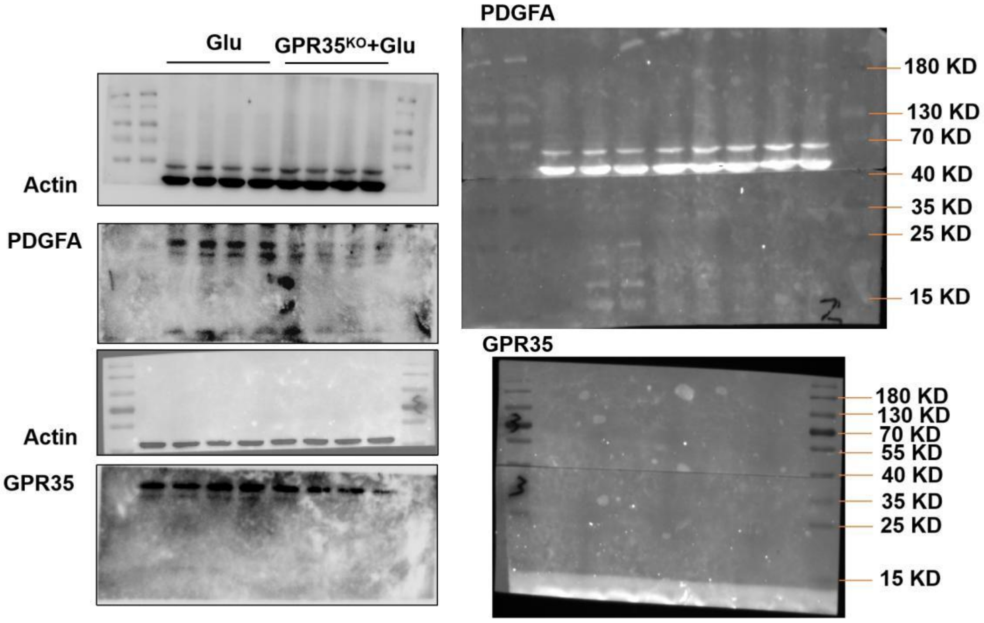

Figure 6C.

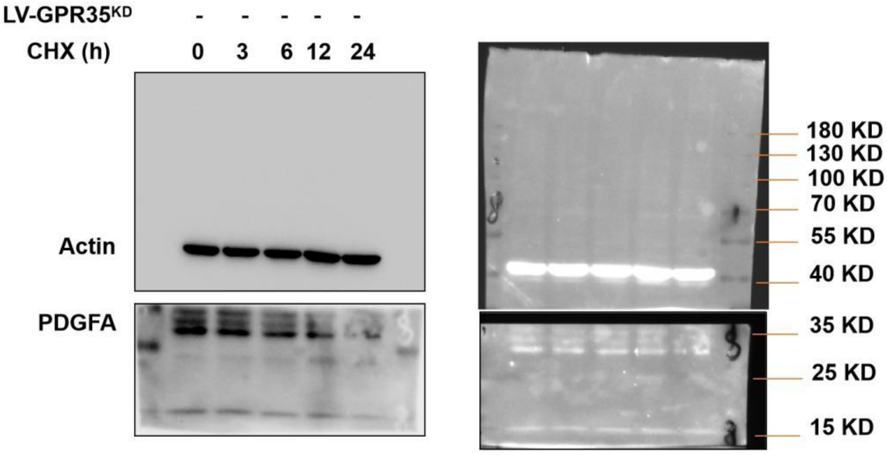

Figure 6C.

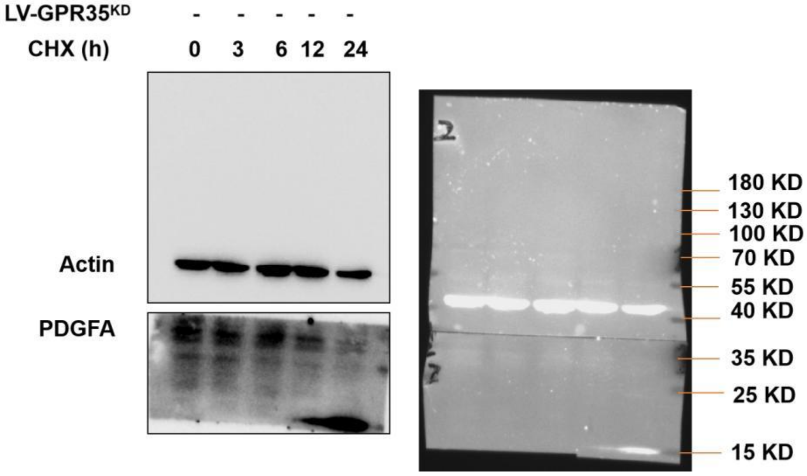

Figure 6C.

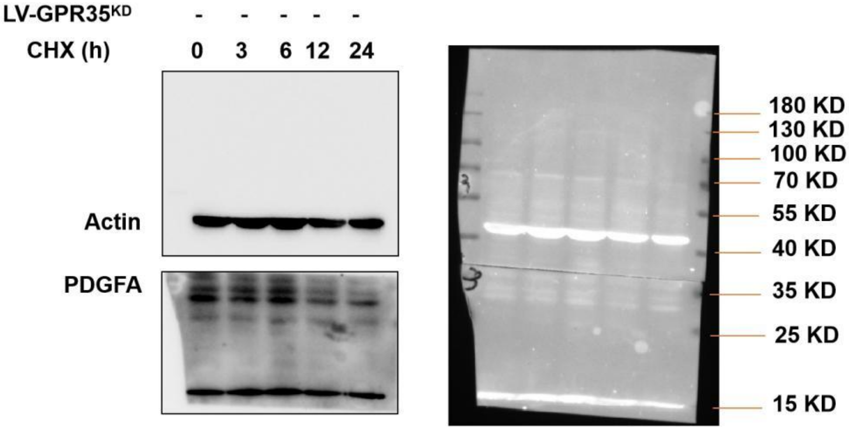

Figure 6C.

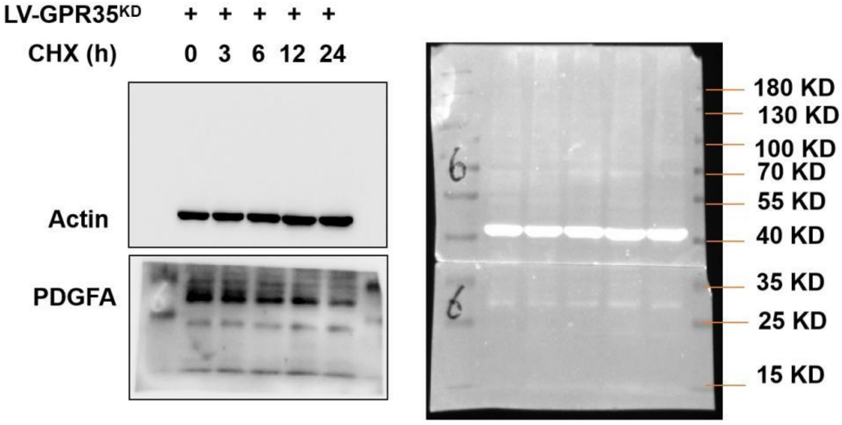

Figure 6C.

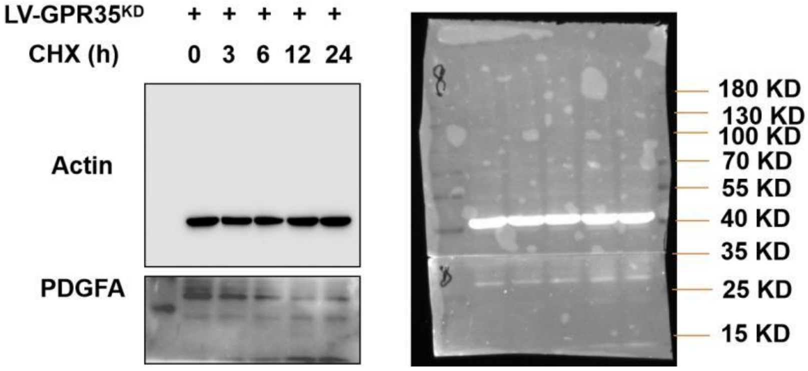

Figure 6C.

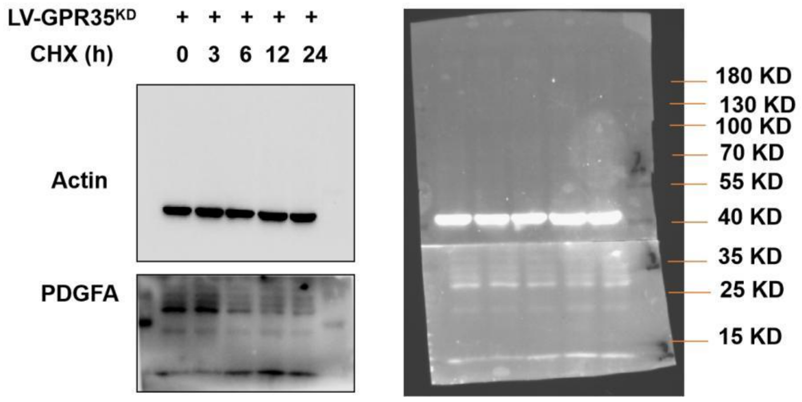

Figure 6D.

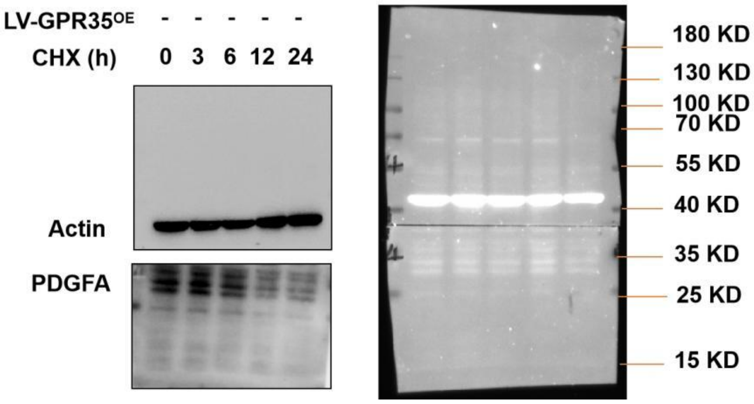

Figure 6D.

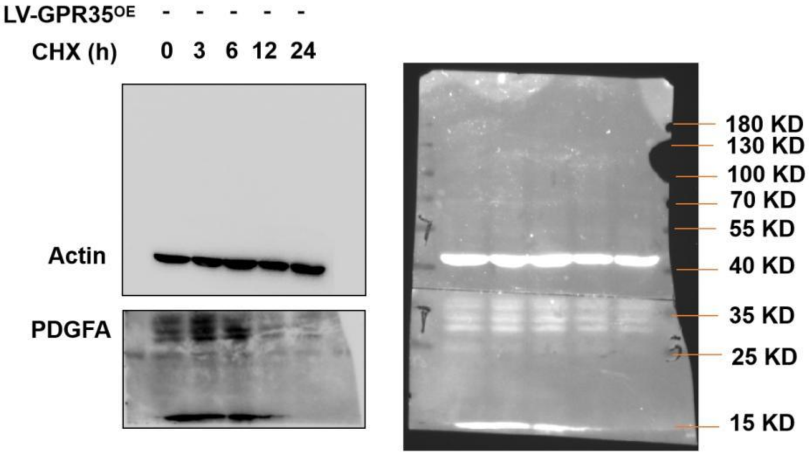

Figure 6D.

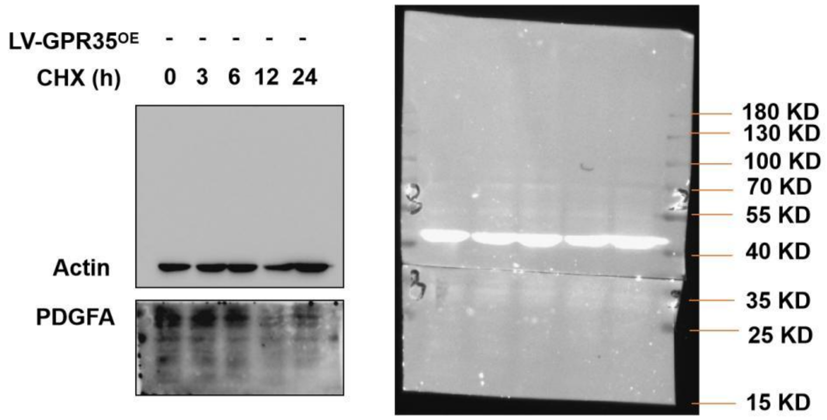

Figure 6D.

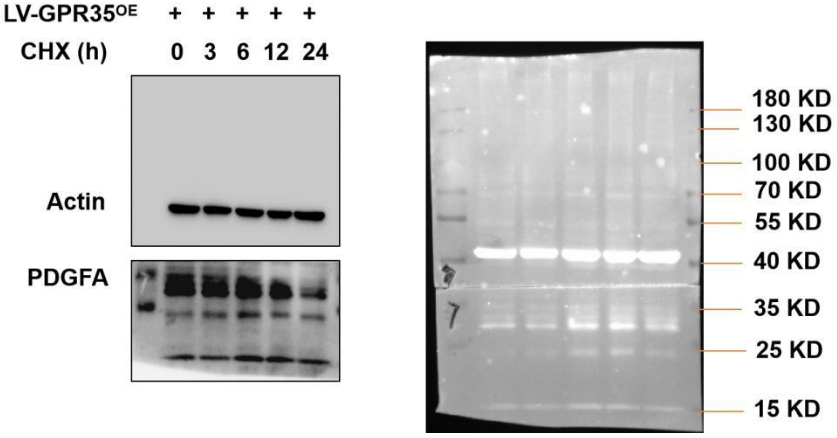

Figure 6D.

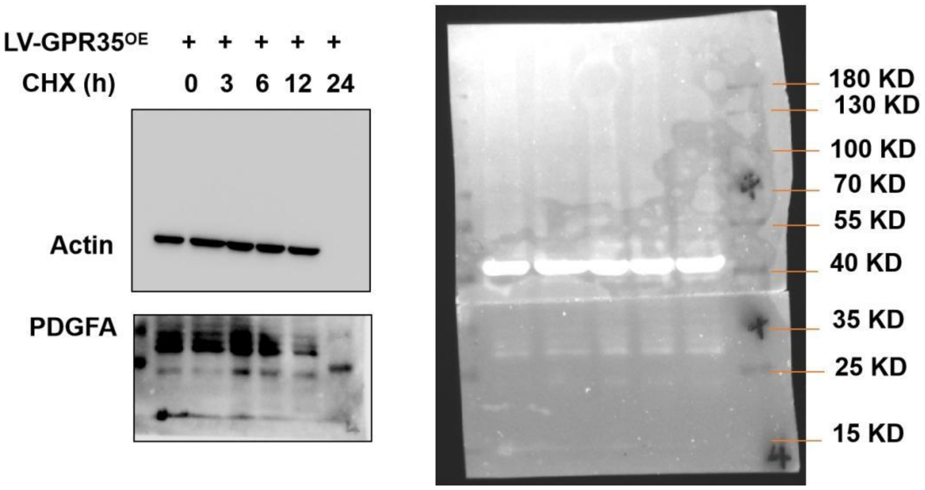

Figure 6D.

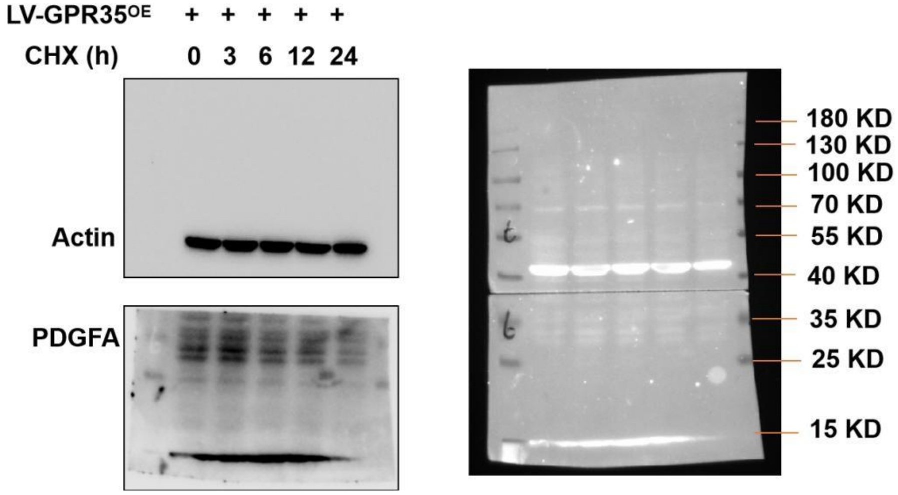

Figure 6F.

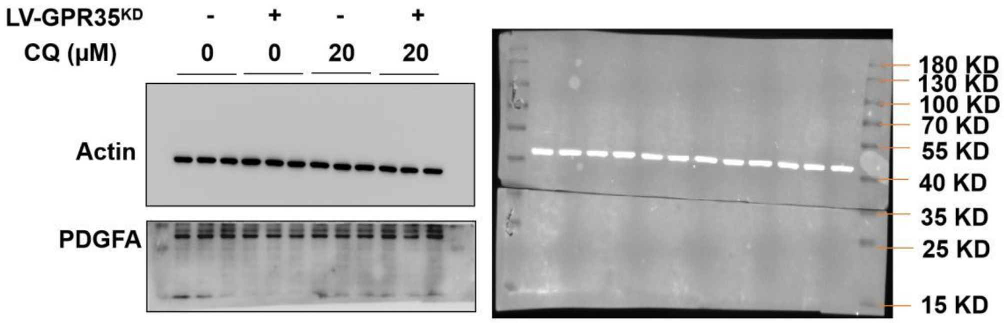

Figure 6F.

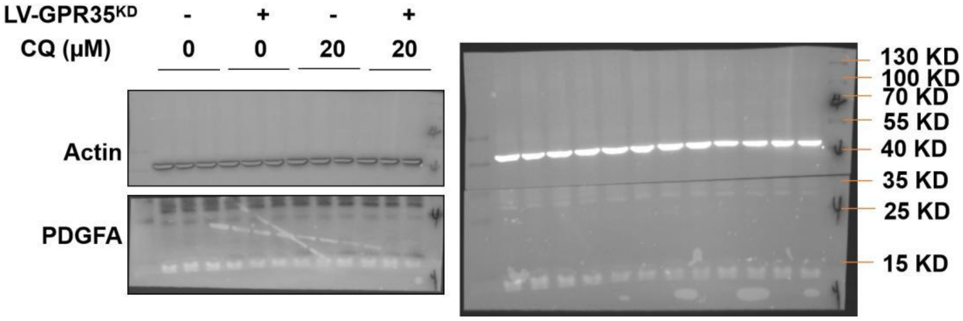

Figure 6G.

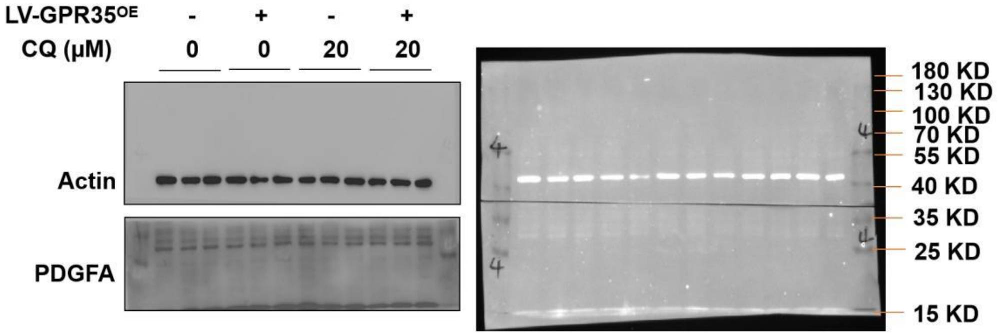

Figure 6G.

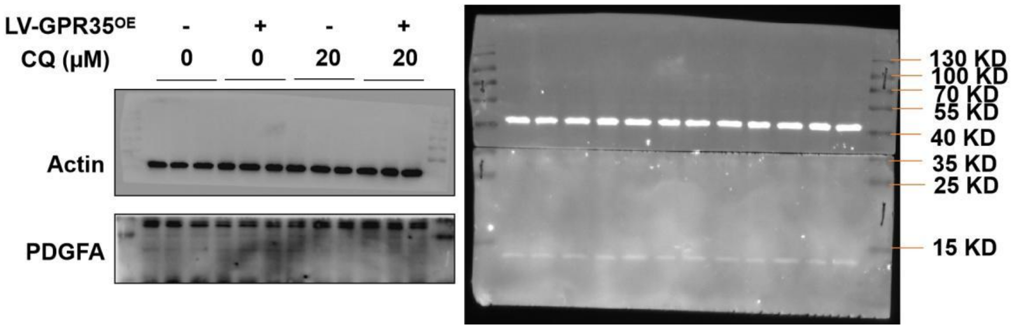

Figure 6H.

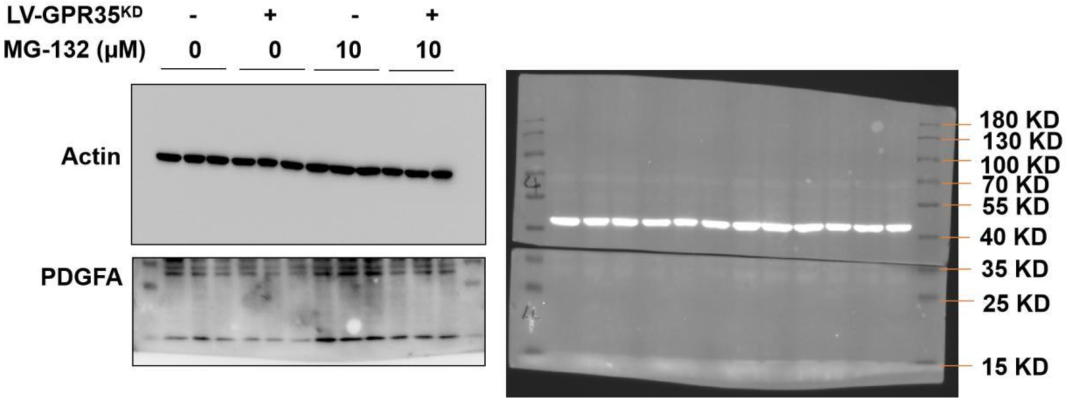

Figure 6H.

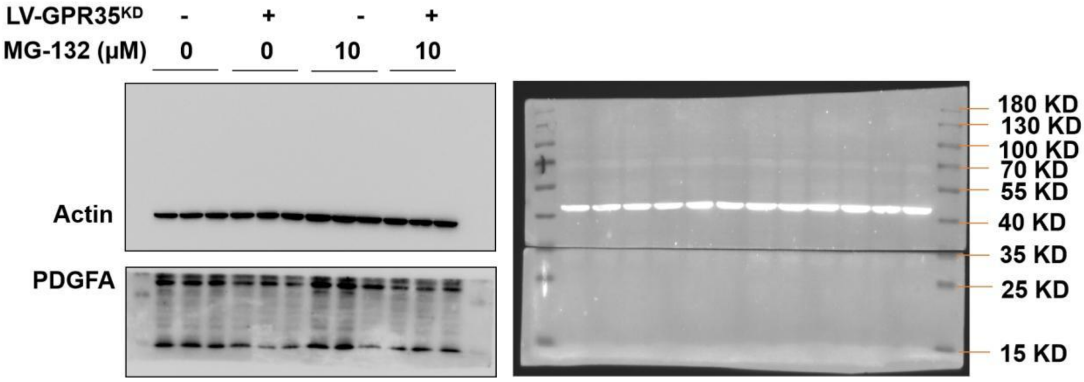

Figure 6I.

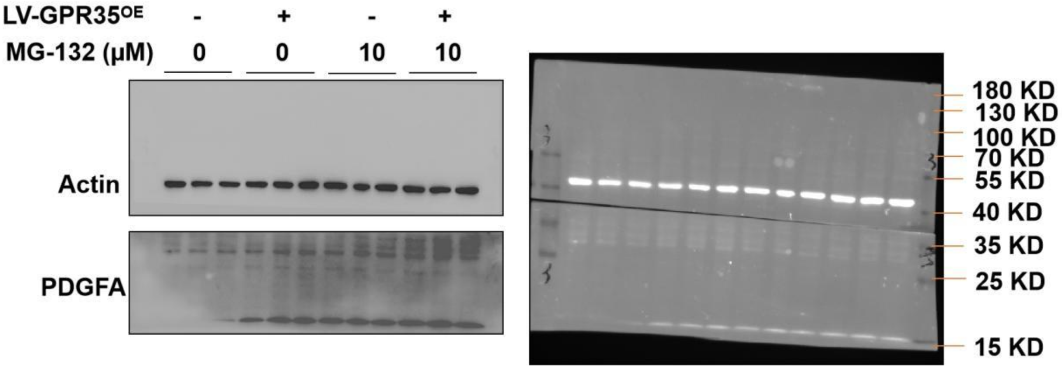

Figure 6I.

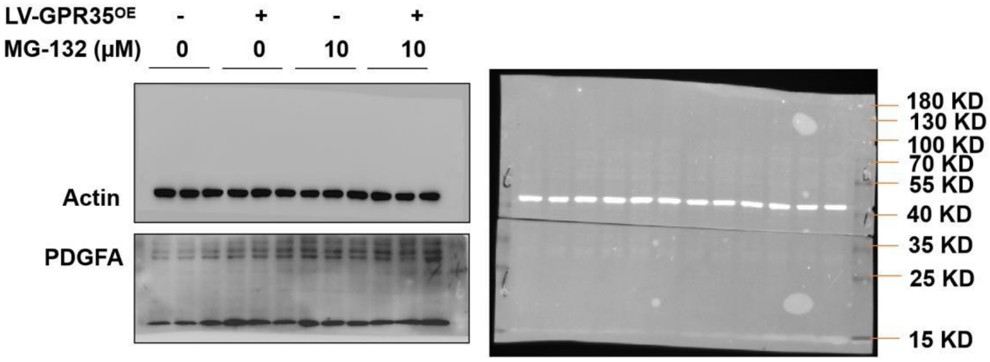

Figure 6J.

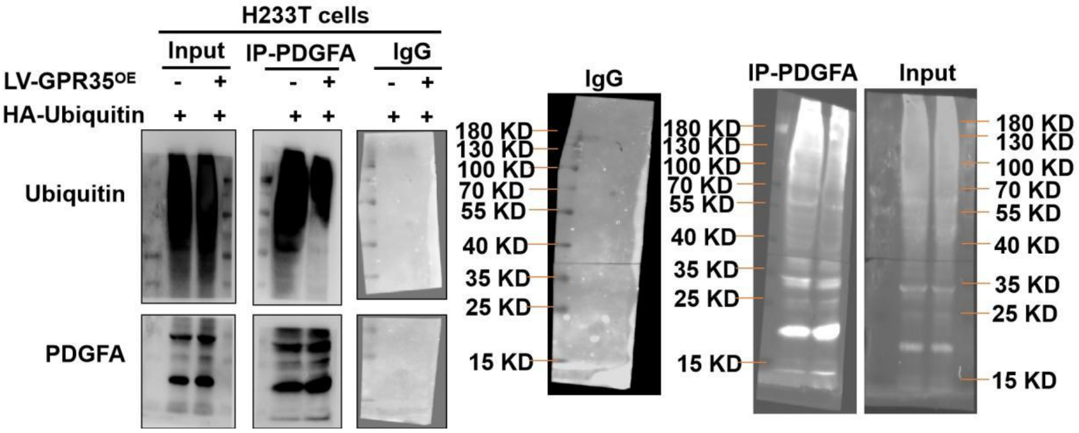

Figure 6K.

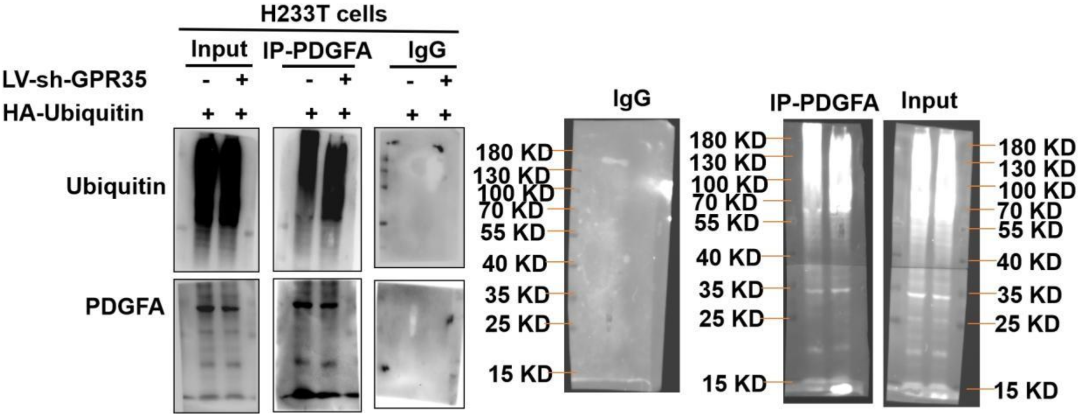

Figure 7B.

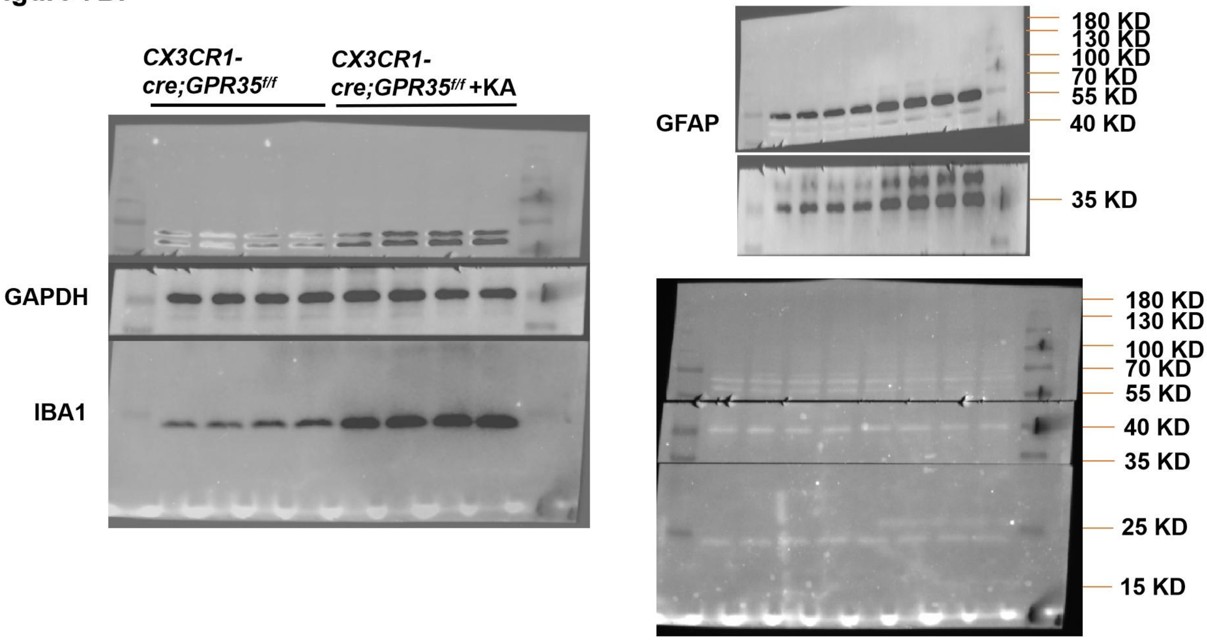

Figure 7C.

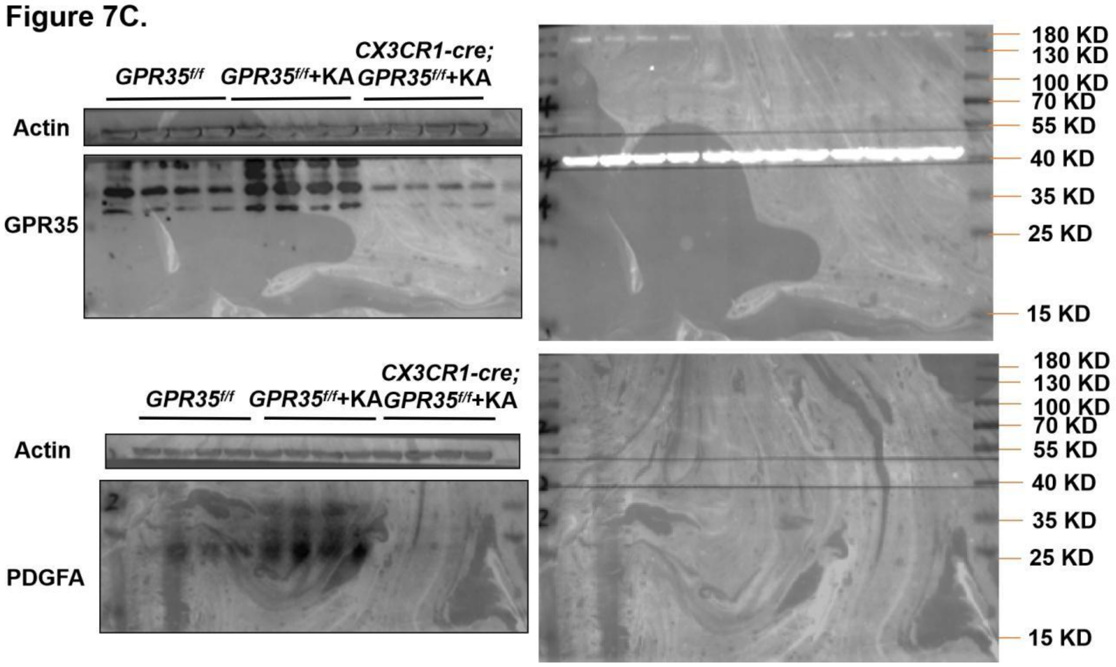

Figure 7C.

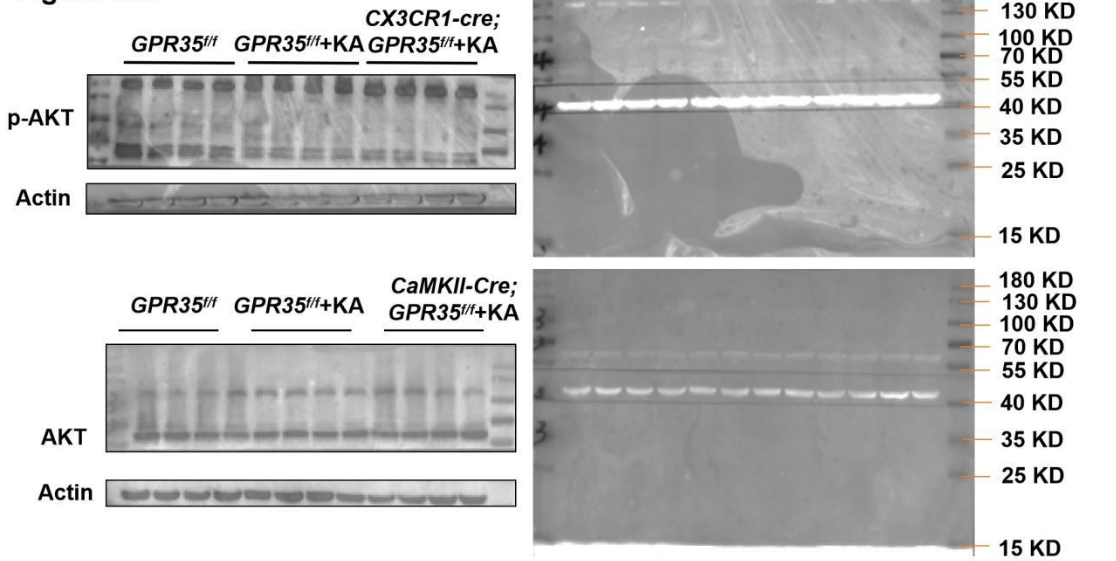

Figure 7C.

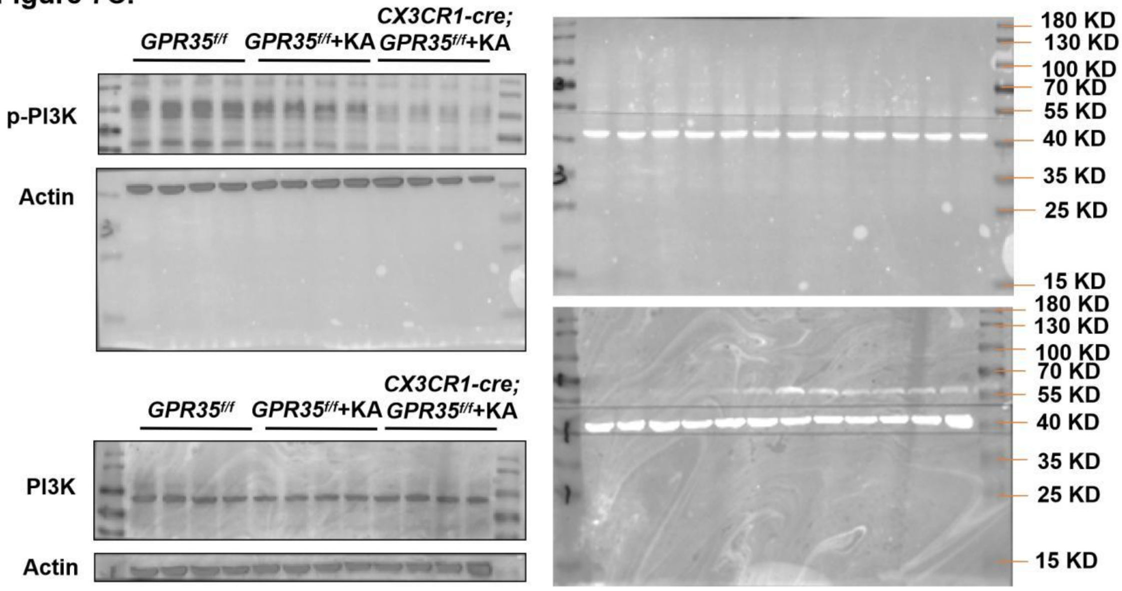

Figure 8D.

1. *CX3CR1-cre* KA
2. *CX3CR1-cre* + AAV-Con2 KA
3. *CX3CR1-cre* + PDGFA^{KD} KA

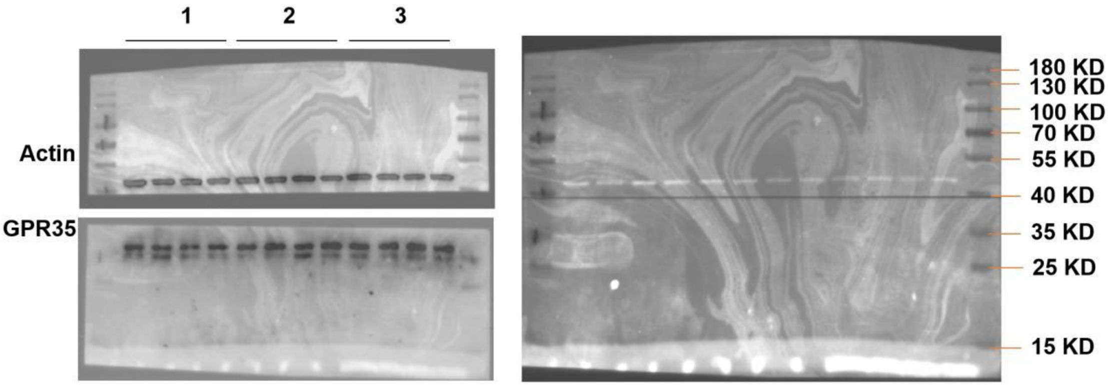

Figure 8D.

1. *CX3CR1-cre* KA
2. *CX3CR1-cre* + AAV-Con2 KA
3. *CX3CR1-cre* + PDGFA^{KD} KA

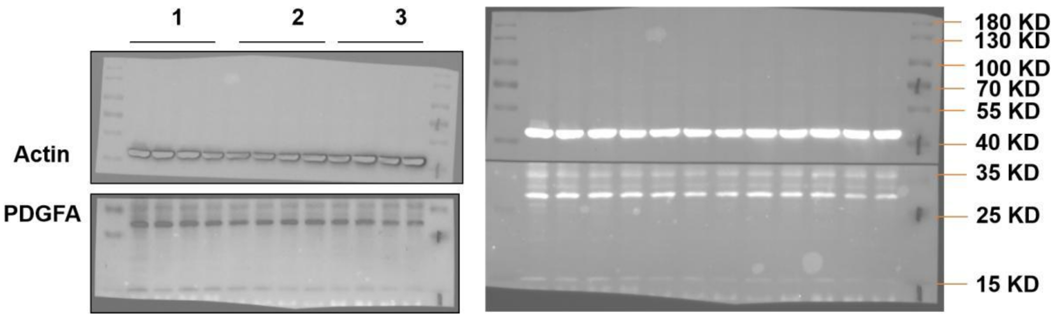

Figure 8J.

1. *GPR35*$^{f/f}$ + AAV-Con3 KA
2. *CX3CR1-cre; GPR35*$^{f/f}$ + AAV-Con3 KA
3. *GPR35*$^{f/f}$ + PDGFA^{OE} KA
4. *CX3CR1-cre; GPR35*$^{f/f}$ + PDGFA^{OE} KA

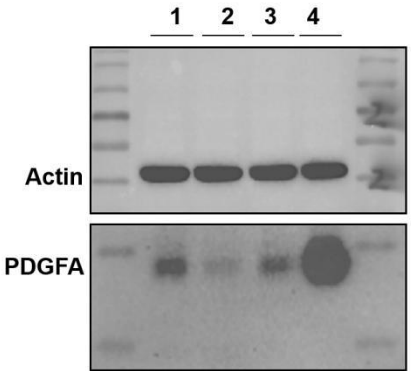

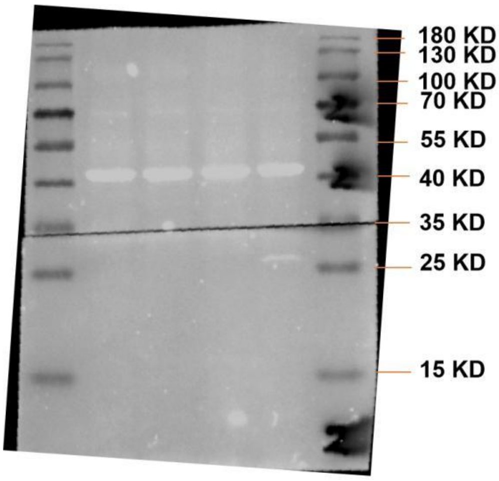

Figure 8J.

1. *GPR35*$^{f/f}$ + AAV-Con3 KA
2. *CX3CR1-cre; GPR35*$^{f/f}$ + AAV-Con3 KA
3. *GPR35*$^{f/f}$ + PDGFA^{OE} KA
4. *CX3CR1-cre; GPR35*$^{f/f}$ + PDGFA^{OE} KA

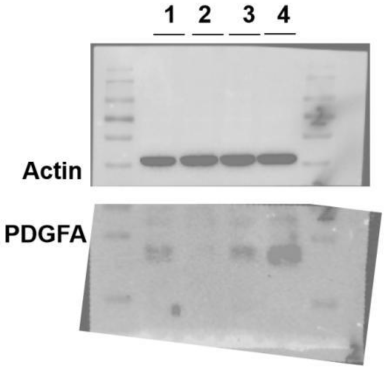

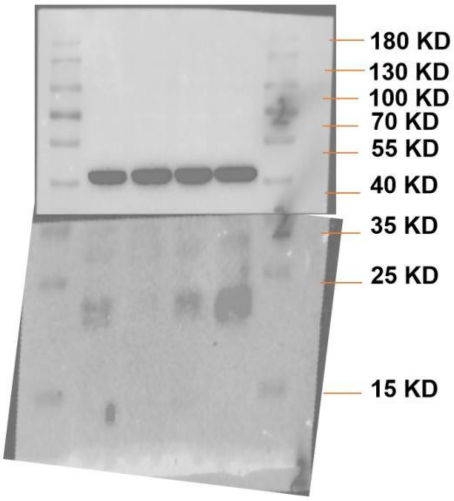

Figure 8J.

1. *GPR35*$^{f/f}$ + AAV-Con3 KA
2. *CX3CR1-cre; GPR35*$^{f/f}$ + AAV-Con3 KA
3. *GPR35*$^{f/f}$ + PDGFA^{OE} KA
4. *CX3CR1-cre; GPR35*$^{f/f}$ + PDGFA^{OE} KA

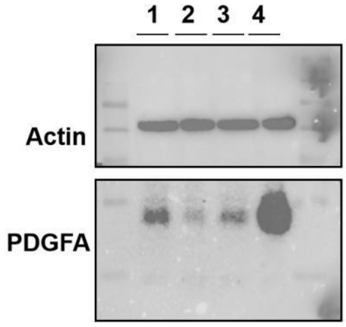

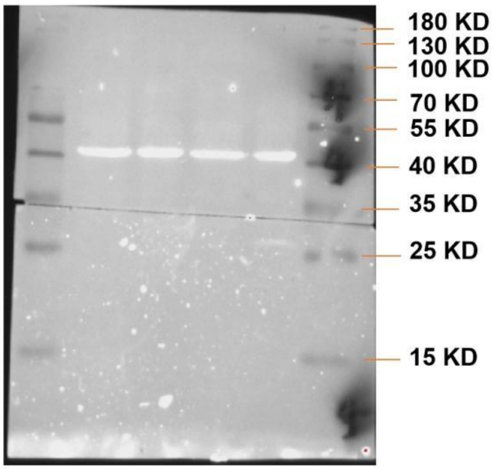

Figure 8J.

1. *GPR35^f/f^* + AAV-Con3 KA
2. *CX3CR1-cre; GPR35^f/f^* + AAV-Con3 KA
3. *GPR35^f/f^* + PDGFA^{OE} KA
4. *CX3CR1-cre; GPR35^f/f^* + PDGFA^{OE} KA

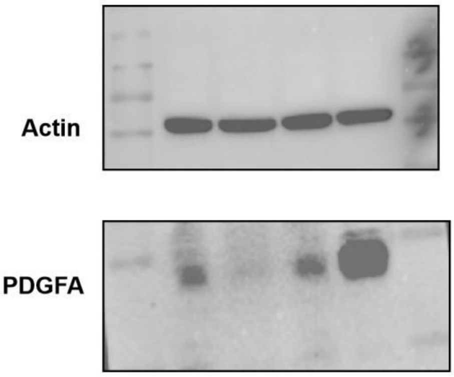

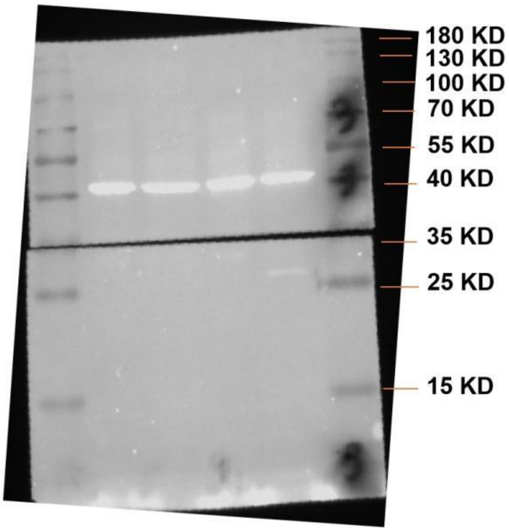

Figure 8L.

1. *GPR35^f/f^* + AAV-Con3 KA
2. *CX3CR1-cre; GPR35^f/f^* + AAV-Con3 KA
3. *GPR35^f/f^* + PDGFA^{OE} KA
4. *CX3CR1-cre; GPR35^f/f^* + PDGFA^{OE} KA

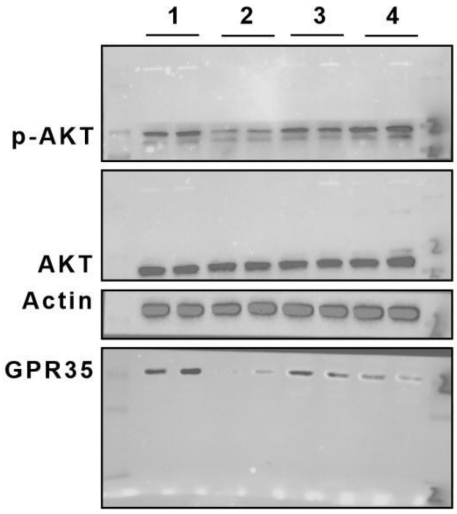

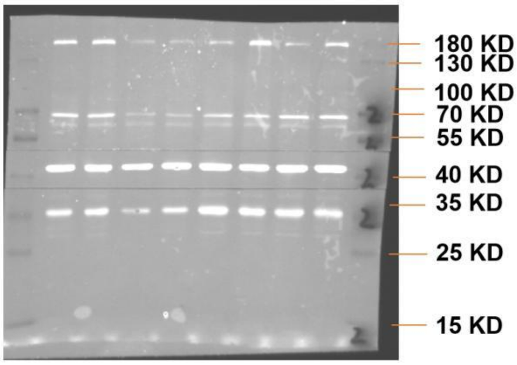

Figure 8L.

1. *GPR35^f/f^* + AAV-Con3 KA
2. *CX3CR1-cre; GPR35^f/f^* + AAV-Con3 KA
3. *GPR35^f/f^* + PDGFA^{OE} KA
4. *CX3CR1-cre; GPR35^f/f^* + PDGFA^{OE} KA

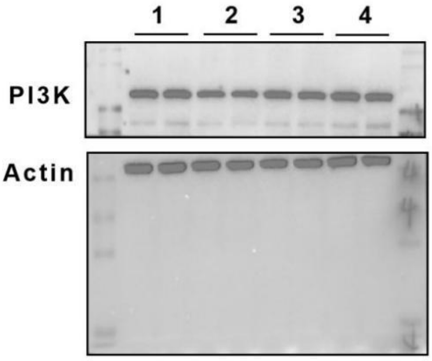

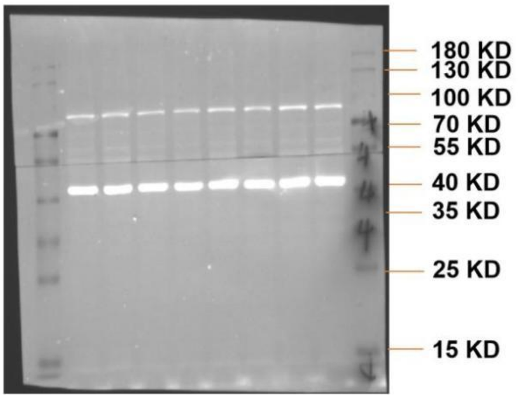

Figure 8L.

1. *GPR35^f/f^* + AAV-Con3 KA
2. *CX3CR1-cre; GPR35^f/f^* + AAV-Con3 KA
3. *GPR35^f/f^* + PDGFA^OE^ KA
4. *CX3CR1-cre; GPR35^f/f^* + PDGFA^OE^ KA

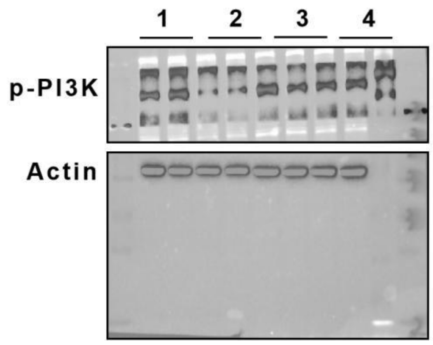

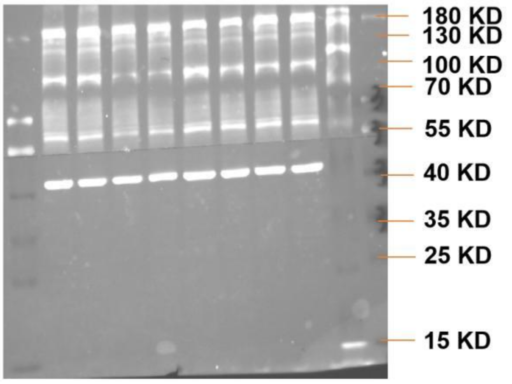

Figure 9D.

1. *GPR35^f/f^* KA
2. *GPR35^f/f^* KA + L-kyna
3. *CX3CR1-Cre; GPR35^f/f^* KA + L-kyna

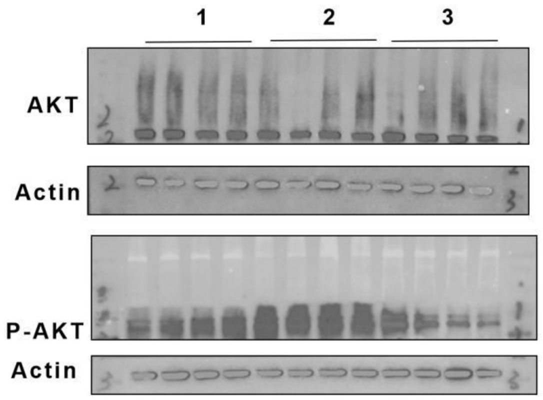

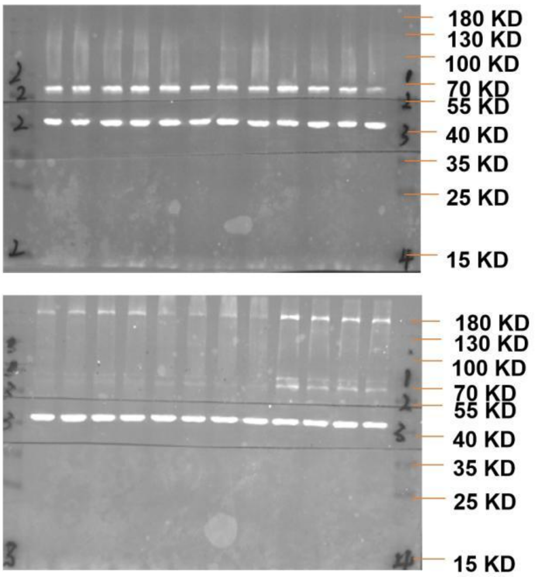

Figure 9D.

1. *GPR35^f/f^* KA
2. *GPR35^f/f^* KA + L-kyna
3. *CX3CR1-Cre; GPR35^f/f^* KA + L-kyna

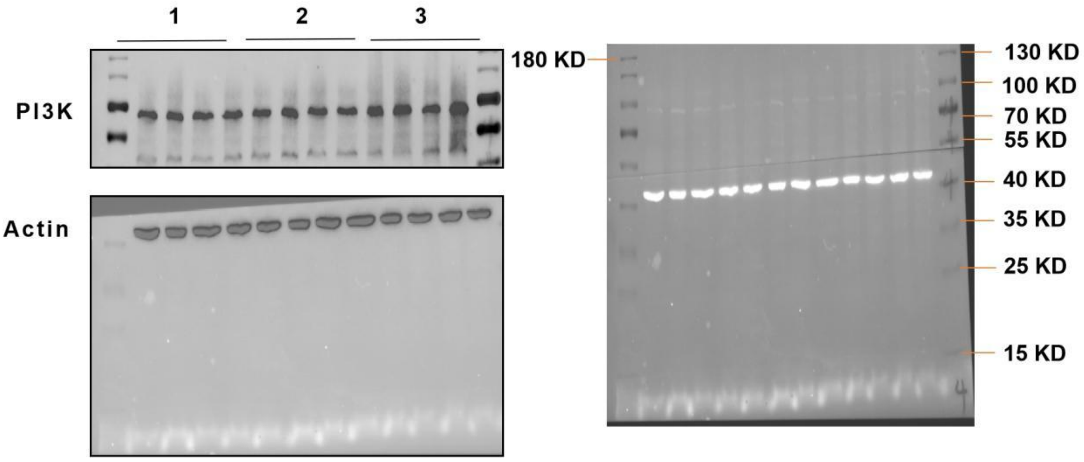

Figure 9D.

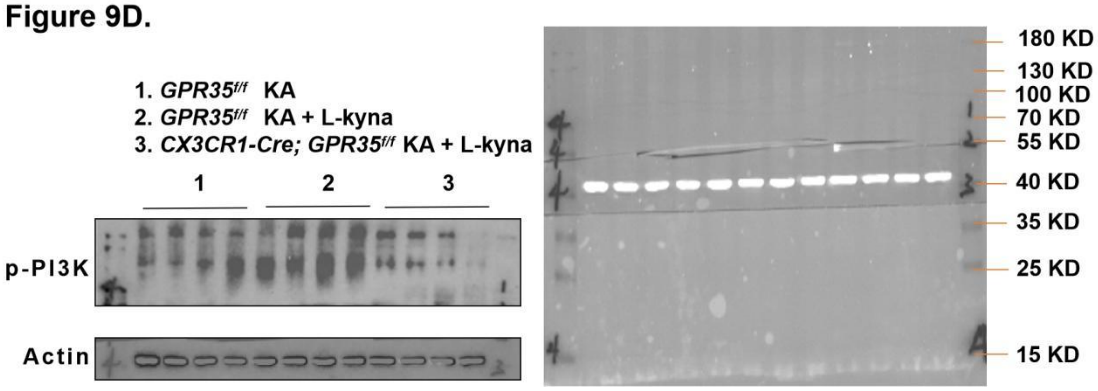

Figure 9D.

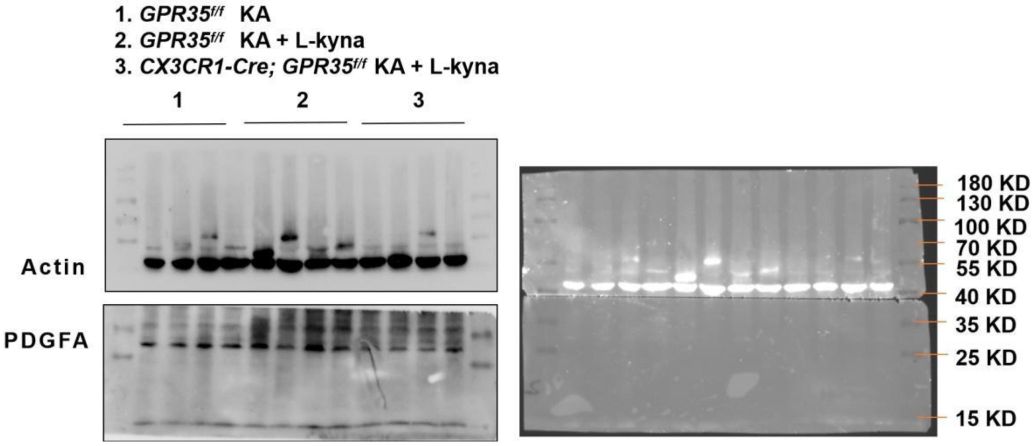

Supplementary Figure S8A.

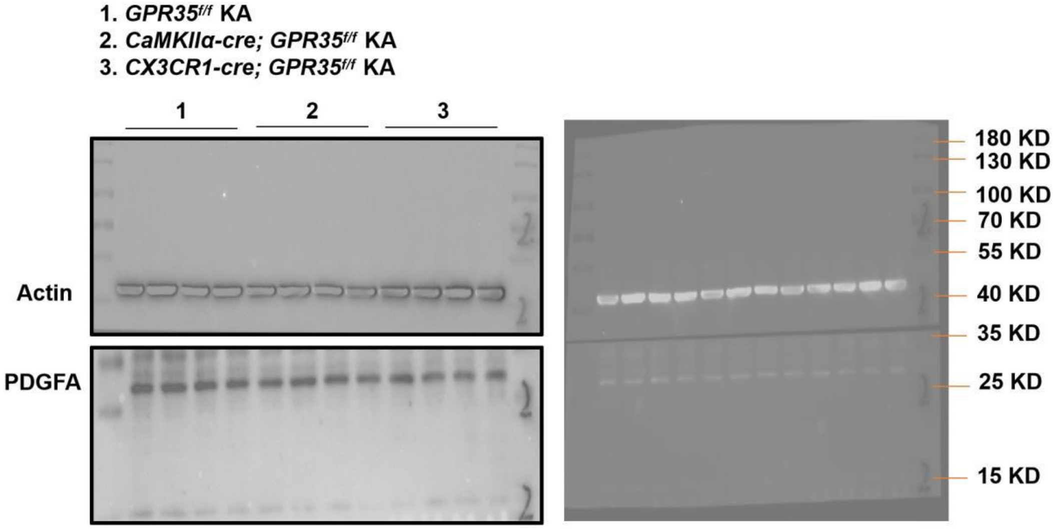

Supplementary Figure S8C.

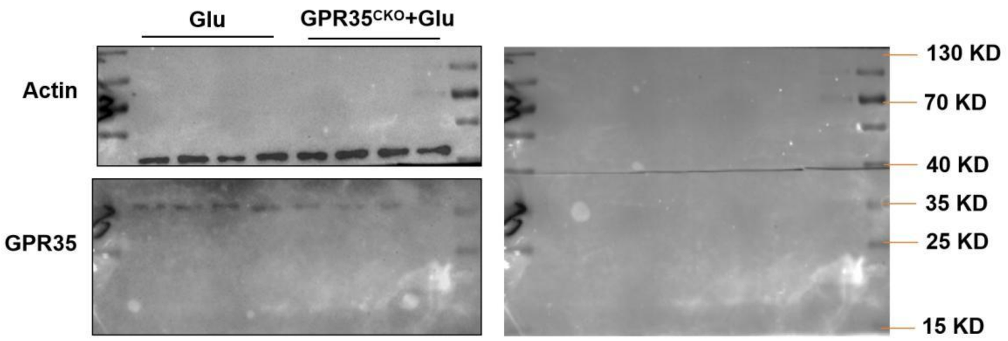

cKO: *CX3CR1-cre; GPR35$^{f/f}$*

Supplementary Figure S8C.

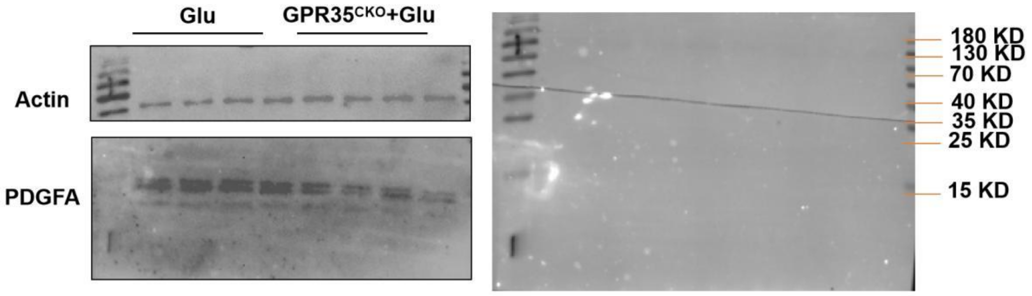

cKO: *CX3CR1-cre; GPR35$^{f/f}$*

Supplementary Figure S9.

Verification of GPR35^{OE}

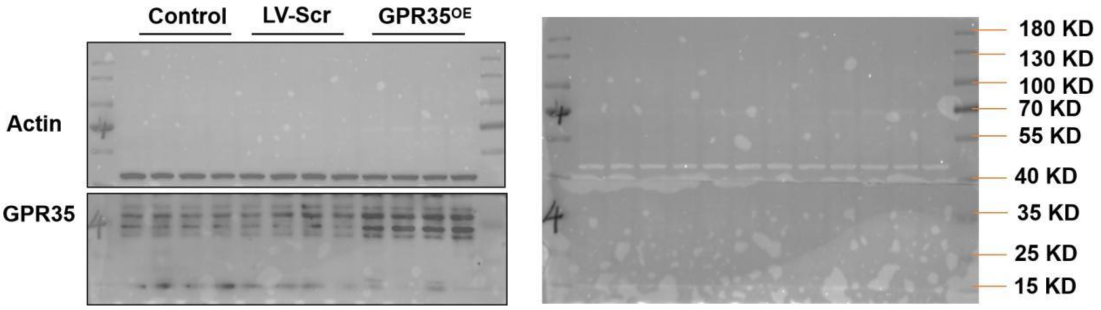

Supplementary Figure S16G.

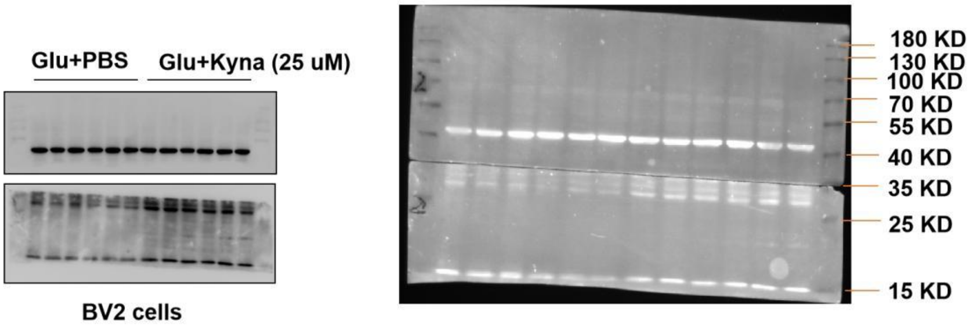

Supplementary Figure S13.

Heart

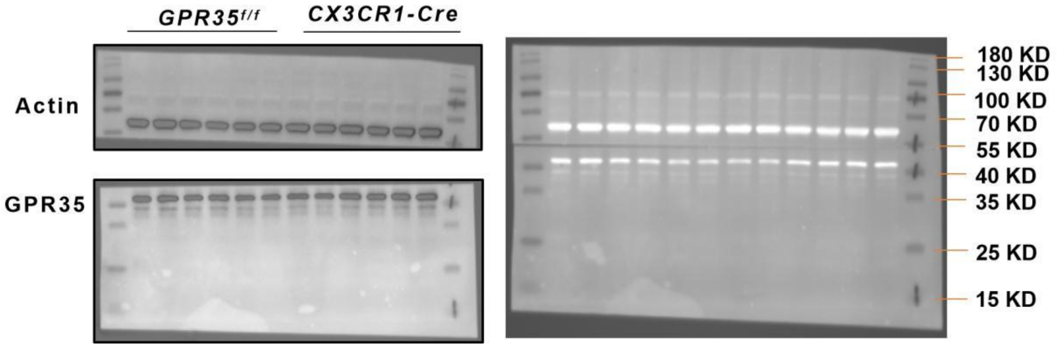

Supplementary Figure S13.

Heart

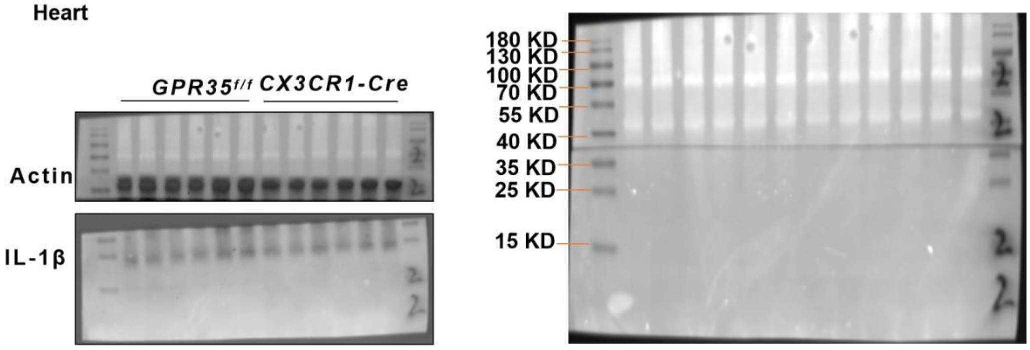

Supplementary Figure S13.

Heart

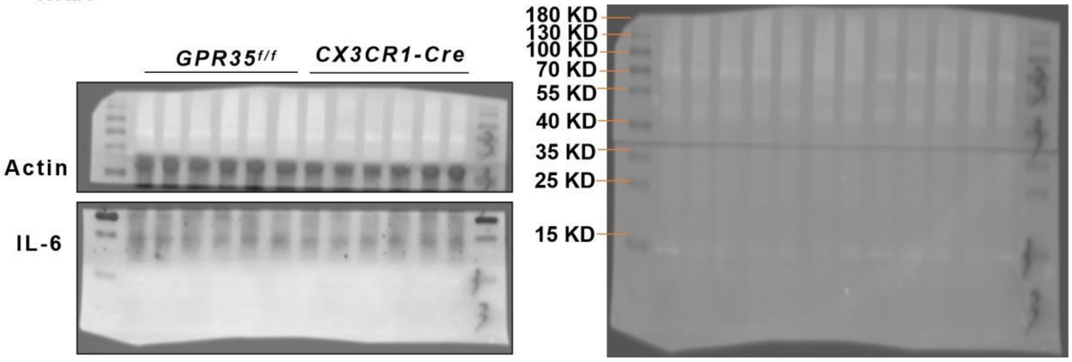

Supplementary Figure S13.

Heart

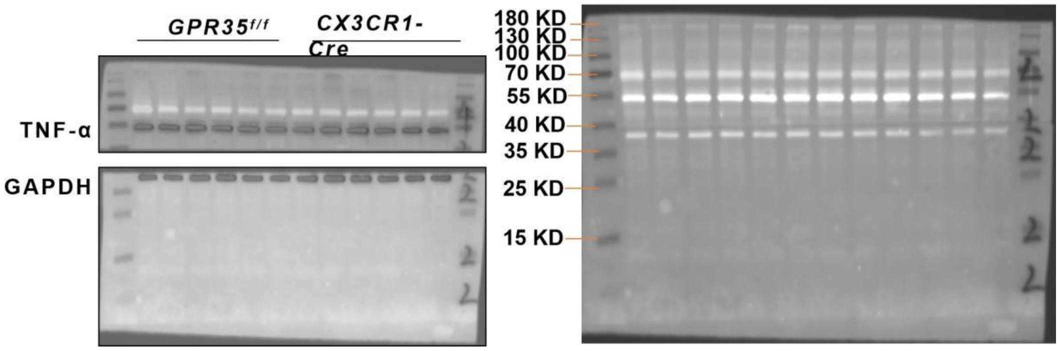

Supplementary Figure S13.

Liver

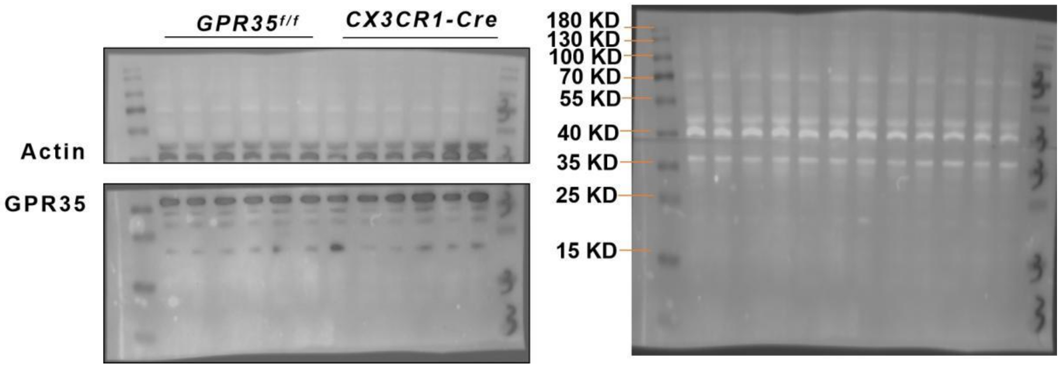

Supplementary Figure S13.

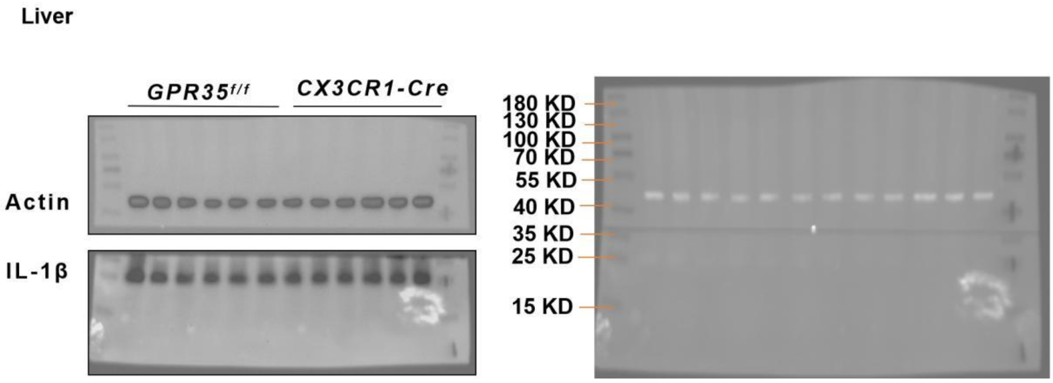

Supplementary Figure S13.

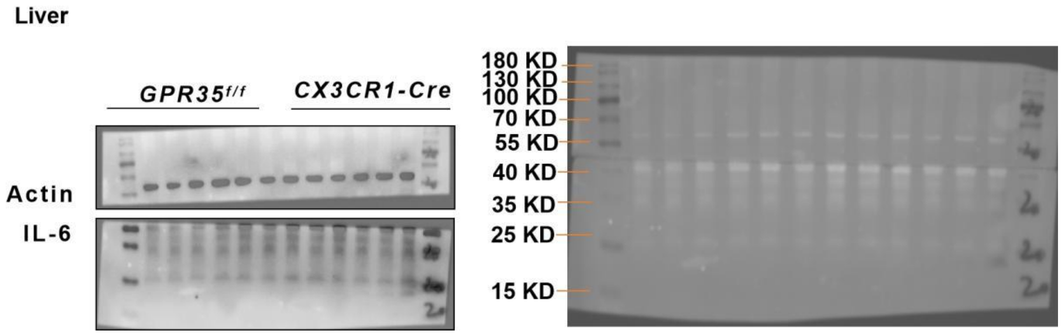

Supplementary Figure S13.

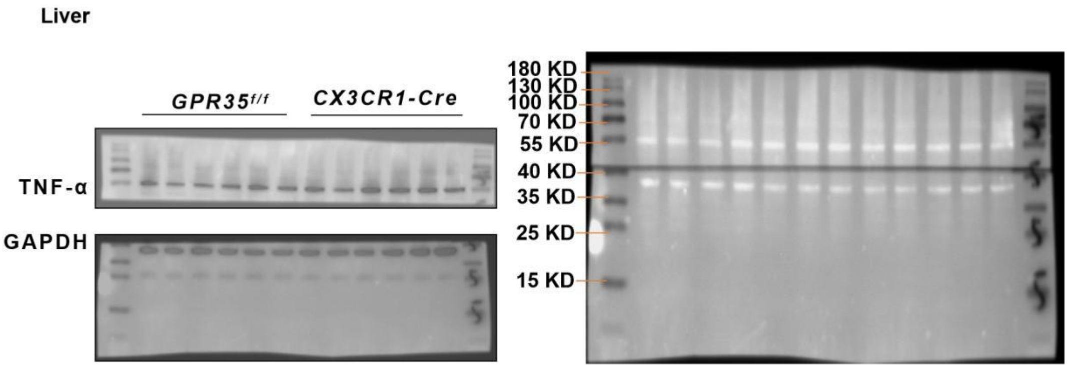

Supplementary Figure S13.

Spleen

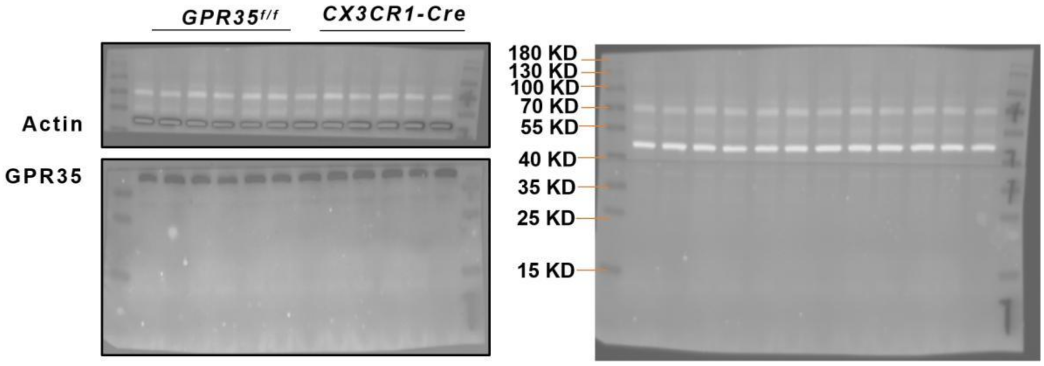

Supplementary Figure S13.

Spleen

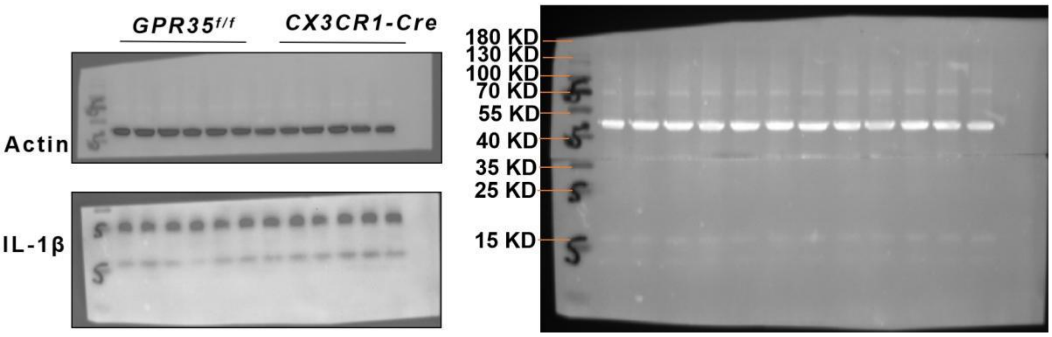

Supplementary Figure S13.

Spleen

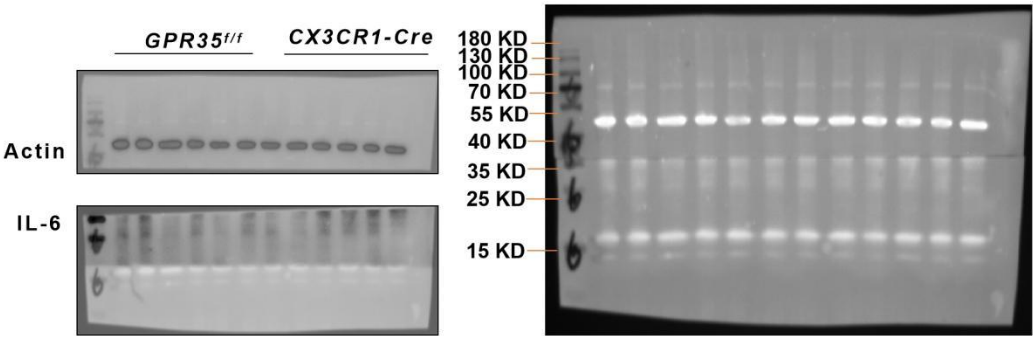

Supplementary Figure S13.

Spleen

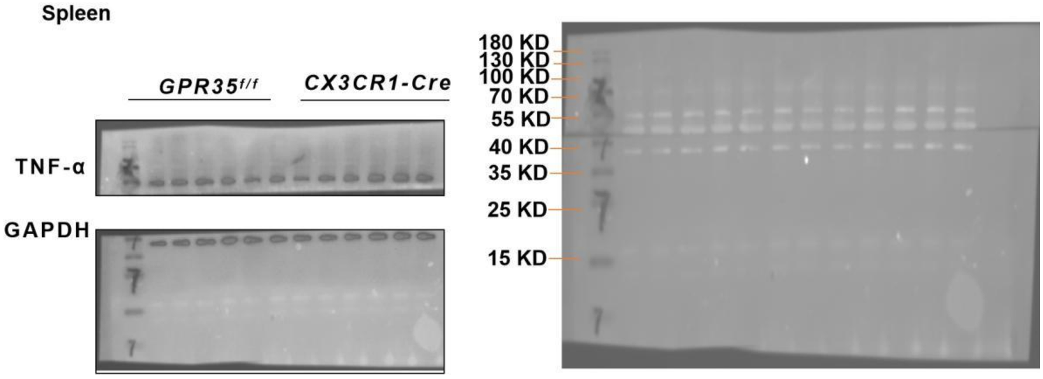

Supplementary Figure S13.

Lung

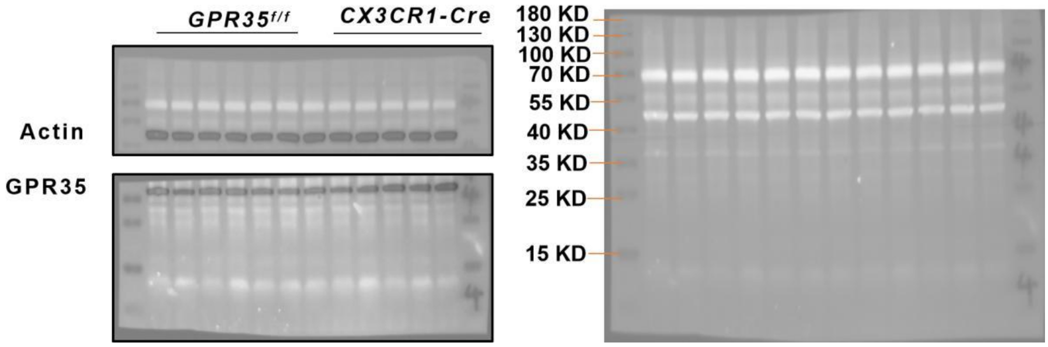

Supplementary Figure S13.

Lung

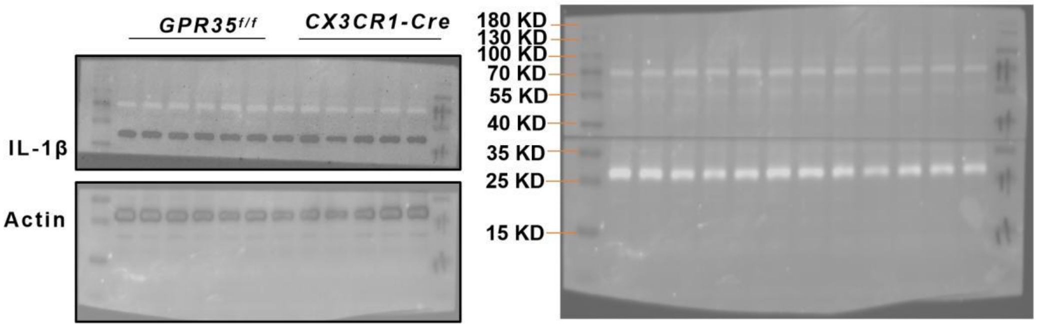

Supplementary Figure S13.

Lung

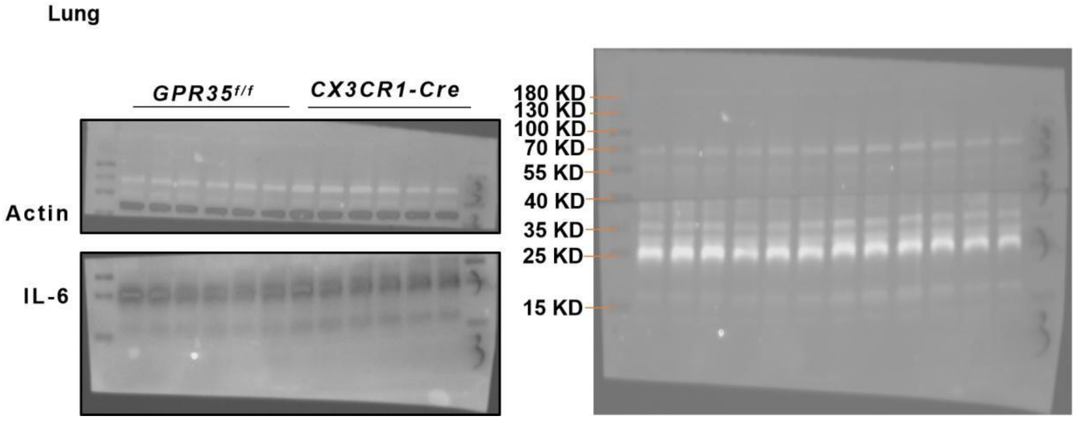

Supplementary Figure S13.

Lung

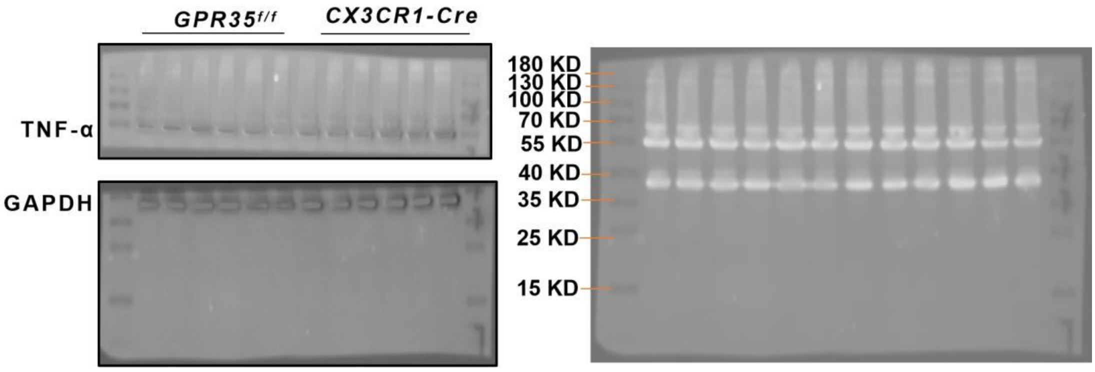

Supplementary Figure S13.

Kidney

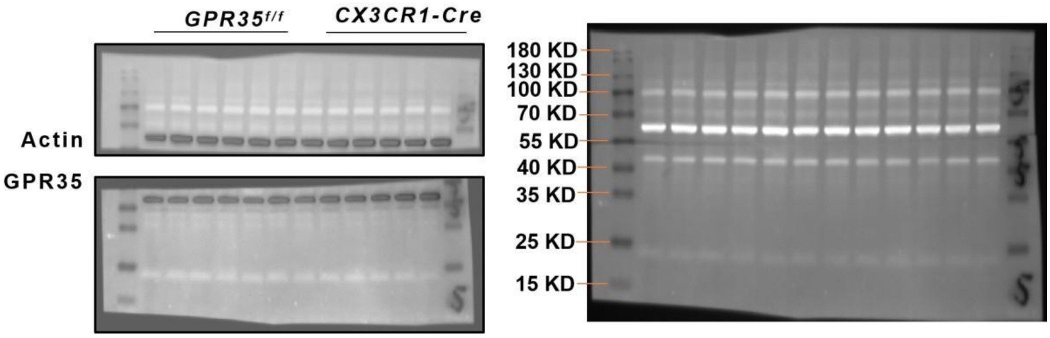

Supplementary Figure S13.

Kidney

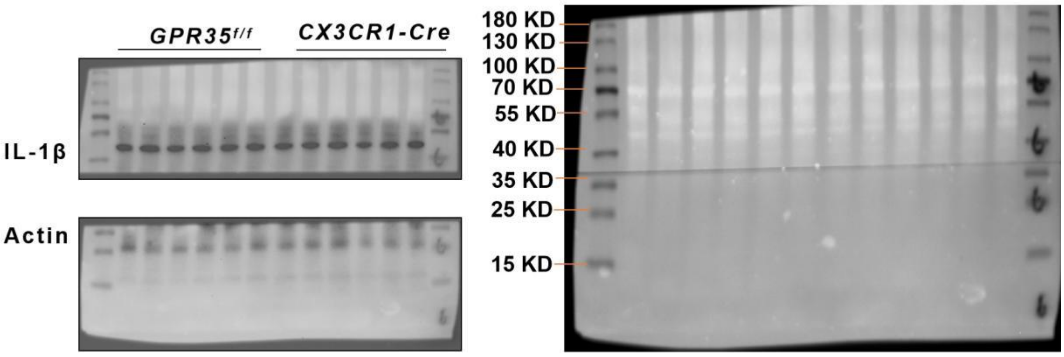

Supplementary Figure S13.

Kidney

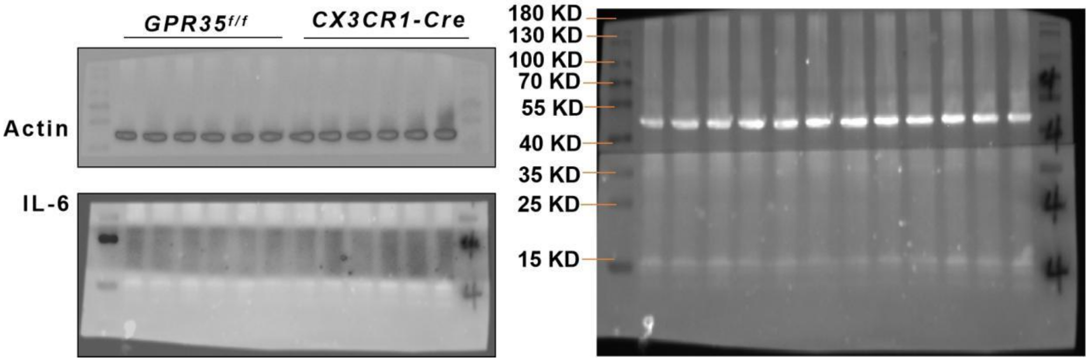

Supplementary Figure S13.

Kidney

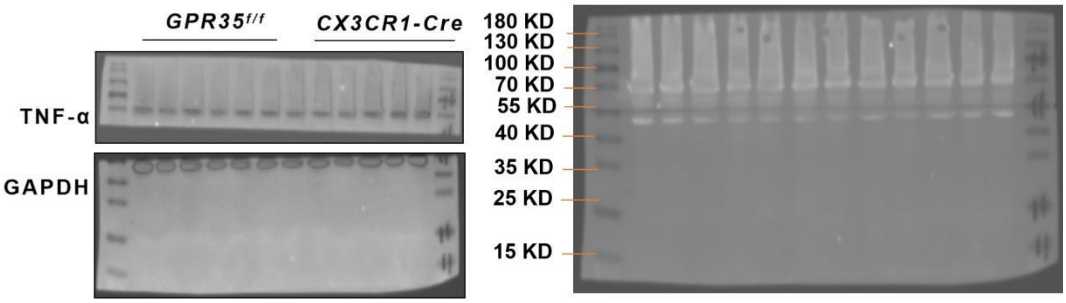

Supplementary Figure S13.

Brian

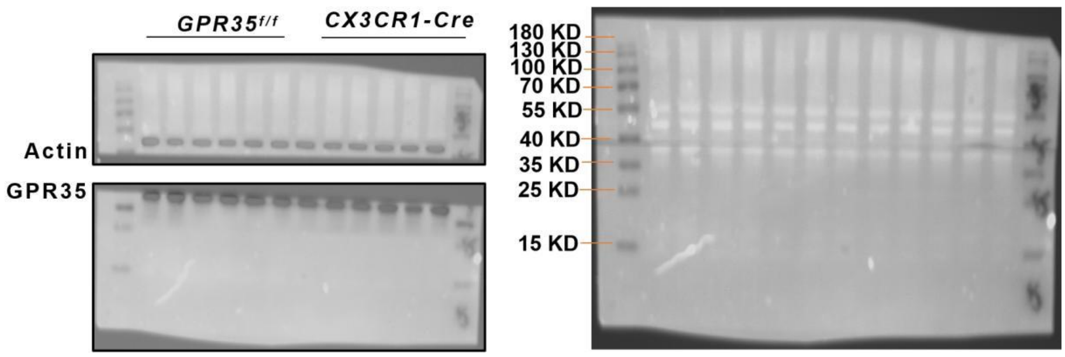

Supplementary Figure S13.

Brian

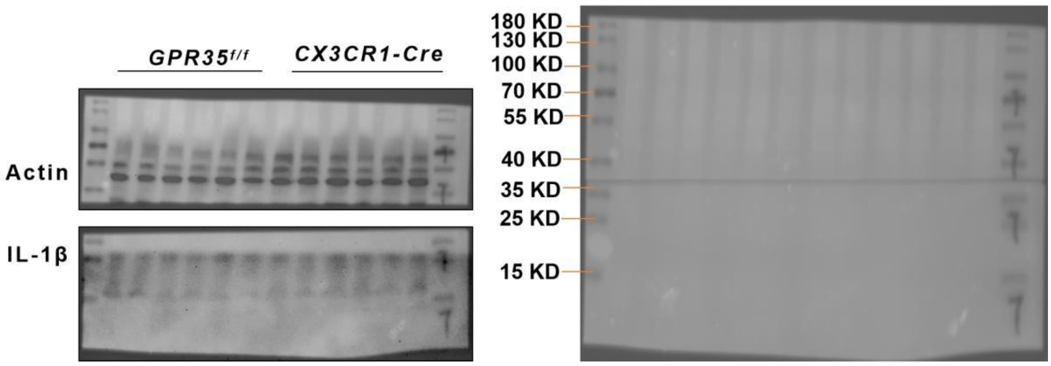

Supplementary Figure S13.

Brian

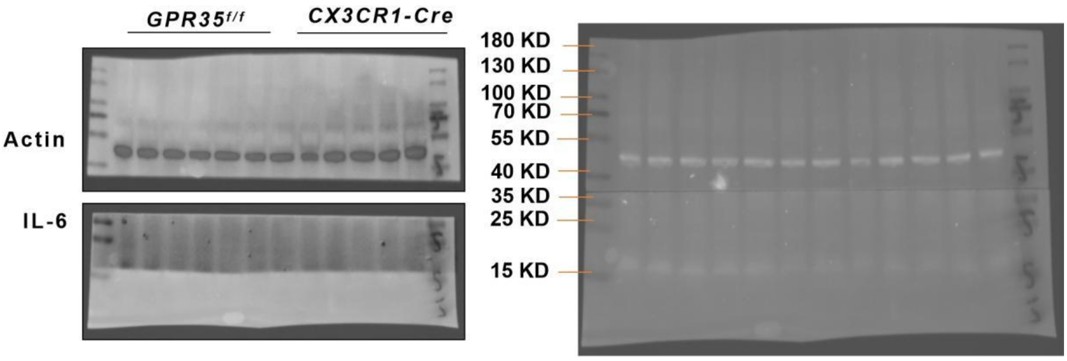

Supplementary Figure S13.

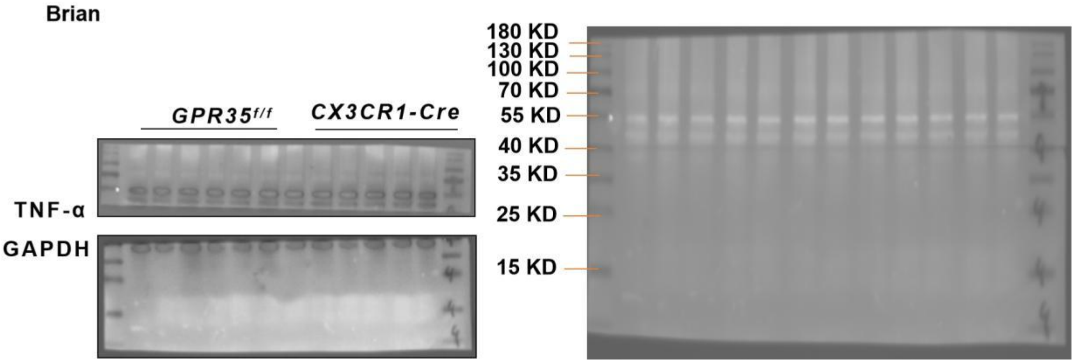

Figure 1H.

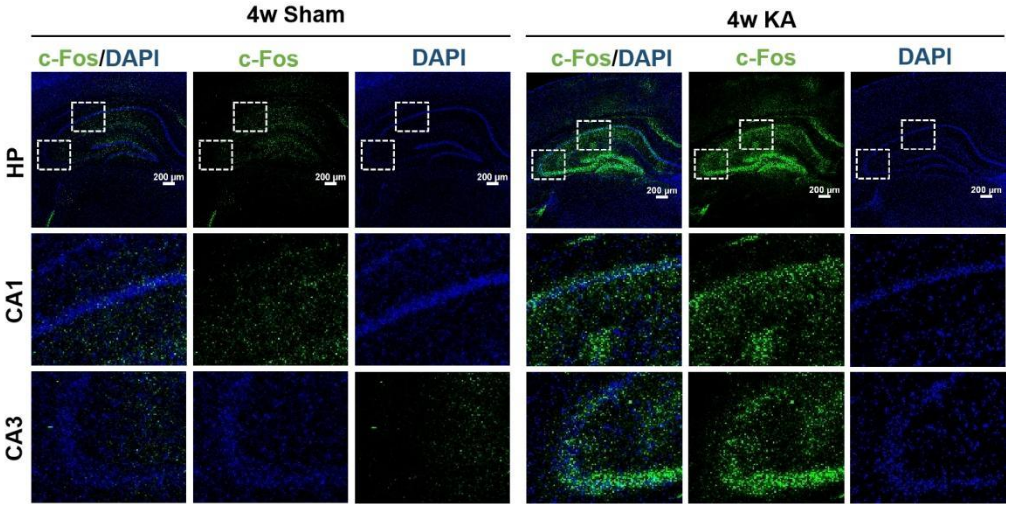

Figure 2F.

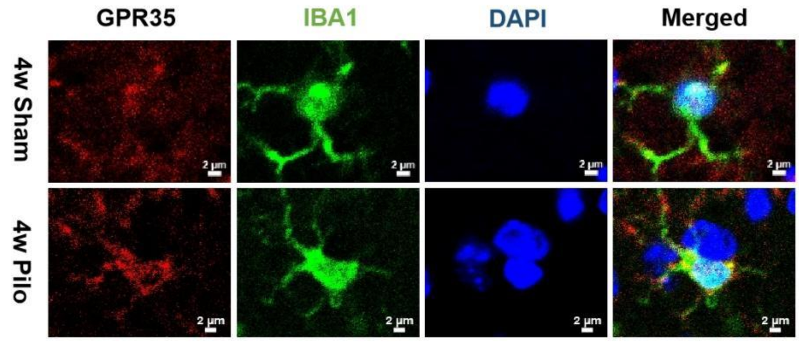

Figure 2I.

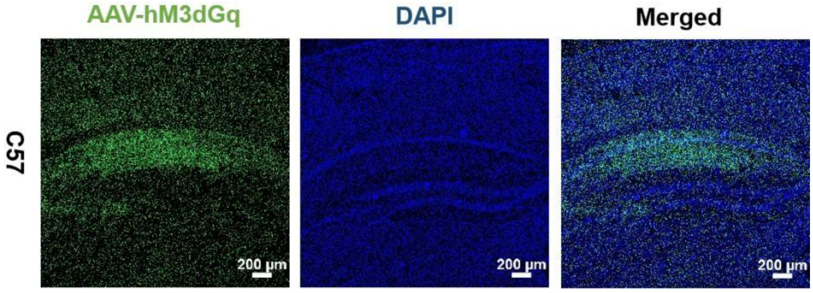

Figure 2M.

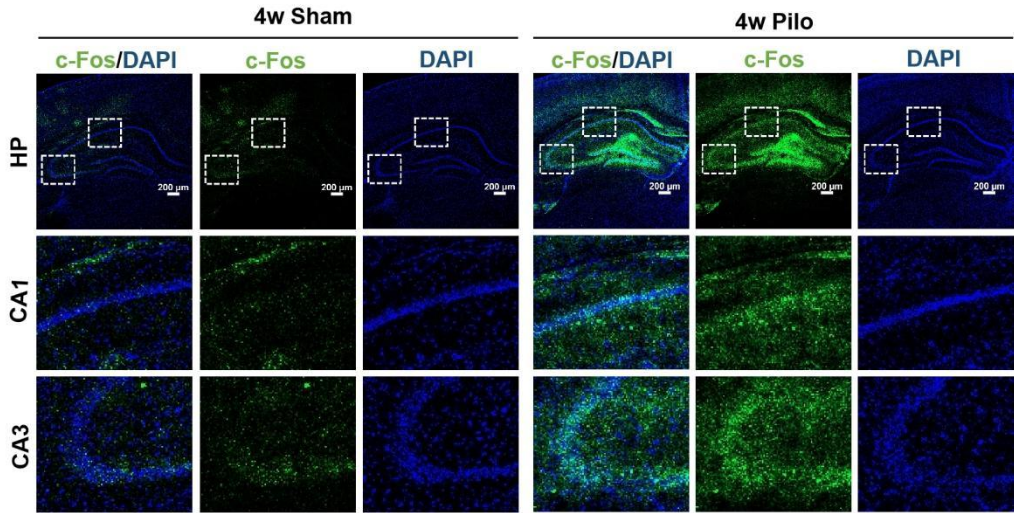

Figure 3C.

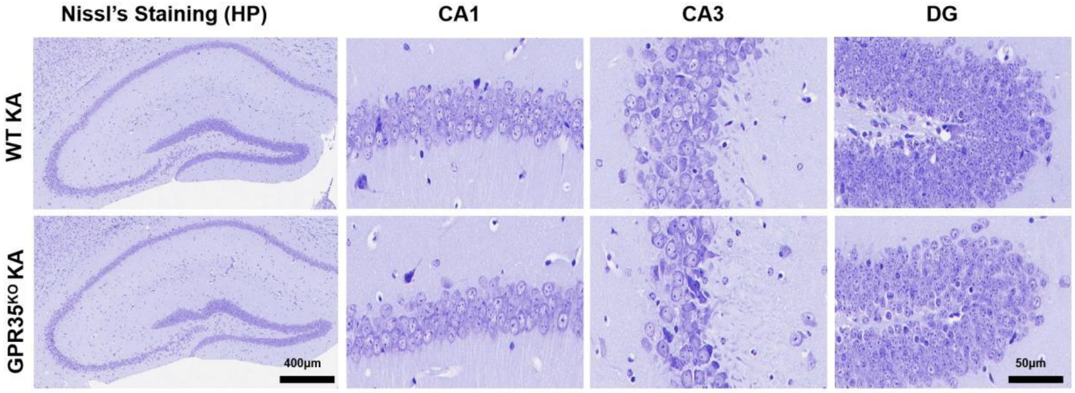

Figure 4B.

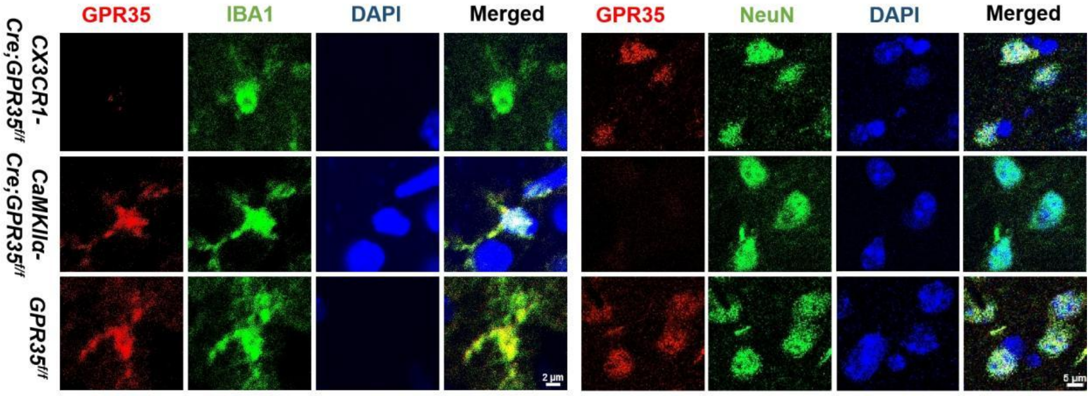

Figure 6N.

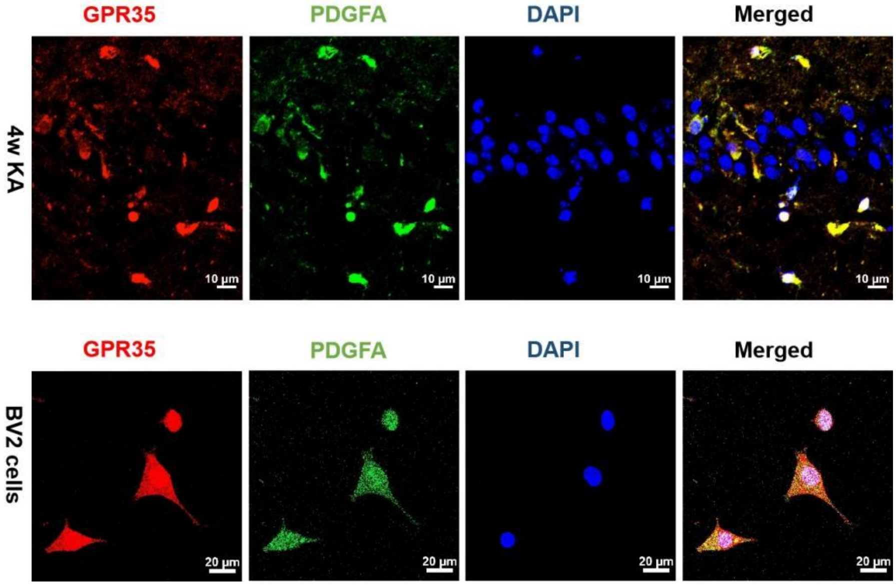

Figure 7A.

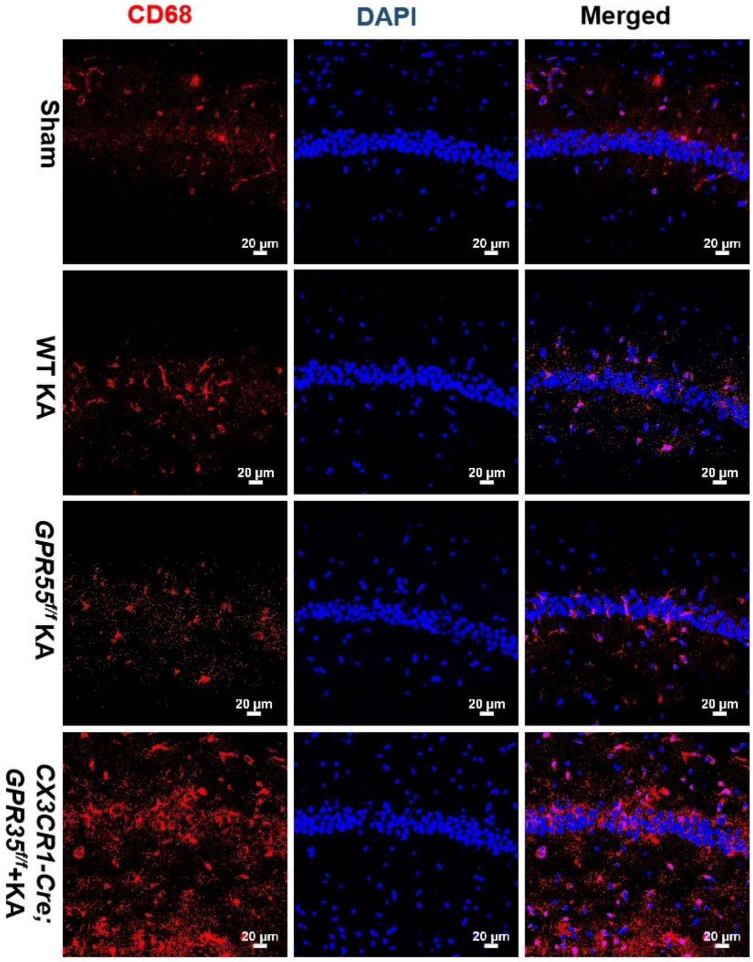

Figure 8C.

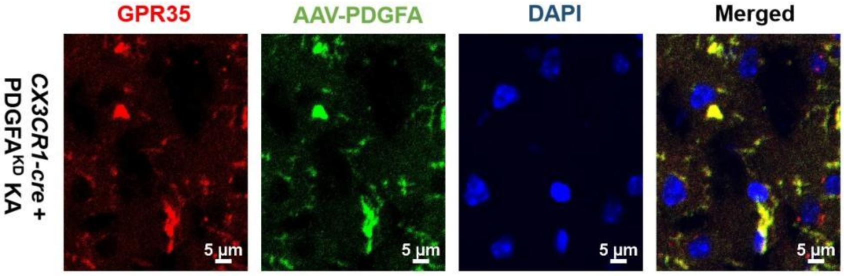

Figure 8I.

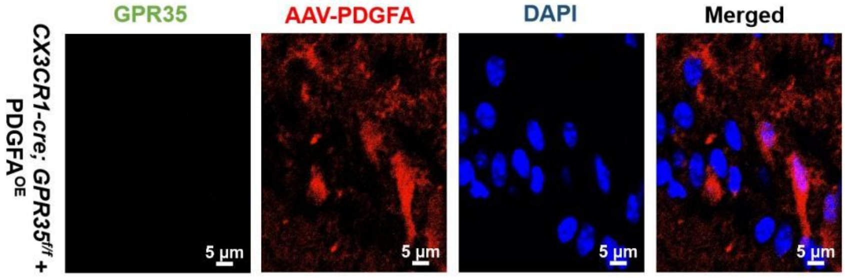

Supplementary Figure S3A .

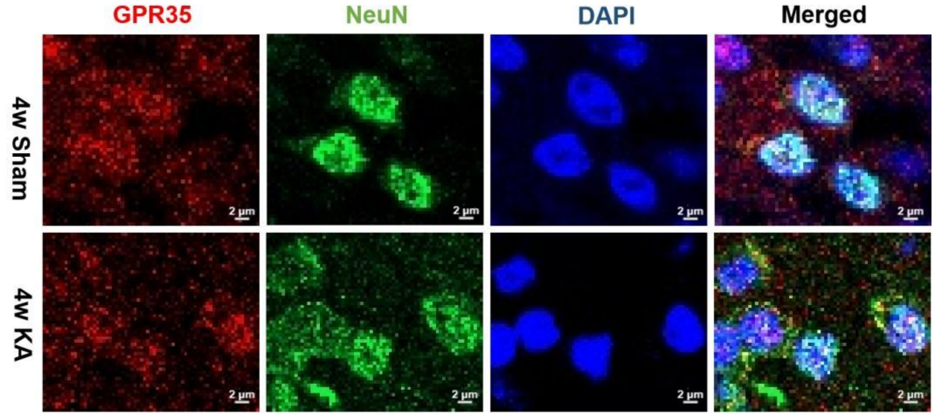

Supplementary Figure S3B .

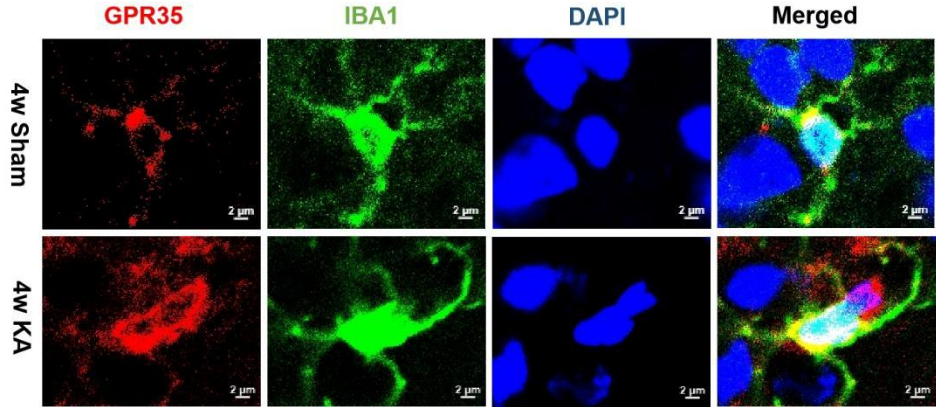

Supplementary Figure S3C .

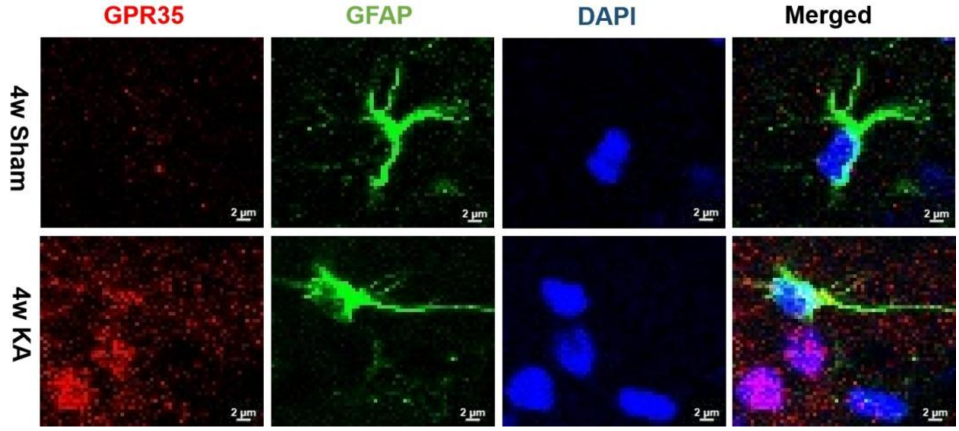

Supplementary Figure S3D .

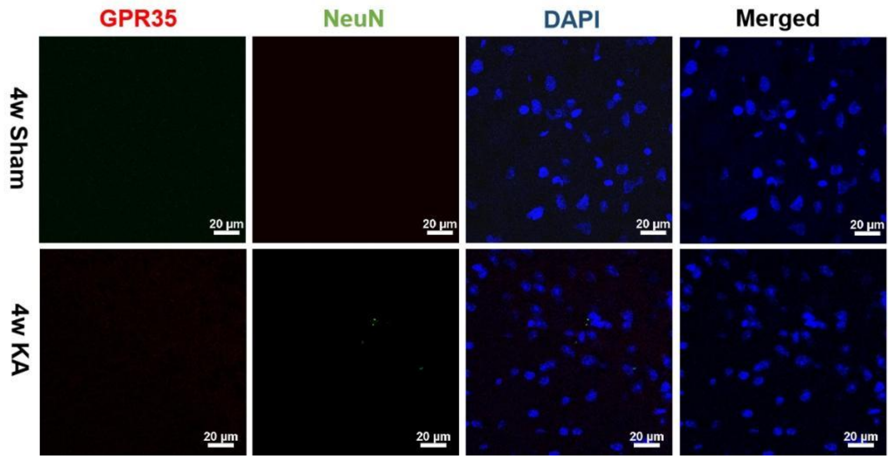

Supplementary Figure S3E .

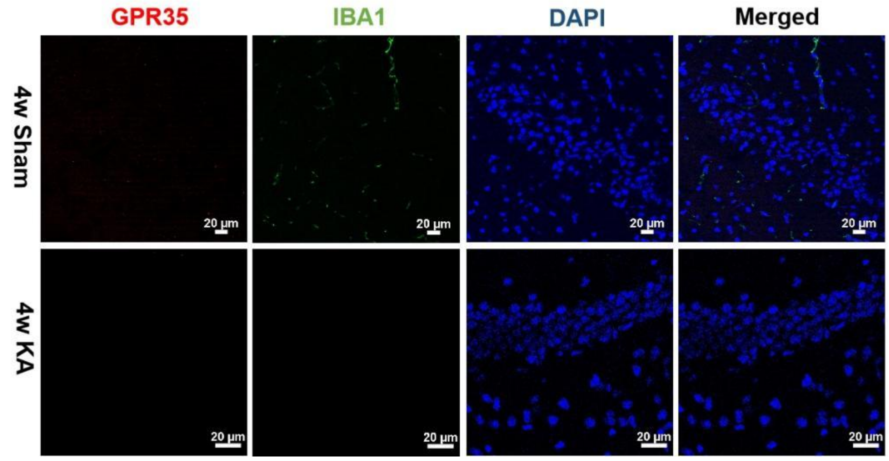

Supplementary Figure S3F .

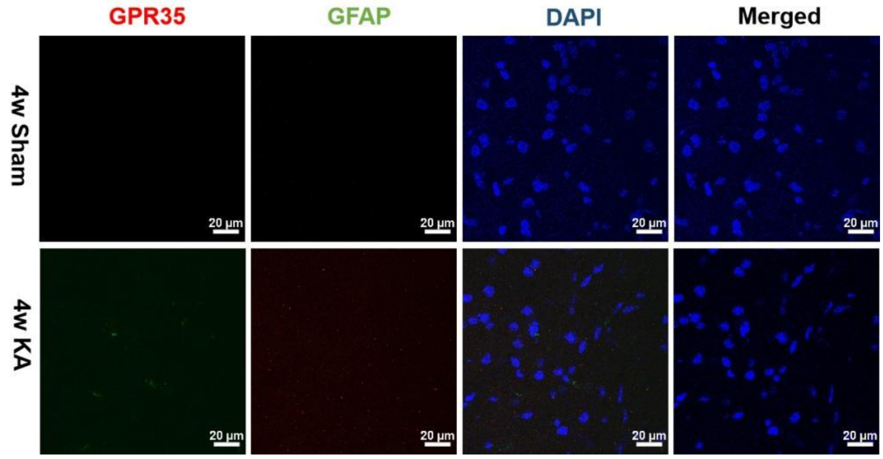

Supplementary Figure 14A.

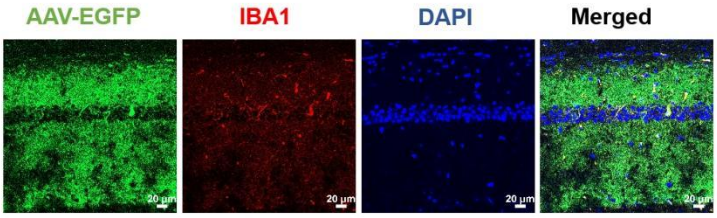

Supplementary Figure 14B.

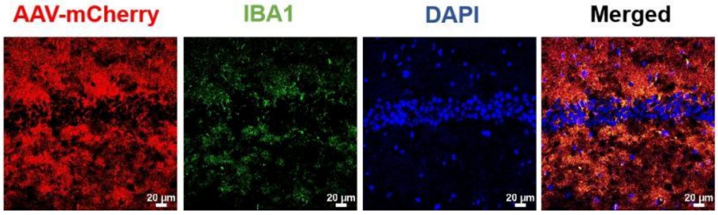

Supplementary Figure 14C.

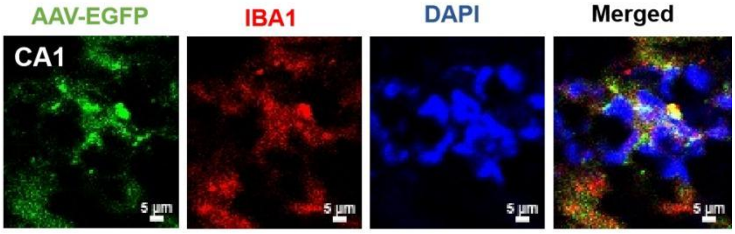

Supplementary Figure 14D.

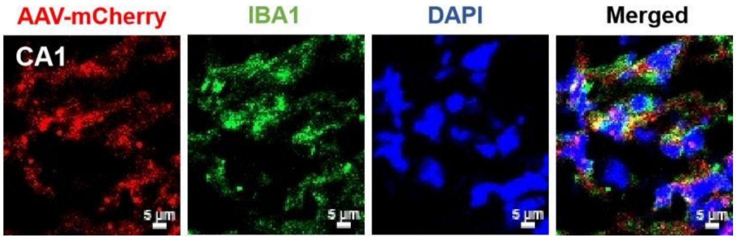

Supplementary Figure 15D.

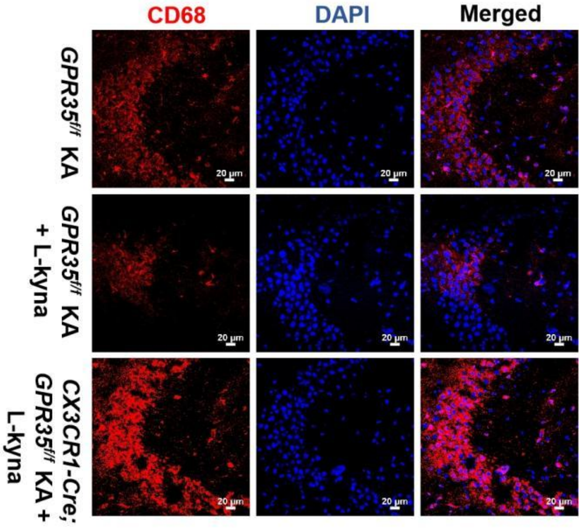

Supplement: Supplementary file 1 — Supporting File: advs74012‐sup‐0001‐SuppMat.pdf. [file ADVS-13-e19642-s001.pdf]
